# Supramolecular Assembly in Live Cells Mapped by Real-Time Phasor-Fluorescence Lifetime Imaging

**DOI:** 10.1021/jacs.4c01279

**Published:** 2024-04-19

**Authors:** Yong Ren, Zhixuan Zhou, Konrad Maxeiner, Anke Kaltbeitzel, Iain Harley, Jiaqi Xing, Yingke Wu, Manfred Wagner, Katharina Landfester, Ingo Lieberwirth, Tanja Weil, David Y. W. Ng

**Affiliations:** † Max Planck Institute for Polymer Research, 55128 Mainz, Germany

## Abstract

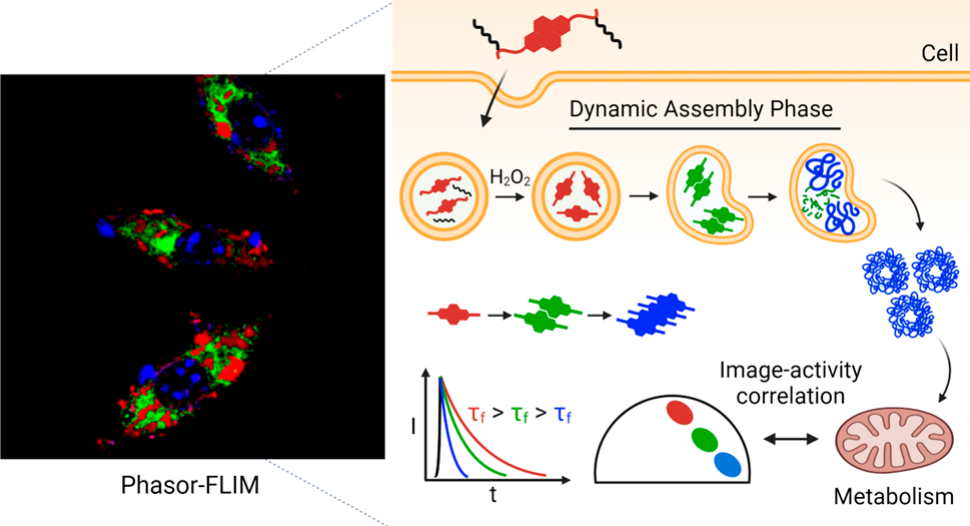

The complex dynamics and transience of assembly pathways in living systems complicate the understanding of these molecular to nanoscale processes. Current technologies are unable to track the molecular events leading to the onset of assembly, where real-time information is imperative to correlate their rich biology. Using a chemically designed pro-assembling molecule, we map its transformation into nanofibers and their fusion with endosomes to form hollow fiber clusters. Tracked by phasor-fluorescence lifetime imaging (phasor-FLIM) in epithelial cells (L929, A549, MDA-MB 231) and correlative light-electron microscopy and tomography (CLEM), spatiotemporal splicing of the assembly events shows time-correlated metabolic dysfunction. The biological impact begins with assembly-induced endosomal disruption that reduces glucose transport into the cells, which, in turn, stymies mitochondrial respiration.

## Introduction

The autonomous assembly of molecules to form nanoscale structures and aggregates in biology is a complex multiscale challenge that is poorly understood. Every structural precursor molecule within the human cell is chemically processed at different time points, and their subsequent accumulation creates a variety of supramolecular intermediates that evolve over time.^1−4^ As a result, many intermediate species exist only transiently, and their biological consequences observed at a later time point are no longer traceable. This lack of knowledge limits progress in understanding assembly-driven biological functions and the development of targeted medical solutions. Therefore, a platform that captures molecular-level assembly dynamics and localization in real time is urgently needed.

**Figure 1:**
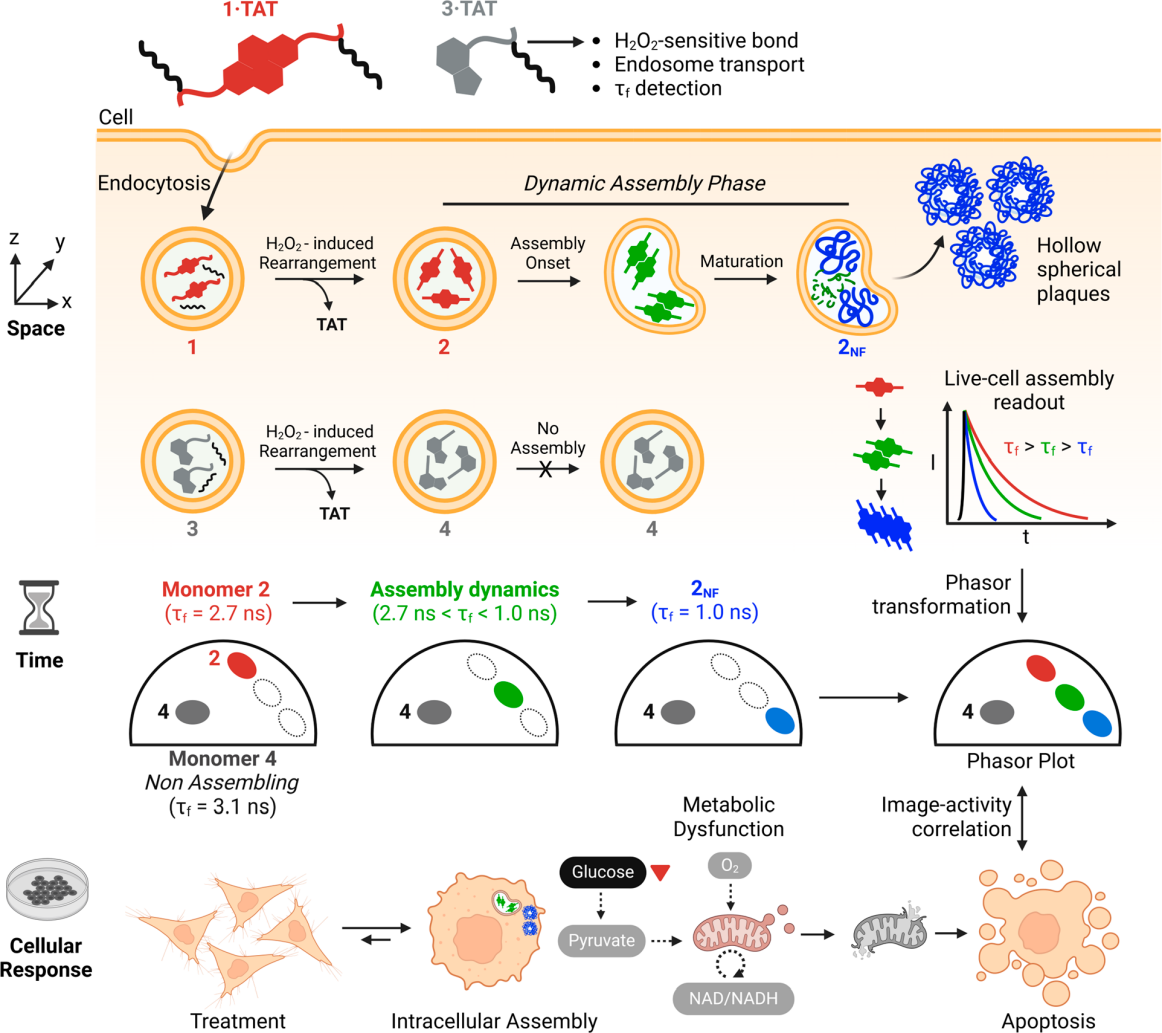
Schematic illustration of endosomal assembly of peptides traced via phasor-FLIM and real-time bioactivity correlation. Pro-assembling peptide **1·TAT** (τ_f_ = 2.7 ns) entered epithelial cells via TAT-mediated endocytosis and was converted to the supramolecular monomer **2** by an endogeneous H_2_O_2_ switch. Endosomal confinement directs the assembly of nanofibers to fuse and form discrete hollow clusters **2**_**NF**_ (τ_f_ = 1.0 ns). The assembly dynamics are observed in real time as photons move between 2.7 ns and τ_f_ > 1.0 ns, correlating to the extent of metabolic dysfunction.

Synthetic molecules serve as important tools to gain fundamental insights of cellular responses and biological pathways without the need for genetic engineering.^5−7^ However, when crossing from discrete oligomers to the formation of higher ordered structures and aggregates, it is usually not possible to resolve the dynamics within the early phase of molecular assembly.^8−11^ To unravel this elusive phase, we have integrated chemical switches, real-time imaging technologies, and biological readouts into a short peptide amphiphile that is programmed to undergo intracellular assembly (Figure 1). The assembly into nanostructures is reported live by monitoring fluorescence lifetime (τ_f_) changes of the diethoxynaphthyl diimide (NDI) moiety on the peptide. Unlike intensity- or wavelength-based sensors that require specific chromophores,^12−14^ the fluorescence lifetime response as a function of molecular aggregation is a universal property. We combine phasor plot analysis with fluorescence lifetime imaging (phasor-FLIM),^15−17^ to map assembly progression within the cell over space and time. Based on these dynamic insights, critical aggregation time points are easily identified for high-resolution correlative light-electron microscopy/tomography (CLEM) to visualize nanoscale morphologies and endosomal interactions.

By coupling imaging technologies with time-resolved extracellular flux analysis, we found that the onset of structure formation impaired endosomal functions, signaling the depletion of glucose-dependent metabolic pathways and subsequent mitochondrial dysfunction. Over time, the production of nanofibers fused with the endosomes, maturing into hollow, spherical clusters that are ejected from the apoptotic cell. We show that our integrative approach provides the breadth of technologies required to track the complex supramolecular dynamics in situ, enabling a comprehensive understanding of bioassembly processes at the molecular level.

## Results and Discussion

### Design, Synthesis, and H_2_O_2_-Induced Transformation of Pro-assembling Peptide

**Figure 2:**
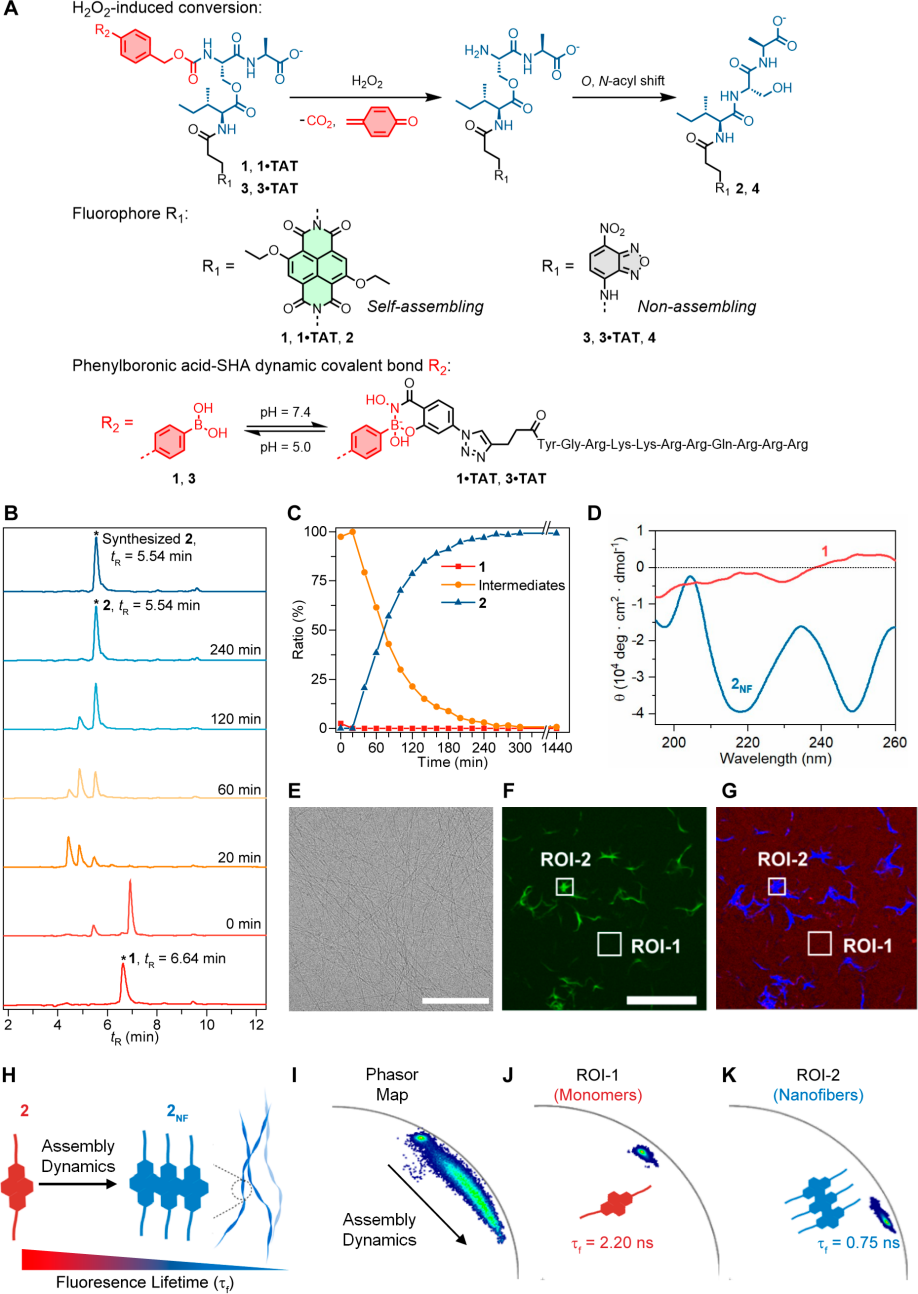
Self-assembly analysis of nanofiber **2**_**NF**_. (A) Structure of the compounds **1** and **3**, their TAT-functionalized complexes **1·TAT** and **3·TAT** obtained by salicylhydroxamic acid (SHA)-PBA dynamic covalent interaction with SHA-TAT, and their conversion products **2** and **4**. The formation of the SHA-PBA bond exhibited no apparent influence on the conversion properties of the parent compounds. (B) Kinetic analysis over the H_2_O_2_ (1.0 mM)-induced linearization of **1** (50 μM) to **2** in a mixture of NH_4_HCO_3_ buffer (pH 7.4, 10 mM) and THF (v:v = 1:1) by using HPLC-MS. (C) Molar ratio of precursor **1**, deboronation and rearrangement intermediates, and **2** after incubating with H_2_O_2_ based on the peak integration at 254 nm. (D) Circular dichroism (CD) spectra of **1** and **2**_**NF**_ in a mixture of PB (pH 7.4, 100 mM) and CH_3_CN (0.5 vol %). (E) Cryo-TEM micrograph of the nanofibers **2**_**NF**_ (100 μM) in DPBS. Scale bar: 100 nm. (F) Intensity-based confocal image for **2**_**NF**_ in a cell-free condition. λ_ex_ = 469 nm, λ_em_ = 500–520 nm. Scale bar: 20 μm. (G) Corresponding fluorescence lifetime image for **2**_**NF**_. λ_ex_ = 469 nm. The red channel indicates species with τ_f_ = 2.20 ns, and the blue channel indicates species with τ_f_ = 0.75 ns. Scale bar, 20 μm. (H) Schematic illustration of the assembly progression from monomers to assemblies, resulting in decreased fluorescence lifetime (2.20 ns → 0.75 ns) upon formation of nanofiber morphology. (I) Fluorescence lifetime analysis of assembly progression in a cell-free system using phasor analysis. Two major species showing τ_f_ values of 2.20 and 0.75 ns can be identified. (J) Phasor plots of ROI-1 (monomers) in Dulbecco’s modified Eagle medium (DMEM), τ_f_ = 2.20 ns. (K) Phasor plots of ROI-2 (assemblies) in DMEM, τ_f_ = 0.75 ns.

To enable the NDI for τ_f_ observation in the visible range, we modify the NDI core with two ethoxy groups occupying the 2,6-bay positions. The diimide positions are covalently linked to tripeptide Ile-Ser-Ala, where the design is inspired from short self-assembling peptides known in the literature.^18−21^ The serine residue, caged by a phenylboronic acid (R^2^), serves as an O → N intramolecular acyl migration switch that can be turned on by intracellular H_2_O_2_. Structural propagation occurs upon the triggered alignment of the molecular backbone between the NDI core (π interactions)^22^ and the tripeptides (van der Waals, H-bonds).^23−25^ Compound **1** and its H_2_O_2_-induced, rearranged product **2** (as a control) were synthesized using Fmoc solid-phase peptide synthesis to install the NDI moiety (Figure 2A, Figures S1–S8). HPLC-MS investigation on the conversion of compound **1** demonstrated the kinetics of the chemical switch, which is eventually transformed into **2** with 97% conversion (Figure 2B,C, Figure S21). In the absence of H_2_O_2_, **1** remained intact after 24 h (Figure S22). NBD-functionalized compound **3** and its H_2_O_2_-induced conversion product **4** were also synthesized as non-assembling control compounds (Figure 2A, Figures S1 and S9–S16). The H_2_O_2_-induced conversion of **3** to **4** was consistent with that of **1** to **2** (Figure S27). To transport peptides **1** and **3** into cells via endosomal uptake, the transactivator of transcription (TAT) derived from HIV,^26^ functionalized with a salicylhydroxamic acid (SHA) motif (SHA-TAT), was designed. TAT-functionalized complexes (**1·TAT**, **3·TAT**) were obtained through a dynamic covalent bond between the SHA moiety and the phenylboronic acid (PBA) group present in compounds **1** and **3** at pH 7.4 (Figure 2A, Figures S17–S20).^27^ These SHA-PBA bound forms (**1·TAT**, **3·TAT**) show similar responsiveness to H_2_O_2_ as their parent compounds (**1**, **3**) (Figures S23–S25, S27–S31).^21,28^

Optical properties of **1** and **2** revealed similar absorbance and fluorescence fingerprints, implying that the chemical transformations do not affect their basic photophysical behavior. In detail, **1** displayed a low-energy absorption band centered at 468 nm, assigned to the n−π* transition of the NDI group.^29^ A higher-energy absorption band was observed at 340 nm, representing the π–π* transition (Figure S32).^29^ Upon excitation of the n−π* transition at 468 nm, both **1** and **2** exhibited a broad emission band centered at around 510 nm, characteristic of NDI chromophores (Figure S32).^30^ As peptide **2** is the active self-assembling species, it exhibited a slightly lower absorption intensity and a minor hypsochromic shift in the absorption and fluorescence emission relative to **1**. These observations can be attributed to the enhanced exciton migration in the closely packed NDI cores,^29,31^ providing first hints toward assembly. Control compounds **3** and **4** exhibited nearly identical absorption bands centered at 475 nm and emission centered at 540 nm, suggesting no assembly behavior for either compound (Figure S32).

### Assembly Profile and Phasor-FLIM Calibration

At concentrations ranging from 10 to 100 μM in Dulbecco’s phosphate-buffered saline (DPBS, pH 7.4), peptide **2** assembled into nanofibers (Figure 2E and Figures S33, S35). The assembled form of **2** is henceforth labeled as **2**_**NF**_. The critical assembly concentration was assessed by Nile Red assay to be around 9 μM (Figure S36). In situ H_2_O_2_-triggered transformation of **1** (10–100 μM) further confirmed the formation of **2**_**NF**_ as the final state (Figure S34). The conversion and assembly process were supported by dynamic light scattering (DLS) analysis (Figures S37, S38). In the absence of H_2_O_2_, compounds **1**, **1·TAT**, **3**, **3·TAT**, and **4** exhibited no assembly behavior (Figure S37). Circular dichroism (CD) spectroscopy analysis on **2**_**NF**_ revealed a strong negative signal at 250 nm corresponding to the π → π* transition of the aromatic core,^30^ reflecting the importance of the NDI core during assembly. Additionally, a strong negative n → π* transition of the carbonyl group was observed at around 220 nm, indicating the H-bond interactions of the peptide backbone (Figure 2D).^32^ As a control, **1** showed no obvious signal between 200 and 260 nm. Variable-temperature (293–353 K) ^1^H NMR studies support the formation of intermolecular bonds as the basis of the structural assembly of **2**_**NF**_ (Figures S40, S41).

The fluorescence lifetime profile toward the assembly of **2** is calibrated under cell-free conditions using Dulbecco’s modified Eagle medium (DMEM) and imaged using phasor-FLIM. Intensity-based fluorescence shows large regions of nanofibers upon the assembly of **2** into **2**_**NF**_ (Figure 2F). By separating the lifetimes of emitted photons on a phasor plot, the population of photons corresponding to their spatial distribution was gathered (Figure 2F–I, Figures S42, S43). Regions containing the nanofibers (ROI-2) show a distinct locus on the phasor plot, with an average lifetime of 0.75 ns (Figure 2K, Figures S42e, S43e), whereas free molecules of **2** (ROI-1) in solution emit photons with a longer lifetime of 2.2 ns (Figure 2J, Figures S42d, S43d). The image pixels corresponding to the assembly dynamics are linearly distributed across these two phasors (Figure 2I, Figures S42c, S43c). Pixels found closer to the 0.75 ns locus imply a larger photon population corresponding to the fiber assembly. Using these parameters, live observation of the transformation of **1·TAT** to **2**_**NF**_ in the presence of H_2_O_2_ showed increasing population of photons with shorter lifetimes and their spatial correlation toward the assemblies (Figure S44, Video 1).

### Spatial Visualization of **2**_**NF**_ Using CLSM and CLEM Tomography in Cells

**Figure 3:**
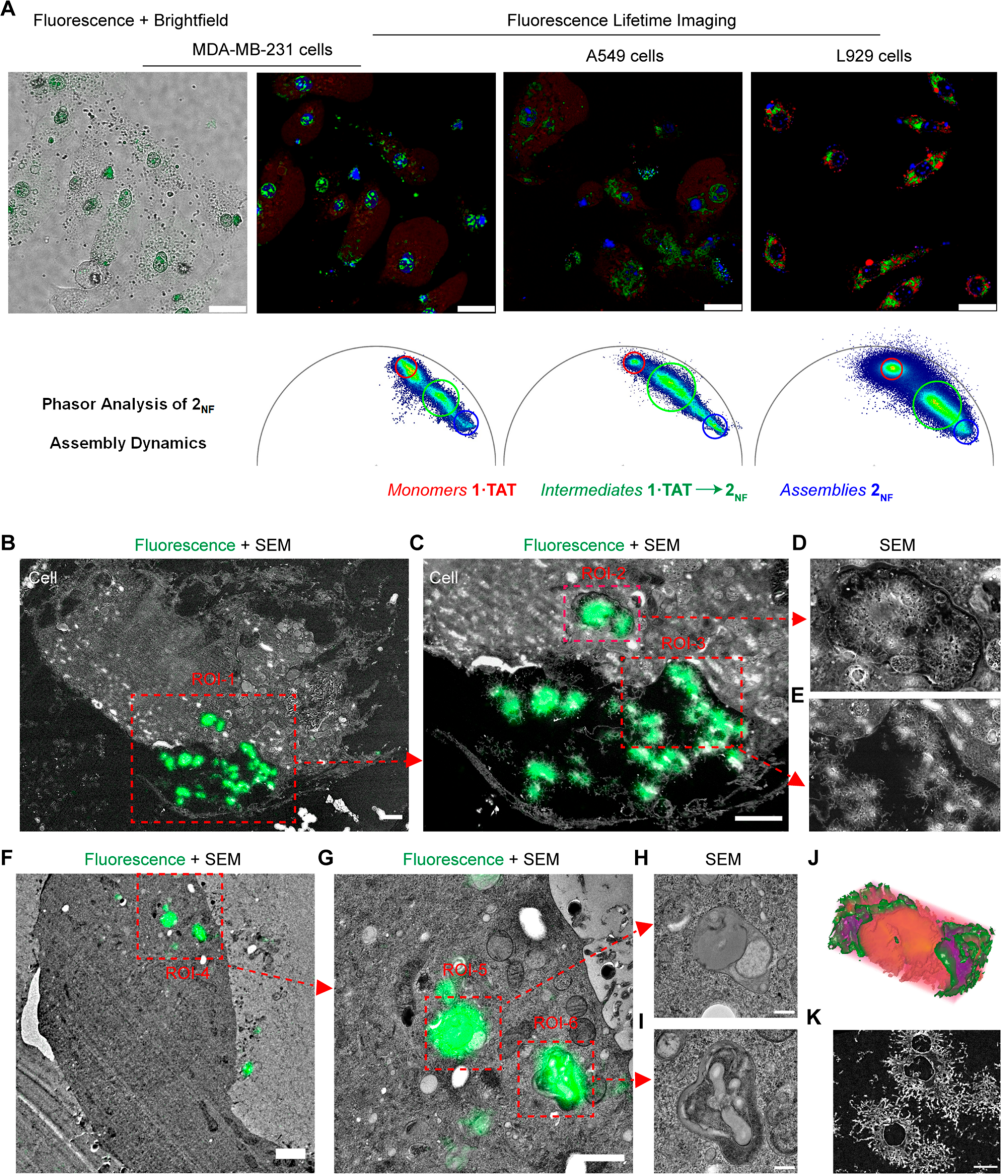
| Phasor-FLIM and CLEM analysis of the formation of hollow nanofiber clusters of **2**_**NF**_. (A) Live-cell fluorescence lifetime images of MDA-MB-231 cells, A549 cells, and L929 cells treated for 4 h with **1·TAT** (25 μM) and corresponding phasor analysis of assembly progression. Scale bars: 20 μm. (B) CLEM-SEM micrographs of MDA-MB-231 cells treated for 1 h with **1·TAT** (25 μM), showing the nanostructures of the fiber clusters as the cell shrinks. Scale bars: 2 μm. (C) Magnified CLEM-SEM micrographs correspond to ROI-1, scale bar: 2 μm. (D, E) Magnified SEM micrographs corresponding to ROI-2 and ROI-3. (F) CLEM-SEM micrographs of MDA-MB-231 cells with developing nanostructures within distorted endosomes. Scale bars: 1 μm. (G) Magnified CLEM-SEM micrographs correspond to ROI-4, scale bar: 1 μm. (H, I) Magnified SEM micrographs corresponding to ROI-5 and ROI-6, showing the phase separation and endosomal distortion brought about by the assembly dynamics. Scale bar: 200 nm. (J) 3D volume EM tomogram of the hollow nanofiber clusters, where green and red represent peptide structures and less dense areas, respectively. Purple represents spatially overlapping regions of green and red. (K) SEM micrographs of the nanofiber clusters featuring the hollow core. Scale bar: 500 nm.

The TAT-dependent endocytosis into MDA-MB-231 metastatic breast cancer cells was visualized using confocal laser scanning microscopy (CLSM) (Figure 3A, Videos 2, 3). At 4 h of treatment using 25 and 50 μM **1·TAT**, concentration-dependent assembly into aggregates within the cells occurs along with apparent cell shrinkage and blebbing (Figure S45). To overcome the limitation of intensity-based fluorescence microscopy in resolving assembly states, we performed corresponding imaging in the lifetime domain. Phasor plot analysis shows the assembly profile (molecules, intermediates, aggregates) and lifetime mapped to their spatial localization at this time point (4 h; Figure 3A). Attempts to control aggregate formation were subsequently performed with lower concentrations (5 and 10 μM of **1·TAT**) and for longer incubation times of 12 and 24 h. At 12 h, photons that are emitted intracellularly exhibit fluorescence lifetimes of 2.7 ns > τ_f_ > 2.0 ns, correlating to the initial phase of self-assembly (Figure S46). Upon further incubation to 24 h, cells remained healthy, and phasor plots reveal that further progression into fibrillar aggregates was not observed. Photon population analysis implies that at these low concentrations the cells are able to maintain homeostasis in a way that assembly intermediates do not accumulate enough to propagate into nanofibers. Control experiments with **3·TAT**, which correspondingly forms nonassembling **4**, did not produce aggregates and therefore showed a single-photon population with a τ_f_ of 3.1 ns (Figures S47, S48). In alternative cell lines such as A549 lung adenocarcinoma cells and L929 murine fibroblasts (Figure 3A), applying the sensitive phasor-FLIM technique reveals differences in the variety and distribution of assembled species within each cell type.

High spatial resolution provided by CLEM and tomography revealed the morphology of the nanostructured assemblies as discrete hollow spheres, each surrounded by a corona of fibers (Figure 3B–E, J–K). In detail, fluorescence signals from the NDI core were correlated to electron micrographs of the MDA-MB-231 cells, capturing endosomal vesicles at different stages of structure formation (Figure 3B–I and Figures S49–S52). In intact healthy cells, heavily distorted vesicles with signs of intravesicular phase separation were observed, showing the initial stages of chemical transformation of **1·TAT** (Figure 3F–I and Figures S49, S50). In contrast, dying cells show the formation of iconic fibrillar spheres, and when the cell boundary contracts, these structures are extruded to the periphery, similar to what is observed in live CLSM (Figure 3B–E and Figures S51, S52, Video 2). Characterization by electron tomography demonstrated that these fibrillar spheres contain a hollow core (Figure 3J,K, Video 4, Figure S53), showing that the endosomal vesicles provided a discrete volume that spatially controls the assembly and growth of **2**_**NF**_ nanostructures.

### Temporal Analysis of Nanofiber Clusters Using Phasor-FLIM

**Figure 4:**
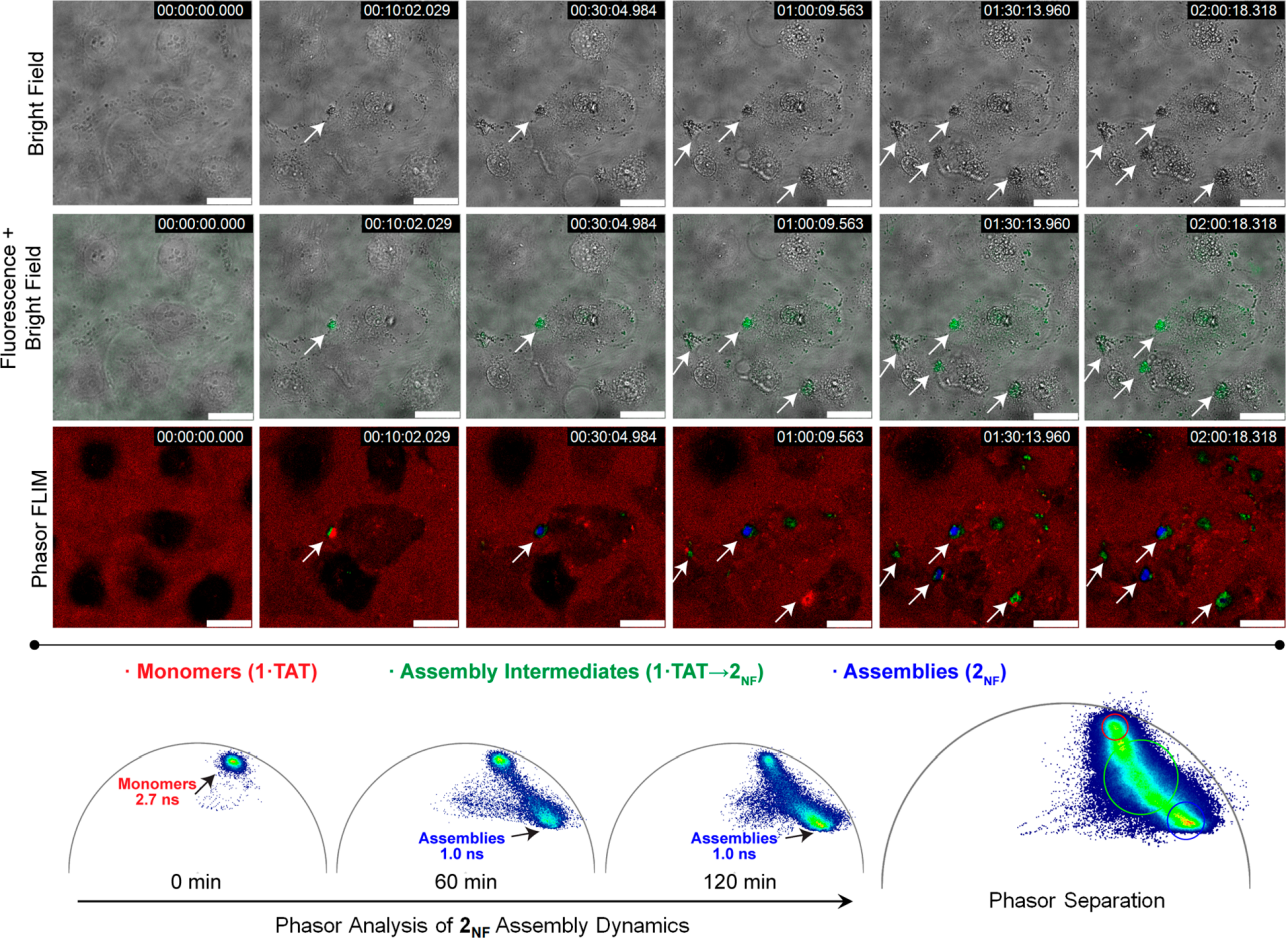
Time-lapsed phasor-FLIM analysis of **1·TAT** internalization and assembly into **2**_**NF**_ clusters. Time-dependent brightfield, fluorescence + brightfield, and phasor-FLIM images of MDA-MB-231 cells treated with **1·TAT** (25 μM) (0–120 min). Bottom row: Phasor maps of MDA-MB-231 cells treated with **1·TAT** (25 μM) at various time points as well as the total cumulative phasor distribution used to perform phasor separation. Arrows indicate the dynamics leading to nanofiber formation and growth with a concomitant change in the phasor-FLIM. Scale bars = 20 μm.

The formation of the hollow clusters was tracked using time-lapsed live cell phasor-FLIM imaging. The extent of aggregation over time can be followed as the photon population shifts between the two characteristic phasors (representing molecular **2** and assembled **2**_**NF**_) on the phasor plot. Control experiments using lysates of MDA-MB-231 cells rule out nonspecific interactions of fluorescence lifetime between **1·TAT** and cellular components (Figure S57). Immediately upon treatment of MDA-MB 231 cells with **1·TAT** (*t*_0_), emitted photons exhibit a homogeneous, single-component fluorescence decay with τ_f_ = 2.7 ns, corresponding to free molecules. With time, the cellular uptake of **1·TAT** initiated a shift of photon population toward the aggregated phasor τ_f_ = 1.0 ns (Figure 4, Video 5). The nanofiber clusters form and enlarge within 1 h, and the corresponding population of photons shifting to τ_f_ = 1.0 ns increases. From 2 h onward, the phasor plot exhibits minimal changes, suggesting that the aggregation process has been completed. Several observations, including cellular blebbing and nuclear shrinkage, which are hallmarks of cell death, were detected as aggregation progresses. In live-cell imaging, these morphological changes clearly occurred before the enlargement of the nanofiber clusters, implying that the observed biological responses were initiated at an earlier time point. Individual cells that survived treatment showed no intracellular aggregation, suggesting that these cells plausibly had higher efflux rates that prevented the accumulation of assembling precursor **2**. Hence, for each cell, the time point of cell death depends on how long it can maintain efflux to continuously expel the peptides (Video 6).

### Real-Time Biological Response upon Intracellular Assembly

The imaging studies by CLEM and phasor-FLIM showed that **1·TAT** entered the cell’s endosomes where exposure to endogenous H_2_O_2_ oxidatively cleaves the boronic acid moiety to release the assembling precursor **2** over time. Subsequent assembly and phase separation of **2** leads to the formation of hollow nanofiber clusters of **2**_**NF**_. Due to the extensive endosome distortion observed, we hypothesized that the assembly process could disable native endosomal activities.

**Figure 5:**
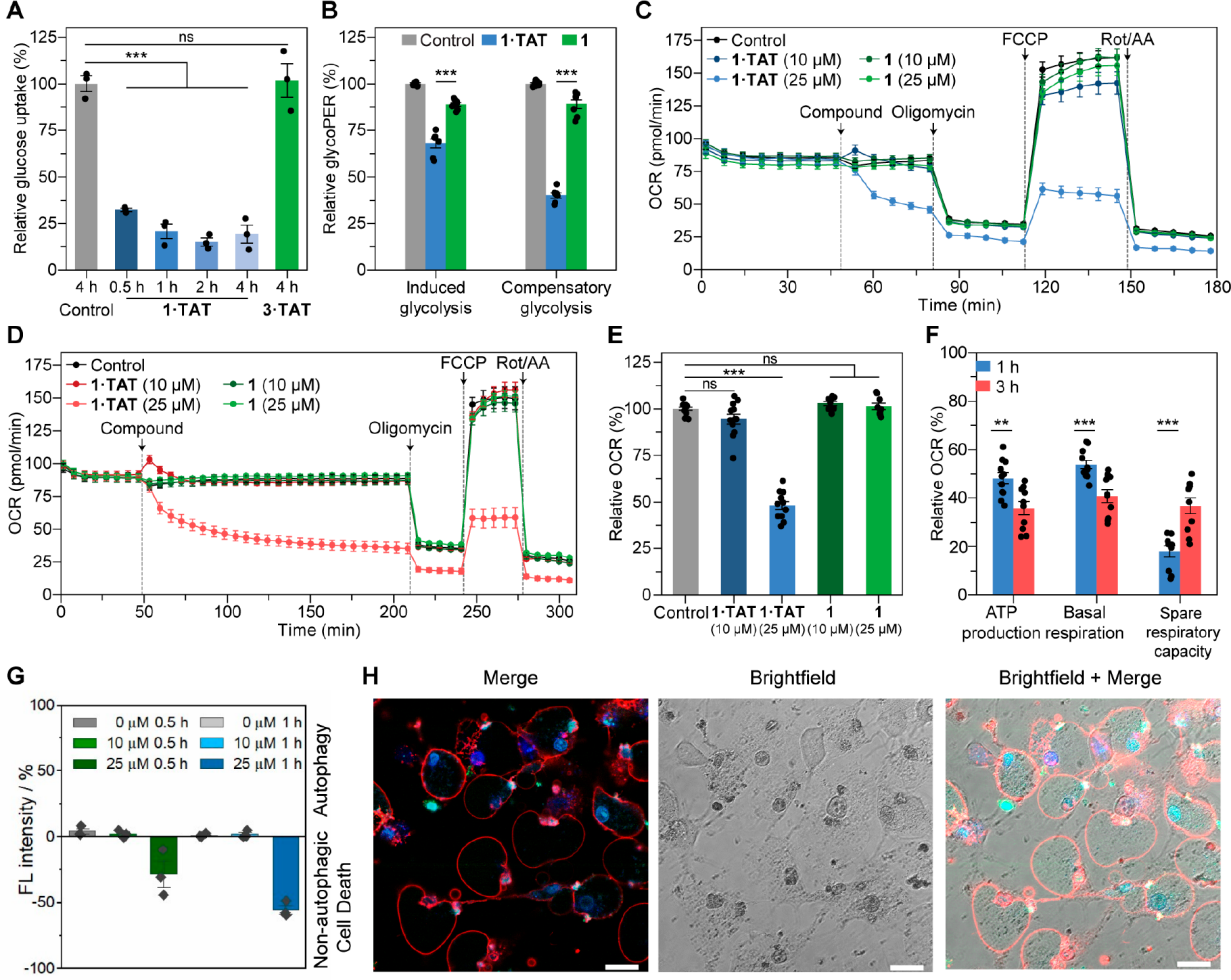
Inhibition of oxidative phosphorylation by intracellular transformation of **1·TAT** into **2**_**NF**_. (A) Glucose uptake assay based on bioluminescence detection of 2-deoxyglucose-6-phosphate upon the treatment of 2-deoxyglucose. MDA-MB-231 cells were incubated with **1·TAT** (25 μM) for various durations (0.5, 1, 2, and 4 h) and **3·TAT** (25 μM) for 4 h as a control. Analysis was performed by a luminescence read-out. *n* = 3. (B) Real-time glycolytic flux assay and analysis of MDA-MB-231 cells treated with **1·TAT** (25 μM). The glycolytic proton efflux rate (glycoPER) obtained was used to quantify glycolytic activity. Glycolytic activity with functional mitochondria is termed induced glycolysis, whereas glycolytic activity with suppressed mitochondria is termed compensatory glycolysis. *n* = 7. All data are presented as mean ± s.e.m. Statistical significance was calculated by ANOVA with a Tukey post hoc test. *p < 0.05, **p < 0.01, ***p < 0.001. (C, D) Effect of **1·TAT** and **1** on the oxygen consumption rate (OCR) of MDA-MB-231 cells after incubation for 1 h (C) and 3 h (D). FCCP: Carbonyl cyanide-4 (trifluoromethoxy)phenylhydrazone, Rot/AA: Rotenone and antimycin. (E) Comparison of the effects of **1·TAT** (10 and 25 μM) and **1** (10 and 25 μM) on cellular ATP production OCR after incubation for 1 h. *n* ≥ 9. (F) Effect of **1·TAT** (25 μM) on ATP production, basal respiration, and spare capacity of the OCR after incubation for 1 and 3 h. Values are normalized toward the untreated control group. The last measurement before the compound injection is set as 100%. *n* ≥ 9. (G) Fluorescence labeling assay of autophagosomes with relative fluorescence is presented against untreated controls. An increase in fluorescence intensity indicates formation of autophagosomes, whereas a decrease in fluorescence suggests non-autophagic cell death. Data presented as s.e.m, *n* = 3. (H) Annexin V/DAPI assay on MDA-MB-231 cells treated with **1·TAT** (25 μM). Scale bar: 20 μm.

Among their plethora of functions, endosomes are crucial in transporting extracellular materials, including essential nutrients such as glucose.^33−36^ Using glucose to probe for endosomal transport, time-lapsed quantification of glucose uptake showed that the MDA-MB-231 cells treated with 25 μM of **1·TAT** showed >65% reduction in intracellular glucose levels within 30 min, whereas controls with nonassembling **3·TAT** retain normal function even after 4 h (Figure 5A). Within the cell, glucose is metabolized through glycolysis to form pyruvate, NADH, and ATP, with a corresponding release of protons into the extracellular medium. The reduction in glucose uptake would impair glycolysis and stymie the supply of pyruvate entering the citric acid cycle in the mitochondria, thereby destabilizing the redox activity within the electron transport chain. Real-time quantification of the glycolytic proton efflux rate (glycoPER) shows a decrease of 68.1 ± 2.4% in basal glycolytic activity (Figure 5B). The impact is magnified to a decrease of 40.1 ± 1.4% when compensatory activity from the mitochondria is artificially suppressed with rotenone and antimycin A (Figure 5B).

Without sufficient pyruvate produced by glycolysis, the NAD/NADH homeostasis maintained by the citric acid cycle and electron transport chain would be disrupted.^37^ Analyses using treated cells with 25 μM **1·TAT** show a time-dependent impact on NAD^+^, with a 51.1 ± 3.3% reduction in NAD^+^ content at 4 h (Figure S59). The impact on the electron transport chain within the mitochondria was quantified by monitoring oxidative phosphorylation (OxPhos) where ATP is produced through the consumption of oxygen. Real-time monitoring of the cells upon treatment with **1·TAT** (25 μM) reveals an acutely rapid decrease of the oxygen consumption rate (OCR), corroborating with the assembly onset observed in phasor-FLIM. Inhibitors of the protein complexes within the electron transport chain were used to evaluate ATP production and mitochondrial function. Two start points for the inhibitors were selected, at 30 min and 2 h 30 min, corresponding to the dynamic assembly phase and when the formation of nanofiber clusters **2**_**NF**_ is mostly completed. Accounting for the assay time, the first impact toward mitochondria respiration was received at 1 and 3 h, respectively (Figure 5C,D). Cells treated with **1·TAT** showed concentration dependency, with 25 μM demonstrating a marked decrease in ATP production to 48.1 ± 2.3% relative to that of untreated control cells (Figure 5E). Other mitochondrial functions associated with basal (53.8 ± 1.7%, Figures S61a, S62b) and spare respiratory capacities (17.9 ± 2.2%, Figures S61b, S62c) were similarly reduced by the formation of **2**_**NF**_. No significant inhibitory effect was detected for cells treated with **1**, supporting the idea that the metabolic disruption is exerted by intracellular aggregation of **2**_**NF**_. Between the two phases of assembly (Figure 5F), the reduction in ATP production was consistent, suggesting that the size of the nanofiber clusters seems to be irrelevant in impacting mitochondrial function. In contrast, an elevated level of spare respiratory capacity (+105.6% relative to 1 h stage, 36.8 ± 3.3% relative to untreated cells) was observed at 3 h compared to treated cells at 1 h (Figure 5F). The respiratory spare capacity is defined as the difference between basal ATP production and its maximal activity, which is switched on to counter mitochondrial stress. These responses prevent the amplification of initial stress, which may otherwise further impair the respiratory chain or promote mutations in mitochondrial DNA.^38,39^

The collective impact of the assembly of **2**_**NF**_ on the cellular respiratory functions led to cell death, where treatment with **1·TAT** revealed an IC_50_ value of 69 ± 5 μM at 4 h (Figure S63). In contrast, nonassembling **3·TAT** is nontoxic up to 100 μM (Figure S64). Labeling experiments confirmed the absence of autophagosomes, ruling out mitophagy as a plausible mechanism for respiratory dysfunction (Figure 5G). Corresponding positive binding of annexin-V suggests apoptosis as the primary mechanism of cell death (Figure 5H, Figure S65).

## Conclusion

By tracking the assembly dynamics of nanostructure formation in living cells, we have demonstrated the importance of establishing a temporally and spatially resolved profile of biological activities. Within this highly transient window, when molecules begin to assemble, the phasor-FLIM technique was invaluable in resolving assembly progression in real time. The time-lapse study, in conjunction with CLEM, provided a precise rationalization of the assembly process from the molecular to the nanoscale, where precursor peptides developed into hollow nanofiber clusters under endosomal fusion. The onset of assembly was critical in the disruption of endosomal function, severely impairing the associated glucose-dependent metabolism and cellular respiration. In contrast, the growth of mature assemblies and accumulation did not induce additional biological responses. Our study paves the way to understand and visualize the supramolecular chemistry of nanostructure formation in biology to ultimately address aggregation-based dysfunction in diseases.

## Supporting Information Available

Details of instruments, materials, and methods (NMR, MS, HPLC, UV–vis), cell assays, sample preparation workflow for microscopy, microscopy parameters, setup, and analysis (PDF)
Phasor-FLIM imaging of the cell-free transformation of **1·TAT** to **2**_NF_ in the presence of H_2_O_2_ (MP4)
CLSM imaging (zoomed) of TAT-dependent endocytosis into MDA-MB-231 metastatic breast cancer cells (MP4)
CLSM imaging of TAT-dependent endocytosis into MDA-MB-231 metastatic breast cancer cells (MP4)
Video of electron tomography of fibrillar spheres (MP4)
Phasor-FLIM imaging (zoomed) of the intracellular assembly of **1·TAT** to **2**_NF_ (MP4)
Phasor-FLIM imaging of the intracellular assembly of (MP4)
