# Supplementary material for: Supramolecular Assembly in Live Cells Mapped by Real-Time Phasor-Fluorescence Lifetime Imaging
Source: J Am Chem Soc. 2024 Apr 19;146(17):11991–9. doi: 10.1021/jacs.4c01279 (PMC11066860; doi:10.1021/jacs.4c01279)
Supplement: Supplementary file 1 — ja4c01279_si_001.pdf [file ja4c01279_si_001.pdf]

Supramolecular Assembly in Live Cells mapped by Real-time Phasor-Fluorescence Lifetime Imaging

Yong Ren[1], Zhixuan Zhou[1], Konrad Maxeiner[1], Anke Kaltbeitzel[1], Iain Harley[1], Jiaqi Xing[1], Yingke Wu[1], Manfred Wagner[1], Katharina Landfester[1], Ingo Lieberwirth[1], Tanja Weil[1,*], David Y.W. Ng[1,*]

[1] Max Planck Institute for Polymer Research, 55128 Mainz, Germany

* Corresponding Author. Email: weil@mpip-mainz.mpg.de; david.ng@mpip-mainz.mpg.de

Table of Contents

1 General Information 3

1.1 Materials 3

1.2 Instruments 3

1.2.1 Nuclear Magnetic Resonance Spectroscopy (NMR) 3

1.2.2 Microwave Peptide Synthesizer 3

1.2.3 Preparative High-Performance Liquid Chromatography (HPLC) 3

1.2.4 Analytical High-Performance Liquid Chromatography (HPLC) 3

1.2.5 High-Performance Liquid Chromatography - Mass Spectrometry (HPLC-MS) 4

1.2.6 Matrix-Assisted Laser Desorption/Ionisation - Time of Flight Mass Spectrometry (MALDI-TOF-MS) 4

1.2.7 Spectroscopy 4

1.2.8 Dynamic Light Scattering (DLS) 4

1.2.9 Transmission Electron Microscopy (TEM) 4

1.2.10 Cryogenic Transmission Electron Microscopy 5

1.2.11 Cell Culture 5

1.2.12 Luminescence Detection of Cell Viability 5

1.2.13 Confocal Laser Scanning Microscopy & Fluorescence Lifetime Imaging Microscopy 5

1.2.14 Seahorse XFe96 Analyzer 6

1.2.15 Fluorescence Microscopy 6

2 Synthesis and structural characterization 7

2.1 Compound 1 8

2.2 Compound 2 11
2.3 Compound 3 13
2.4 Compound 4 15
2.5 Compound 1·TAT/3TAT 19
3 H_2O_2-induced formation of 2 22
3.1 HPLC-MS analysis of H_2O_2-induced conversion from 1/1·TAT to 2. 22
3.2 HPLC-MS analysis of H_2O_2-induced conversion from 3/3·TAT to 4. 27
4. Photophysical properties of 3 and 4 30
5. Self-assembly profiles of 2_{NF} 31
5.1 TEM analysis of the self-assemblies 31
5.2 Determination of critical aggregation concentration (CAC) 32
5.3 DLS analysis 33
5.4 Variable temperature NMR analysis of the self-assemblies 34
5.5 Fluorescence lifetime imaging of assembled fibers in a Cell-Free system 35
6 Cellular uptake of 1·TAT and 3·TAT and intracellular self-assembly of 1·TAT 37
6.1 Cellular uptake of 1·TAT 37
6.2 Electron microscopy and correlative light- and electronmicroscopy of cells 39
6.3 Tomography 43
6.4 Mapping intracellular formation of 2_{NF} for live cells using Phasor-FLIM 44
6.5 Stability of compound 1 in cell lysate 45
6.6 HPLC analysis of intracellular transformation of 1·TAT into 2_{NF} 46
6.7 Phasor-FLIM analysis of 1·TAT incubated in cell lysates of MDA-MB-231 cells 48
7. Biological response of intracellular superstructure formation 48
7.1 Glucose Uptake-Glo^{TM} Assay 48
7.2 NAD/NADH assay 49
7.3 Effect on cellular oxidative phosphorylation 50
7.4 Cell viability assay 53
7.5 Autophagy assay 54
7.6 Annexin V/DAPI apoptosis assay 54

1 General Information

1.1 Materials

Reagents and solvents were purchased from commercial sources and were used without further purification. Peptide Synthesis grade reagents were used for synthesizing the peptides. HPLC was performed using CH_3CN in HPLC grade (containing 0.1% CF_3COOH) and H_2O for HPLC (containing 0.1% CF_3COOH) and reactions was obtained from a Millipore purification system.

1.2 Instruments

1.2.1 Nuclear Magnetic Resonance Spectroscopy (NMR)

NMR spectra for all compounds and intermediates were recorded on a Bruker Avance III 700 MHz NMR spectrometer. Temperature control was achieved using a VTU (BCU II; variable temperature unit) with an accuracy of +/- 0.1K. The samples were stabilized at the desired temperature for 20 minutes prior to the measurements. For ^{1}H spectra, the chemical shifts are reported in parts per million (ppm) from high to low frequency using the residual solvent peak as the internal reference (D2O δ = 4.79 ppm and DMSO-d6= 2.50 ppm). All ^{1}H resonances are reported to the nearest 0.01 ppm. The multiplicity of 1H signals are indicated as: s = singlet; d = doublet; t = triplet; q = quartet; p = pentet; m = multiplet; br = broad; or combinations of thereof. Coupling constants (J) are quoted in Hz and reported to the nearest 0.1 Hz. Where appropriate, averages of the signals from peaks displaying multiplicity were used to calculate the value of the coupling constant. Chemical shifts for protons of the compounds were assigned on the basis of COSY and HSQC correlations. The data were processed in MestReNova.

1.2.2 Microwave Peptide Synthesizer

Peptides were synthesized in a Liberty Blue Automated Microwave Peptide Synthesizer by CEM Corporation.

1.2.3 Preparative High-Performance Liquid Chromatography (HPLC)

The compounds were purified by preparative HPLC using a setup by Shimadzu. For purification either a ZORBAX Eclipse XDB-C18 HPLC column (9.4 × 250 mm, 5 µm) was used at a flowrate of 4 mL/min or a Phenomenex Gemini 5 µm NX-C18 110 Å 150 × 30 mm was used at a flowrate of 25 mL/min. Experiments were performed using CH_3CN in HPLC grade (containing 0.1% CF_3COOH) and MiliQ water (containing 0.1% CF_3COOH).

1.2.4 Analytical High-Performance Liquid Chromatography (HPLC)

The samples were analyzed by analytical HPLC using a setup my Shimadzu. An Altlantis T4 column (4.6 × 100 mm, 5 µm) was used at a flowrate of 1 mL/min. Experiments were performed using CH_3CN in HPLC grade (containing 0.1% CF_3COOH) and MiliQ water (containing 0.1% CF_3COOH). The solvent gradient started with 100% water, then the CH_3CN content was linearly increased to 100% in 20 min. The molar ratio of the compounds was calculated using peak areas at 254 nm. Data were processed in LabSolutions and Origin.

1.2.5 High-Performance Liquid Chromatography - Mass Spectrometry (HPLC-MS)

The compounds were analyzed by HPLC-ESI-MS on a LC-MS 2020 by Shimadzu using a Kinetex 2.6 µm EVO C18 100 Å LC 50 × 2.1 mm column. MilliQ water, acidified with 0.1% formic acid and CH_3CN were used as solvents for all measurements. The solvent gradient started with 5% CH_3CN and 95% water. This solvent ratio was kept constant for 2 min, then the CH_3CN content was linearly increased to 95% in 14 min. The molar ratio of the compounds was calculated using peak areas at 254 nm. Data were processed in LabSolutions and Origin.

1.2.6 Matrix-Assisted Laser Desorption/Ionisation - Time of Flight Mass Spectrometry (MALDI-TOF-MS)

All MALDI-TOF spectra were recorded on either a rapifleX MALDI-TOF/TOF from Bruker or MALDI Synapt G2-SI from Waters. Samples were mixed with a saturated solution of the matrix S7 α-cyano-4-hydroxycinnamic acid (CHCA) in NH_4HCO_3 buffer (20 mM)/CH_3CN 1/1. Data processing was performed in mMass.

1.2.7 Spectroscopy

The UV/Vis absorption spectra for the solutions of the compounds were recorded on a Thermo Scientific™ NanoDrop 2000/2000c spectrophotometer in a Hellma high precision quartz cell, 10 x 10 mm light path. The luminescence emission spectra for the solutions of the compounds were recorded on a Cary Eclipse fluorescence/luminescence spectrophotometer in a Hellma high precision quartz cell, 10 x 10 mm light path. For the CAC analysis using Nile Red, the luminescence spectra were recorded using a Tecan Spark 20M microplate reader. CD spectra were recorded on a JASCO J-1500 spectrometer in a Hellma high precision cell with 1 mm light path. Data were processed in Spectra Analysis by JASCO and Excel.

1.2.8 Dynamic Light Scattering (DLS)

Single-angle DLS measurements were performed at 25 °C using a Malvern ZetaSizer Nano S purchased from Malvern Instruments Ltd. (Malvern, Great Britain) with a He/Ne Laser (λ = 633 nm) at a fixed scattering angle of 173°. All measurements were performed in triplicate. The obtained data was processed by cumulant fitting for D_h, or by CONTIN fitting for intensity weighted particle size distribution. Samples were prepared at 50 µM. Dust was removed prior to each measurement by filtration through GHP syringe filters (0.2 µm pore size, Acrodisc).

1.2.9 Transmission Electron Microscopy (TEM)

TEM images of the conjugate solutions were taken on a JEOL 1400 transmission electron microscope at a voltage of 120 kV. Samples were prepared on Formvar/carbon-film coated copper grids (300 mesh) by Plano GmbH. In order to prepare the TEM grids, 4 µL of the sample solution were put on freshly glow discharged (30 s at 40 W in a 6:1 oxygen: hydrogen plasma) Formvar coated copper grids. After 5 min the solution was removed using a filter paper and grids were stained with 4% uranyl

acetate for 2.5 min. The grids were washed three times with MilliQ water and dried before measuring. TEM images were processed in ImageJ.

1.2.10 Cryogenic Transmission Electron Microscopy

For cryo-TEM examination the samples were vitrified using a Vitrobot Mark V (Thermo Fisher, Hilsboro Oregon) plunging device. 3 µl of the sample dispersion was applied to a Quantifoil or a lacey carbon coated TEM grid that has been glow discharged in a 6:1 oxygen: hydrogen plasma (Diener Nano®, Diener electronic, Germany) shortly before. After removing excess sample solution with a filter paper, the grid is immediately plunged into liquid ethane. For the subsequent examination the specimen is transferred to a TEM (FEI Titan Krios G4) keeping cryogenic conditions.

Conventional TEM imaging was done using an acceleration voltage of 300 kV. Micrographs were acquired with a 4k Direct Electron Detection Camera (Gatan K3) under low dose conditions.

1.2.11 Cell Culture

MDA-MB-231 cells were cultured at 37 °C and 5% CO_2 in Dulbecco's Modified Eagle's Medium (DMEM, high glucose), supplemented with 10% FBS. Both cell lines were cultured in T75 culture flask and subcultivated two to three times per week.

1.2.12 Luminescence Detection of Cell Viability

After performing the CellTiter-Glo® Luminescent Cell Viability Assay in a half area, white 96-well plate, luminescence intensity was measured on a Promega GloMax®-Multi Detection System using the protocol for the CellTiter-Glo® Luminescent Cell Viability Assay preinstalled on the instrument. Data were processed in Excel and graphs were generated in Origin.

1.2.13 Confocal Laser Scanning Microscopy & Fluorescence Lifetime Imaging Microscopy

CLSM-FLIM imaging was performed on a STELLARIS 8 Leica DMi8 microscope (Leica Microsystems, S/N: 8300000313). The live cells imaging was conducted in a microscope Incubator (okolab).

Cells are cultured in an IBIDI 8-well glass bottom chamber at a density of 25,000 cells per well in DMEM and allowed to adhere overnight at 37°C, 5% CO_2. The medium was aspirated and samples pre-dissolved in DMEM at the respective concentrations were introduced into each well. Incubation was performed at 37°C, 5% CO_2.

Cells were imaged live using an incubator-equipped Leica Stellaris® 8 microscope (40x glycerol immersion objective) with fast lifetime contrast (FALCON) module. Samples were excited using a 40 MHz pulsed white light laser tuned to 469 nm for both intensity and fluorescence lifetime measurements. Emitted photons were detected using HyD® X (GaAsP hybrid photocathode) detector with a filter window at 480 nm – 650 nm.

Fluorescence lifetime imaging (FLIM) was conducted with living cells at 2.5 min intervals, with scanning resolution of 1024 x 1024 pixels at 200 Hz, 15 frame accumulation equipped with adaptive

focusing on each frame and well position. Acquisition was performed for 100 frames. Photons were counted based on FALCON-modified time correlated single photon counting (TCSPC) method. Each pixel is transformed into a phasor plot according to the following equation:

$$g_{i,j}(\omega) = \int_0^T I(t) \cdot \cos(n\omega t)\mathrm{d}t / \int_0^T I(t)\mathrm{d}t$$

$$s_{i,j}(\omega) = \int_0^T I(t) \cdot \sin(n\omega t)\mathrm{d}t / \int_0^T I(t)\mathrm{d}t$$

in which $g_{i,j}(\omega)$ and $s_{i,j}(\omega)$ are the *x* and *y* coordinates of the phasor plot, n and ω are the harmonic frequency and the angular frequency of excitation, respectively, and T is the repeat frequency of the acquisition. Frequency domain data acquisition from each pixel can be converted to phasor points using the following transformations:

$$g_{i,j}(\omega) = m_{i,j} \cdot \cos\left(\phi_{i,j}\right)$$

$$s_{i,j}(\omega) = m_{i,j} \cdot \sin\left(\phi_{i,j}\right)$$

in which $m_{i,j}$ and $\phi_{i,j}$ are the modulation and phase shift, respectively, of the frequency domain

measurement at pixel *i,j*. The decay from each pixel can hence be translated to a point in the phasor plot.[1]

Phasor components were identified and separated using LAS X software. Photons that lie beyond the defined phasor are excluded from the images.

1.2.14 Seahorse XFe96 Analyzer

Effects of the compound on the cell metabolism were investigated using the Seahorse XFe96 Analyzer (Agilent Technologies) measuring the Extracellular Acidification Rate (ECAR) and Oxygen Consumption Rate (OCR). Seahorse XF Glycolysis Stress Test Kit and Seahorse XF Cell Mito Stress Test Kit were used following the manufacture's protocol to determine the glycolytic and respiratory activity of the cells. Cells were seeded at various densities on an XFe 96 well plate one day prior to the assay. On the day of the assay cells were treated with different concentrations of **1** and **1·TAT**. ECAR and OCR were measured in real-time. Data were processed in Excel and graphs were generated in Origin.

1.2.15 Fluorescence Microscopy

Images of the cells after Seahorse measurement were taken using a Leica DMi8 microscope using a 10x objective by Leica. Cells were stained using NucBlue® Live ReadyProbes® Reagent and fluorescence was imaged with 350/50 nm excitation and 460/50 nm emission filter.

2 Synthesis and structural characterization

a

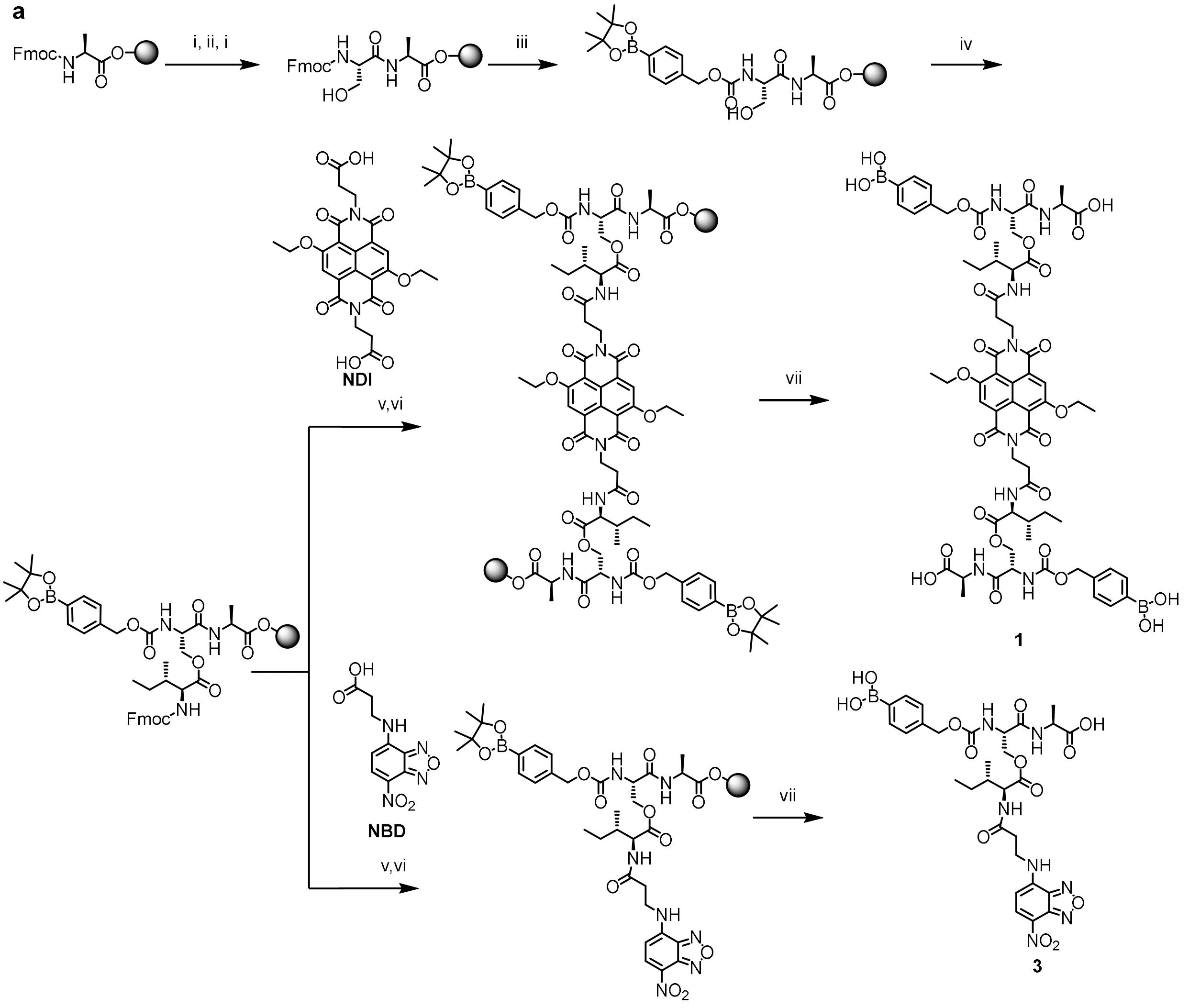

b

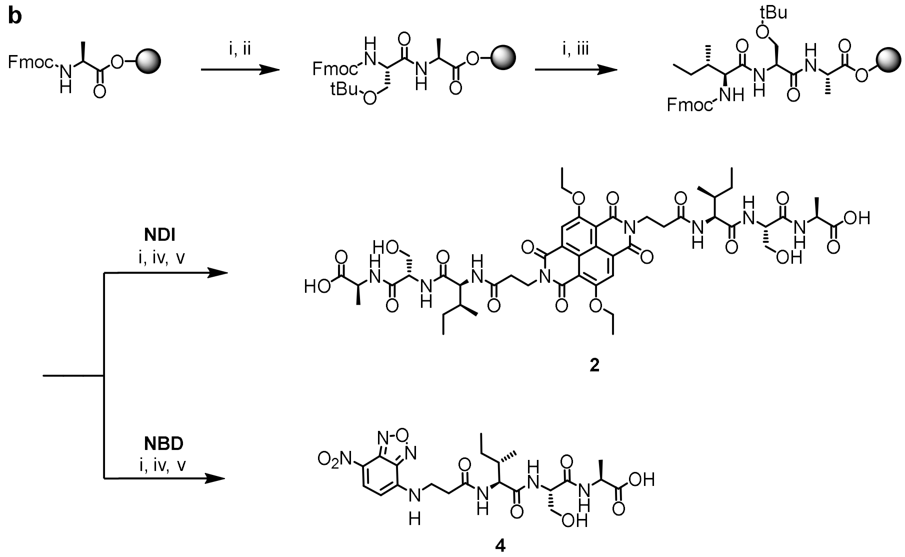

Supplementary Fig. 1| Synthesis of compounds **1**, **2**, **3**, and **4**. **a**, Synthesis of **1** and **3**. (i) 20% piperidine in DMF. (ii) Fmoc-serine, PyBOP, DIPEA, DMF, 75 °C, 20 min. (iii) 4-nitrophenyl (4-(4,4,5,5-tetramethyl-1,3,2-dioxaborolan-2- yl) benzyl), DIPEA, DMF, RT. (iv) Fmoc-Ile, DIC, 4-DMAP, RT. (v) 20% piperidine in DMF. (vi)

core-substituted NDI/3-((7-nitrobenzo[c][1,2,5]oxadiazol-4-yl)amino)propanoic acid, HATU, DIEA, DMF, RT. The c-NDI molecule was synthesized according to a previous report.[2] **b**, Synthesis of **2** and **4**. (i) 20% piperidine in DMF. (ii) Fmoc-Ser(tBu), PyBOP, DIPEA, DMF, 75 °C, 20 min. (iii) Fmoc-Ile, PyBOP, DIPEA, DMF, 75 °C, 20 min. (iv) core-substituted NDI/3-((7-nitrobenzo[c][1,2,5]oxadiazol-4-yl)amino)propanoic acid, HATU, DIPEA, DMF, RT, overnight. (v) 95% CF_3COOH, 2.5% TIPS, 2.5% H_2O.

2.1 Compound 1

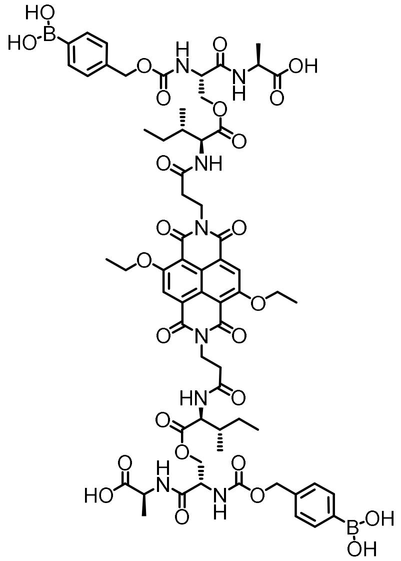

Synthesis: Compound **1** was synthesized using the Fmoc solid phase peptide synthesis strategy by Merrifield, synthesizing the peptide from *C* to *N*-terminus in a microwave assisted peptide synthesizer. Fmoc-Ala preloaded Wang resin (0.5 mmol) was swollen in DMF for 1 h before use. First, the Fmoc group was removed by two consecutive deprotection steps (2 and 5 min) with 20% piperidine in DMF (10 mL) at 75 °C (i). After deprotection, the resin was washed four times with DMF (7 mL). Fmoc-serine (5 equiv. in 10 mL DMF) was coupled to the *N*-terminus by using the activator PyBOP (5 equiv. in 4 mL DMF) and activator base DIPEA (10 equiv. in 2 mL DMF) at 75 °C for 20 min (ii). After deprotection of the Fmoc protecting group (i), the *N* terminus was modified using 4-nitrophenyl (4-(4,4,5,5-tetramethyl-1,3,2-dioxaborolan-2-yl)benzyl) carbonate (1.25 equiv.) in 10 mL DMF and DIPEA (5 equiv.) overnight at room temperature (iii). After the resin was washed with DMF and DCM, Fmoc-Ile (10 equiv. in 5 mL DMF), DIC (10 equiv.) and 4-DMAP (1 equiv.) were added to form the ester bond in the serine side chain. After the reaction mixture was stirred for 12 h at room temperature, the resin was washed with DMF and DCM, dried (iv). The Fmoc group was then removed in two deprotection steps using 20% piperidine in DMF (10 min each, 5 mL) at room temperature (v). 3,3'-(4,9-diethoxy-1,3,6,8-tetraoxo-1,3,6,8-tetrahydrobenzo[lmn][3,8]phenanthroline-2,7 diyl)dipropionic acid (0.2 equiv.) was coupled onto the *N*-terminus overnight at room temperature, using HATU (5 equiv.) and DIPEA (5 equiv.) as the coupling reagents. The resin was washed with DMF and DCM, dried (vi). The product was cleaved from the resin by using 2.5 mL of a cleavage cocktail (95% CF_3COOH, 2.5% TIPS, 2.5% H_2O). This step also removed the pinacol protecting group of the PBA. After 2 hours, the cleavage cocktail was removed in vacuo (vii). The product was purified by HPLC using the Zorbax Eclipse column at a flowrate of 4 mL/min. The gradient started with 20% CH_3CN in H_2O (+0.1% CF_3COOH) and this solvent ratio was kept for 1 min, after which the CH_3CN content was

increased to 100% in 15 min. The product **S1** eluted from the column after 11.8 min and was received as a yellow powder after lyophilization (15.2 mg, 5.1% overall yield).

^{1}H NMR (700 MHz, DMSO-d_6): δ 8.37 (s, 2H), 8.00 (d, *J* = 6.9 Hz, 2H), 7.92 (d, *J* = 7.2 Hz, 2H), 7.73 (d, *J* = 7.6 Hz, 4H), 7.62 (s, 2H), 7.28 (d, *J* = 7.9 Hz, 4H), 5.05 (s, 4H), 4.48 (q, *J* = 6.9 Hz, 4H), 4.42 – 4.36 (m, 2H), 4.33 – 4.25 (m, 10H), 4.21 – 4.14 (m, 2H), 2.64 – 2.61 (m, 4H), 1.81 – 1.72 (m, 2H), 1.51 (t, *J* = 6.9 Hz, 6H), 1.43 – 1.36 (m, 2H), 1.30 (d, *J* = 7.2 Hz, 6H), 1.16 (dt, *J* = 15.8, 7.7 Hz, 2H), 0.87 – 0.77 (m, 12H).

LCMS: LC retention time (min): 6.64. MS (ESI$^+$, *m*/*z*): 1396.9 $[M + H]^+$, 1379.6 $[M + H - H_2O]^+$, 1361.6 $[M + H - 2H_2O]^+$.

HRMS (ESI$^+$, *m*/*z*): Calculated for $[M - H_2O + H]^+$ ($C_{64}H_{77}B_2N_8O_{25}^+$): 1379.5180, found 1379.5317.

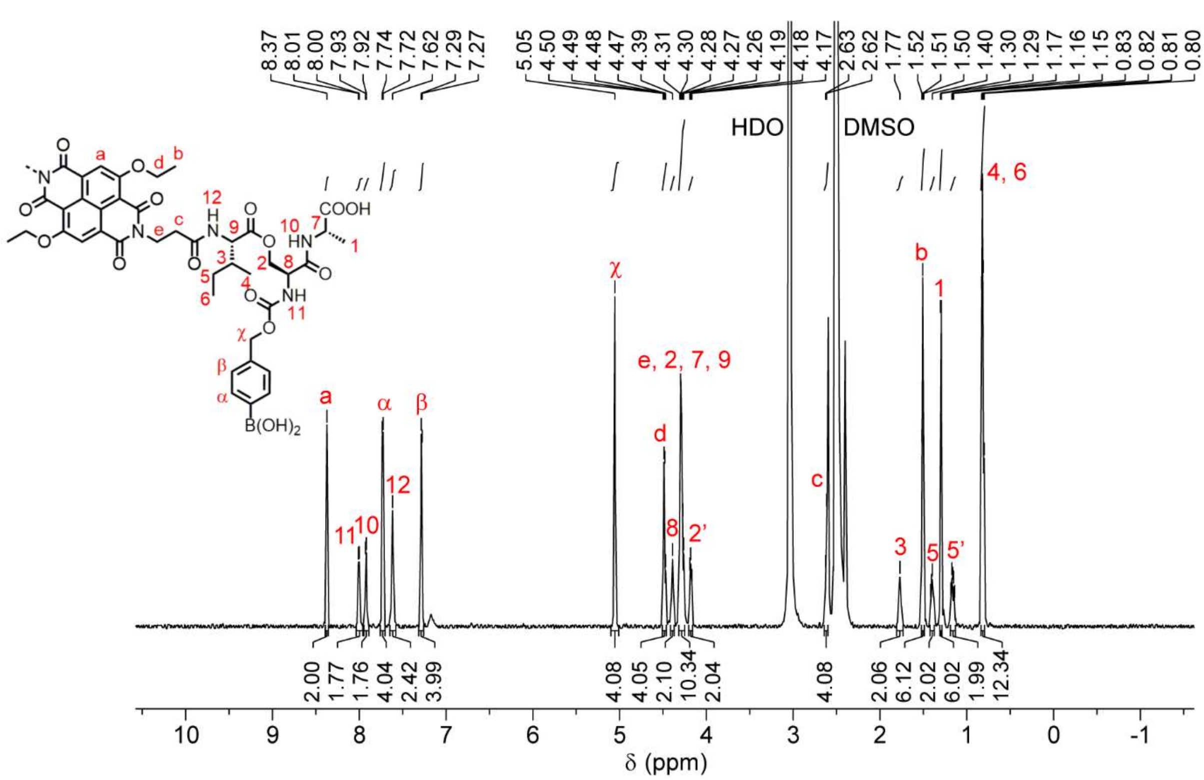

Supplementary Fig. 2| ^{1}H NMR spectrum (700 MHz, DMSO-d_6, 353 K) of compound **1**.

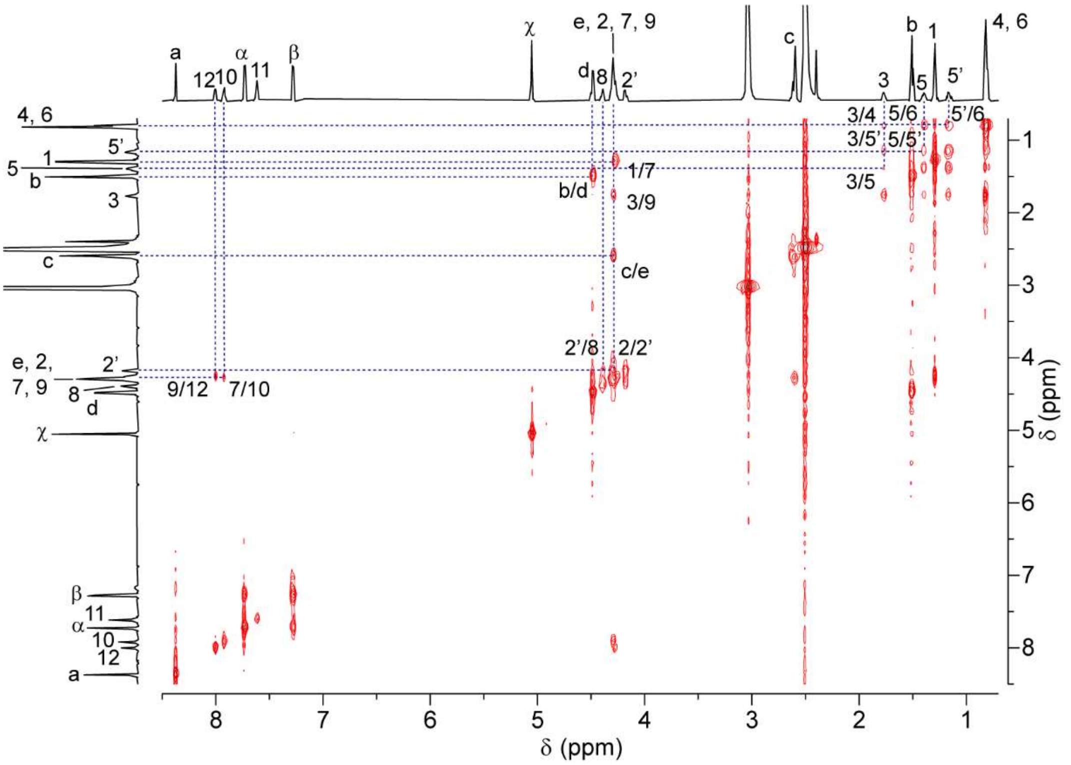

Supplementary Fig. 3| ^{1}H NMR and ^{1}H,^{1}H COSY NMR spectra (700 MHz, DMSO-d_6, 353 K) of compound **1**. The correlations between ^{1}H signals are highlighted with blue dashed lines in the COSY spectra.

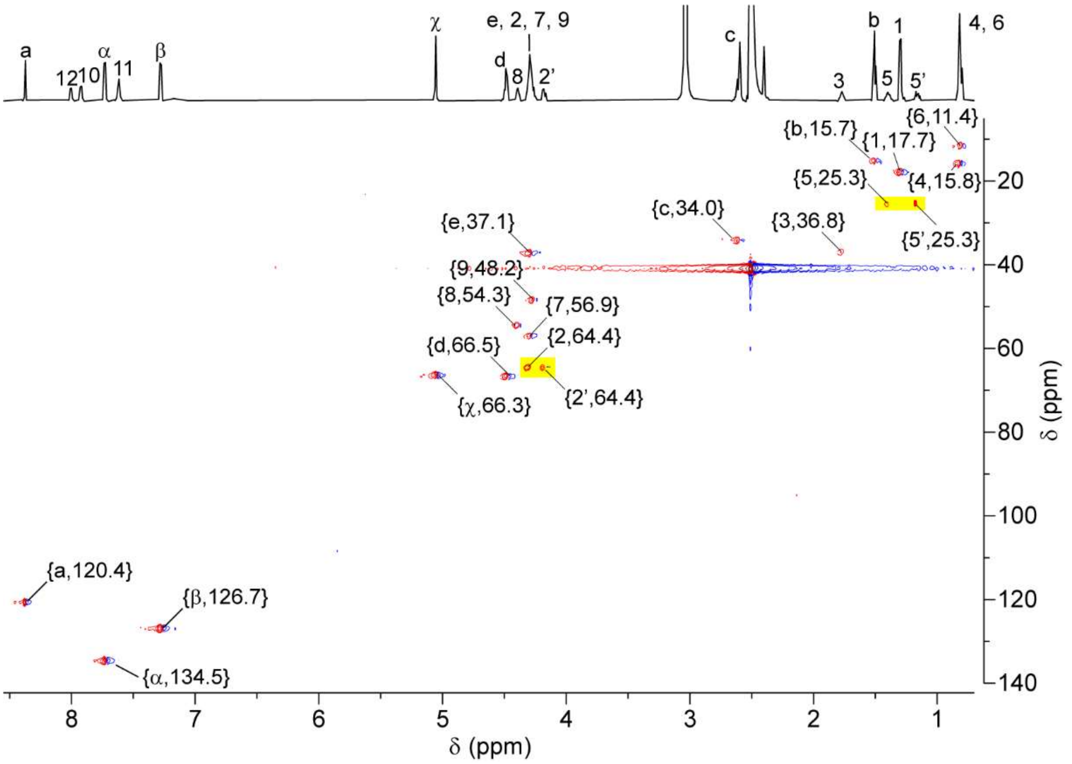

Supplementary Fig. 4| ^{1}H NMR and ^{1}H,^{13}C HSQC NMR spectra (700 MHz, DMSO-d_6, 353 K) of compound **1**. Splitting of H_2 and H_5 traces are highlighted in the HSQC spectra.

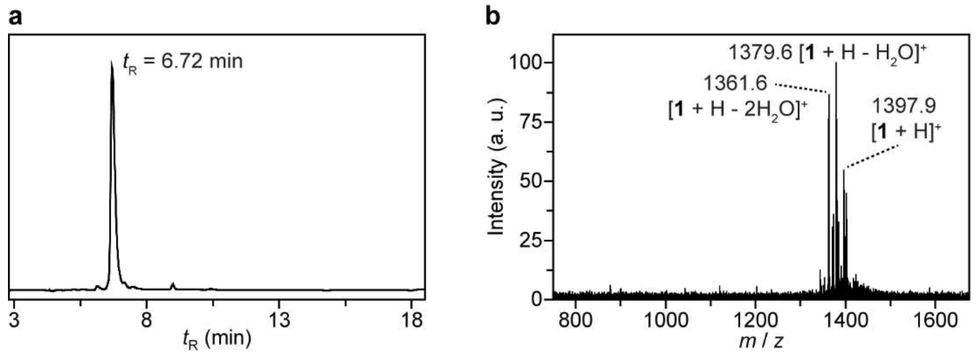

Supplementary Fig. 5| LC-MS study of compound **1**. **a**, HPLC trace of **1.** Retention time (t_R) = 6.72 min. **b**, Convoluted MS spectrum for t_R = 6.15-6.90 min, showing peaks of $[M + H]^+$, $[M + H - 2H_2O]^+$ and $[M + H - H_2O]^+$

2.2 Compound 2

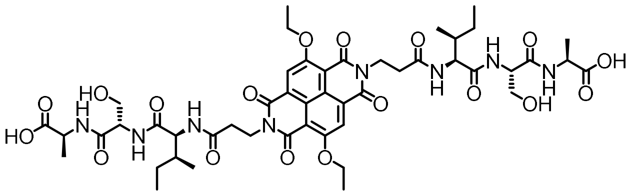

Synthesis: Compound **2** was synthesized using Merrifield's Fmoc solid-phase peptide synthesis strategy, constructing the peptide from the *C*-terminus to the *N*-terminus in a microwave-assisted peptide synthesizer at a 0.25 mmol scale. Fmoc-Ala preloaded Wang resin (0.5 mmol) was swollen in DMF for 1 h before use. After deprotection of the Fmoc group (3 mL of 20% piperidine in DMF, 2 and 5 min at 75 °C) (i), Fmoc-Ser(tBu) (ii), Fmoc-Ile (iii) were double coupled onto the peptide at 75 °C for 20 min. 5 equiv. of the compounds were used in 2.5 mL DMF. PyBOP (5 equiv. in 1 mL DMF) and DIPEA (10 equiv. in 0.5 mL) were used for the coupling reaction. After deprotection of the Fmoc group (3 mL of 20% piperidine in DMF, 2 and 5 min at 75 °C) (i), 0.2 equiv. of 3,3'-(4,9-diethoxy-1,3,6,8-tetraoxo-1,3,6,8-tetrahydrobenzo[lmn][3,8]phenanthroline-2,7-diyl)dipropionic acid were coupled onto the peptide at room temperature overnight (iv). PyBOP (5 equiv. in 1 mL DMF) and DIPEA (10 equiv. in 0.5 mL) were used for the coupling reaction. The product was cleaved from the resin by using 2.5 mL of a cleavage cocktail (95% CF_3COOH, 2.5% TIPS, 2.5% H_2O). The product was purified by HPLC using the Zorbax Eclipse column at a flowrate of 4 mL/min. The gradient started with 20% CH_3CN in H_2O (+0.1% CF_3COOH) and this solvent ratio was kept for 1 min, after which the CH_3CN content was increased to 100% in 15 min. The compound eluted from the column after 5.5 min and was received as a yellow powder after lyophilization (10.3 mg, 3.5% overall yield).

^{1}H NMR [700 MHz, PB (pH = 7.4, 5 mM) in D_2O, 353 K]: δ 9.07 (s, 2H), 5.20 (q, *J* = 6.4 Hz, 4H), 5.18 – 5.12 (m, 2H), 5.02 (t, *J* = 5.6 Hz, 2H), 4.98 – 4.92 (m, 2H), 4.41 (d, *J* = 5.7 Hz, 4H), 3.48 – 3.31 (m, 4H), 2.28 (s, 2H), 2.18 (t, *J* = 6.6 Hz, 6H), 1.90 (d, *J* = 7.3 Hz, 6H), 1.81 (s, 2H), 1.51 (s, 2H), 1.34 (d, *J* = 5.9 Hz, 6H), 1.16 (t, *J* = 6.9 Hz, 6H).

LCMS: LC retention time (min): 5.54. MS (ESI$^+$, *m/z*): 1041.4 $[M + H]^+$, 1063.4 $[M + Na]^+$.

HRMS (ESI$^+$, *m/z*): Calculated for $[M + H]^+$ ($C_{48}H_{64}N_8O_{18}^+$): 1041.4411, found 1041.4440.

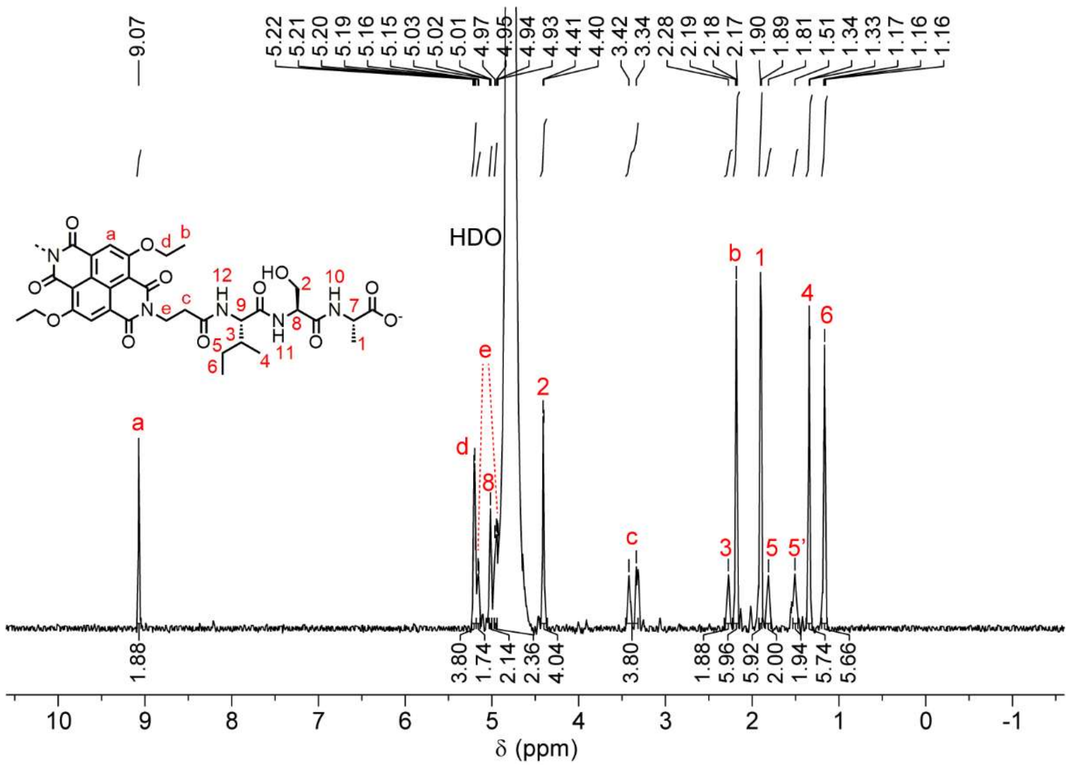

Supplementary Fig. 6| ^{1}H NMR spectrum [700 MHz, PB (pH = 7.4, 5 mM) in D_2O, 353 K] of compound **2**.

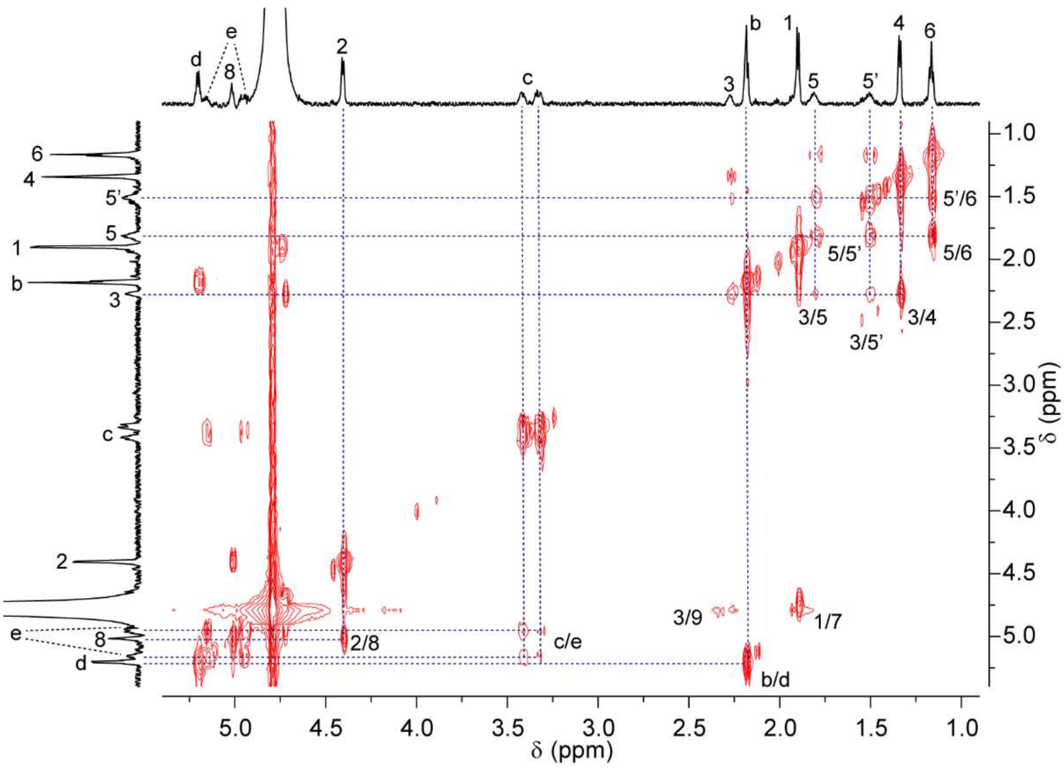

Supplementary Fig. 7| ^{1}H NMR and ^{1}H,^{1}H COSY NMR spectra [700 MHz, PB (pH = 7.4, 5 mM) in D_2O, 353 K] of compound **2**. The correlations between ^{1}H signals are highlighted with blue dashed lines in the COSY spectra.

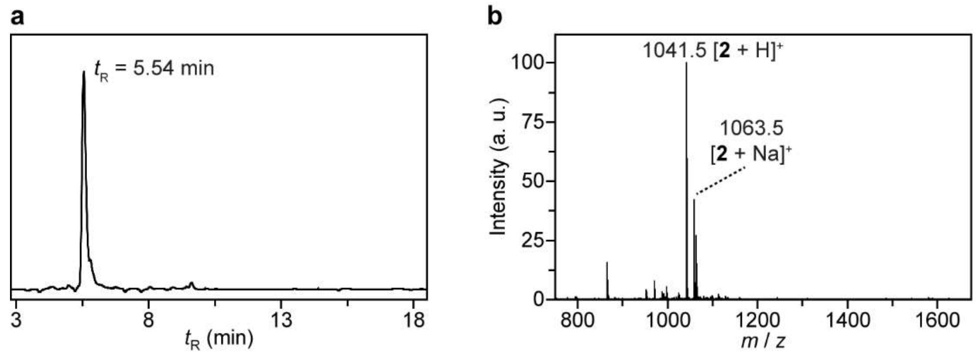

Supplementary Fig. 8| LC-MS study of compound **2**. **a**, HPLC trace of **2.** Retention time (t_R) = 5.54 min. **b**, Convoluted MS spectrum for t_R = 5.26-5.90 min, showing peaks of $[M + H]^+$ and $[M + Na]^+$.

2.3 Compound 3

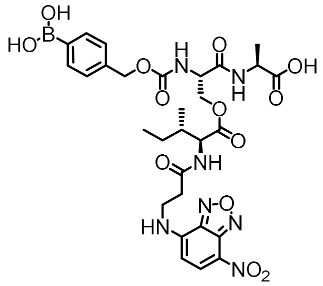

Synthesis: Compound **3** was synthesized using Merrifield's Fmoc solid-phase peptide synthesis strategy, constructing the peptide from the *C*-terminus to the *N*-terminus in a microwave-assisted peptide synthesizer at a 0.25 mmol scale. Fmoc-Ala preloaded Wang resin (0.5 mmol) was swollen in DMF for 1 h before use. First, the Fmoc group was removed by two consecutive deprotection steps (2 and 5 min) with 20% piperidine in DMF (10 mL) at 75 °C (i). After deprotection, the resin was washed four times with DMF (7 mL). Fmoc-serine (5 equiv. in 10 mL DMF) was coupled to the *N*-terminus by using the activator PyBOP (5 equiv. in 4 mL DMF) and activator base DIPEA (10 equiv. in 2 mL DMF) at 75 °C for 20 min (ii). After deprotection of the Fmoc protecting group (i), the *N* terminus was modified using 4-nitrophenyl (4-(4,4,5,5-tetramethyl-1,3,2-dioxaborolan-2-yl)benzyl) carbonate (1.25 equiv.) in 10 mL DMF and DIPEA (5 equiv.) overnight at room temperature (iii). After the resin was washed with DMF and DCM, Fmoc-Ile (10 equiv. in 5 mL DMF), DIC (10 equiv.) and 4-DMAP (1 equiv.) were added to form the ester bond in the serine side chain. After the reaction mixture was stirred for 12 h at room temperature, the resin was washed with DMF and DCM, dried (iv). The Fmoc group was then removed in two deprotection steps using 20% piperidine in DMF (10 min each, 5 mL) at room temperature (v). 3-((7-nitrobenzo[c][1,2,5]oxadiazol-4-yl)amino)propanoic acid (0.2 equiv.) was coupled onto the *N*-terminus overnight at room temperature, using HATU (5 equiv.) and DIPEA (5 equiv.) as the coupling reagents. The resin was washed with DMF and DCM, dried (vi). The product was cleaved from the resin by using 2.5 mL of a cleavage cocktail (95% CF_3COOH, 2.5% TIPS, 2.5% H_2O). This step also removed the pinacol protecting group of the PBA. After 2 hours, the cleavage cocktail was removed in vacuo (vii). The product was purified by HPLC using the Zorbax Eclipse column at a flowrate of 4 mL/min. The gradient started with 20% CH_3CN in H_2O (+0.1% CF_3COOH) and this solvent ratio was kept for 1 min, after which the CH_3CN content was increased

to 100% in 15 min. The product **3** eluted from the column after 11.8 min and was received as an orange powder after lyophilization (8 mg, 11.3% overall yield).

^{1}H NMR (700 MHz, DMSO-d_6, 293 K): δ 9.23 (s, 1H), 8.51 (d, *J* = 8.9 Hz, 1H), 8.15 (d, *J* = 6.3 Hz, 1H), 8.05 (d, *J* = 9.3 Hz, 1H), 7.77 (d, *J* = 8.1 Hz, 2H), 7.55 – 7.36 (m, 1H), 7.31 (d, *J* = 7.8 Hz, 2H), 6.45 (d, *J* = 9.0 Hz, 1H), 5.06 (s, 2H), 4.57 – 4.37 (m, 2H), 4.34 – 4.17 (m, 3H), 3.88 – 3.65 (m, 2H), 2.84 – 2.58 (m, 2H), 1.91 – 1.69 (m, 1H), 1.45 – 1.20 (m, 4H), 1.18 – 0.96 (m, 1H), 0.85 – 0.73 (m, 6H).

LCMS: LC retention time (min): 5.94. MS (ESI$^+$, *m*/*z*): 702.3 [M + H]$^+$, 724.3 [M + Na]$^+$.

HRMS (ESI$^+$, *m*/*z*): Calculated for [M + Na]$^+$ ($C_{29}H_{36}BN_7NaO_{13}^+$): 724.2362, found 724.2328.

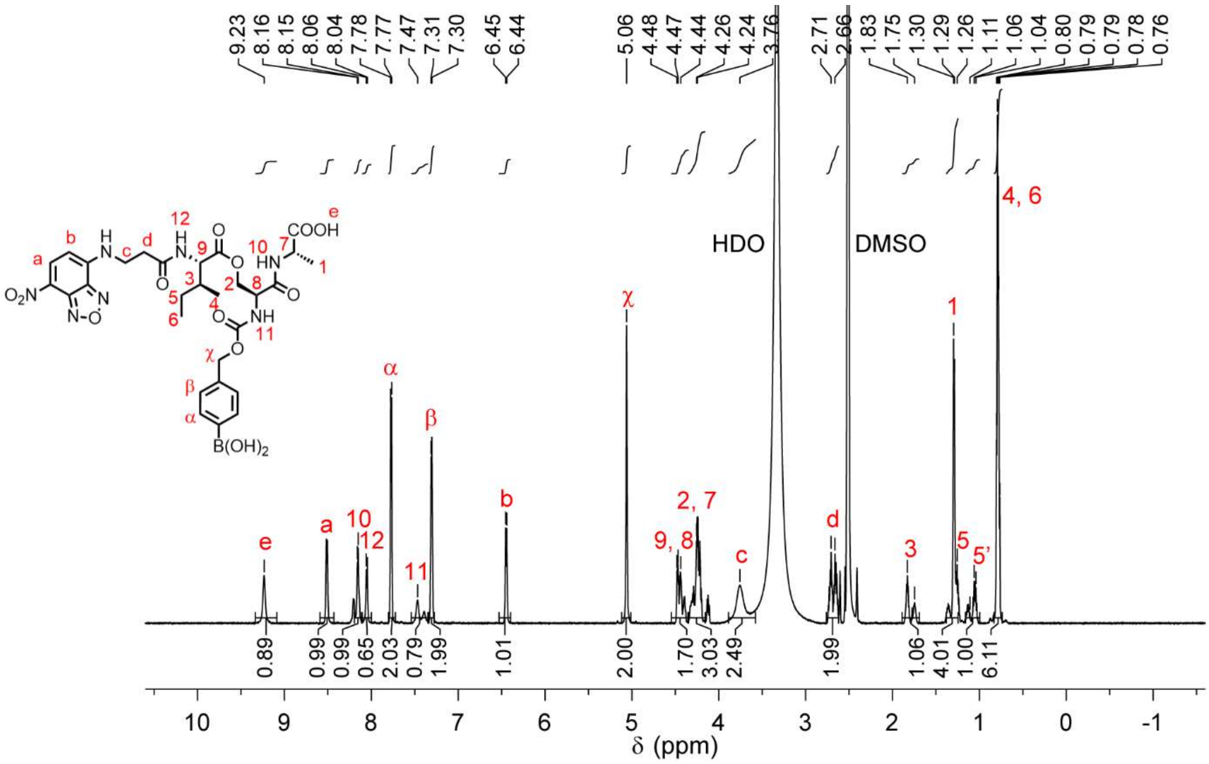

Supplementary Fig. 9| ^{1}H NMR spectrum (700 MHz, DMSO-d_6, 293 K) of compound **3**.

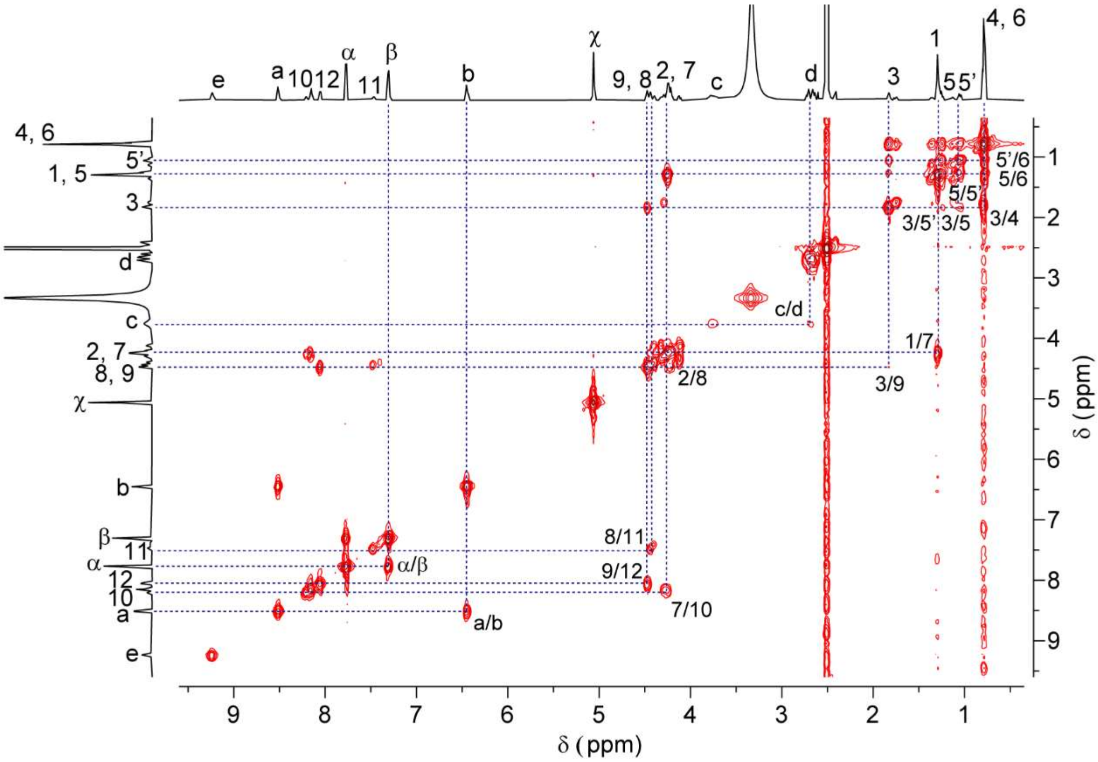

Supplementary Fig. 10| ^{1}H NMR and ^{1}H,^{1}H COSY NMR spectra (700 MHz, DMSO-d_6, 293 K) of compound **3**. The correlations between ^{1}H signals are highlighted with blue dashed lines in the COSY spectra.

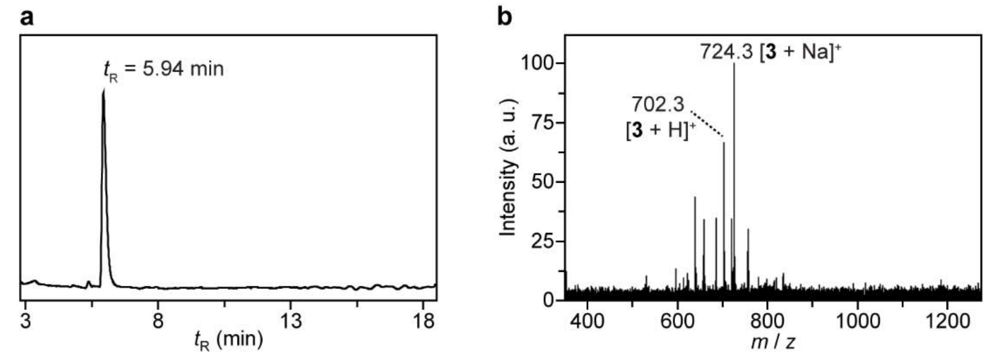

Supplementary Fig. 11| LC-MS study of compound **3**. **a**, HPLC trace of **3.** Retention time (t_R) = 5.94 min. **b**, Convoluted MS spectrum for t_R = 5.90-6.15 min, showing peaks of [M + H]$^+$ and [M + Na]$^+$

2.4 Compound 4

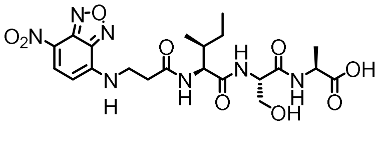

Synthesis: Compound **4** was synthesized using the Fmoc solid phase peptide synthesis strategy by Merrifield, synthesizing the peptide from *C* to *N*-terminus in a microwave assisted peptide synthesizer at a 0.25 mmol scale. Fmoc-Ala preloaded Wang resin (0.25 mmol) was swollen in DMF for 1 h before

use. After deprotection of the Fmoc group (3 mL of 20% piperidine in DMF, 2 and 5 min at 75 °C) (i), Fmoc-Ser(tBu) (ii), Fmoc-Ile (iii) were double coupled onto the peptide at 75 °C for 20 min. 5 equiv. of the compounds were used in 2.5 mL DMF. PyBOP (5 equiv. in 1 mL DMF) and DIPEA (10 equiv. in 0.5 mL) were used for the coupling reaction. After deprotection of the Fmoc group (3 mL of 20% piperidine in DMF, 2 and 5 min at 75 °C) (i), 0.2 equiv. of 3-((7-nitrobenzo[c][1,2,5]oxadiazol-4-yl)amino)propanoic acid were coupled onto the peptide at room temperature overnight (iv). PyBOP (5 equiv. in 1 mL DMF) and DIPEA (10 equiv. in 0.5 mL) were used for the coupling reaction. The product was cleaved from the resin by using 2.5 mL of a cleavage cocktail (95% CF_3COOH, 2.5% TIPS, 2.5% H_2O). The product was purified by HPLC using the Zorbax Eclipse column at a flowrate of 4 mL/min. The gradient started with 20% CH_3CN in H_2O (+0.1% CF_3COOH) and this solvent ratio was kept for 1 min, after which the CH_3CN content was increased to 100% in 15 min. The compound eluted from the column after 5.5 min and was received as an orange powder after lyophilization (8.3 mg, 31.7% overall yield).

^{1}H NMR (700 MHz, DMSO-*d*$_6$, 293 K): δ 9.42 (s, 1H), 8.54 (d, *J* = 8.2 Hz, 1H), 8.12 (d, *J* = 7.5 Hz, 1H), 8.01 (d, *J* = 8.0 Hz, 1H), 7.96 (d, *J* = 6.9 Hz, 1H), 6.46 (d, *J* = 9.1 Hz, 1H), 4.30 (q, *J* = 6.6 Hz, 1H), 4.24 (t, *J* = 7.9 Hz, 1H), 4.18 (p, *J* = 7.3 Hz, 1H), 3.72 – 3.67 (m, 2H), 3.62 – 3.53 (m, 2H), 2.69 – 2.57 (m, 2H), 1.70 – 1.64 (m, 1H), 1.42 – 1.32 (m, 1H), 1.25 (d, *J* = 7.2 Hz, 3H), 1.05 – 0.98 (m, 1H), 0.80 (d, *J* = 6.8 Hz, 3H), 0.74 – 0.69 (m, 3H). [700 MHz, PB (pH = 7.4, 5 mM) in D_2O, 353 K]: δ 8.58 (d, *J* = 9.1 Hz, 1H), 6.44 (d, *J* = 9.2 Hz, 1H), 4.34 (t, *J* = 5.8 Hz, 1H), 4.12 – 4.06 (m, 2H), 3.98 – 3.83 (m, 2H), 3.78 (d, *J* = 5.6 Hz, 2H), 2.84 – 2.69 (m, 2H), 1.72 – 1.66 (m, 1H), 1.29 (d, *J* = 7.2 Hz, 3H), 1.27 – 1.20 (m, 1H), 1.00 – 0.89 (m, 1H), 0.77 (d, *J* = 6.8 Hz, 3H), 0.66 (t, *J* = 7.5 Hz, 3H).

LCMS: LC retention time (min): 4.78. MS (ESI$^+$, *m*/*z*): 524.2 $[M + H]^+$, 1047.5 $[2M + H]^+$.

HRMS (ESI$^+$, *m*/*z*): Calculated for $[M + H]^+$ ($C_{21}H_{30}N_7O_9^+$): 524.2105, found 524.2106.

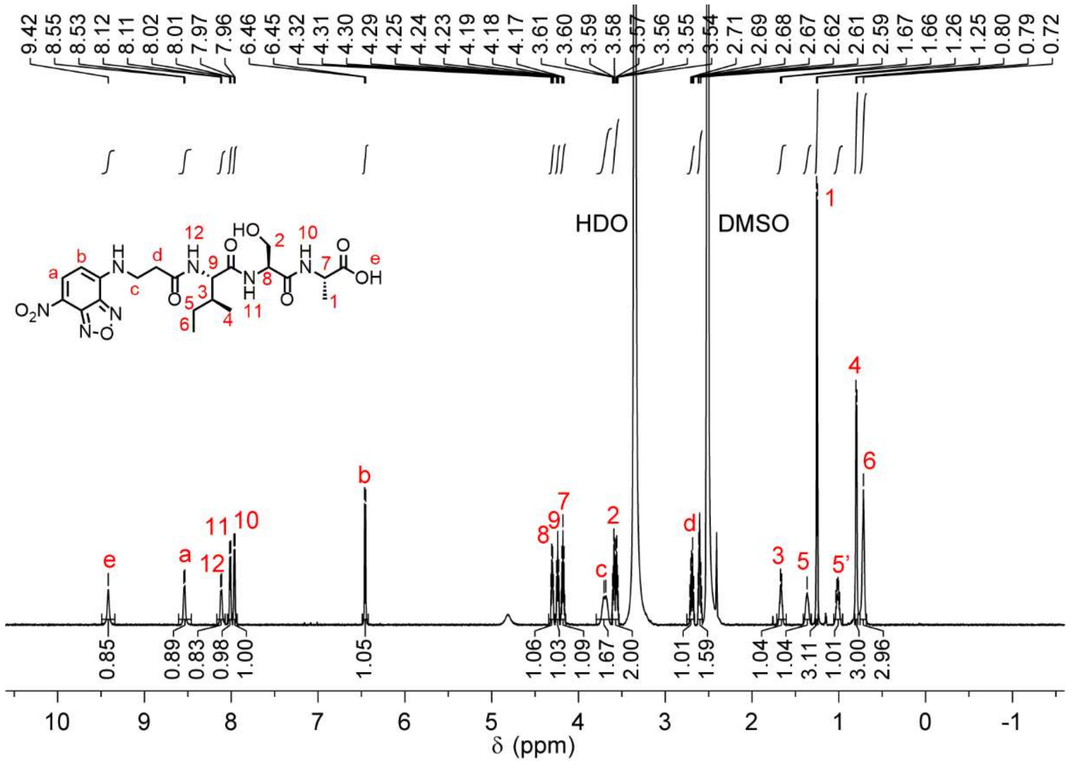

Supplementary Fig. 12| ^{1}H NMR spectrum (700 MHz, DMSO-d_6, 293 K) of compound **4**.

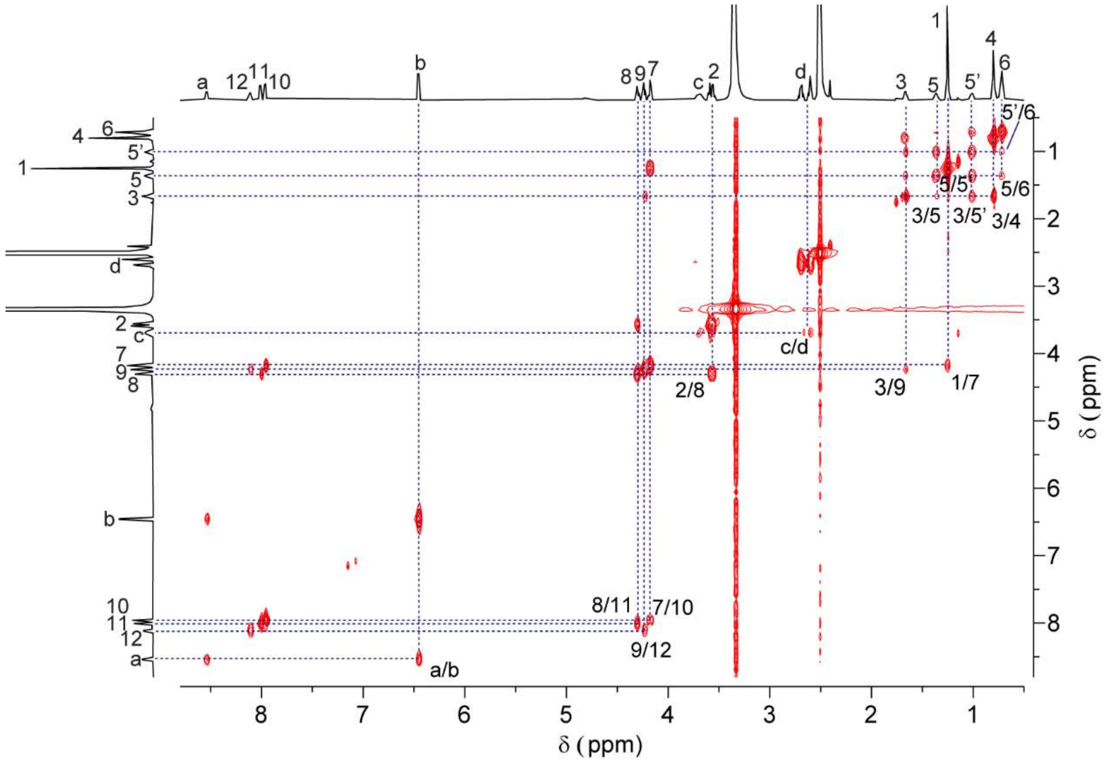

Supplementary Fig. 13| ^{1}H NMR and ^{1}H,^{1}H COSY NMR spectra (700 MHz, DMSO-d_6, 293 K) of compound **4**. The correlations between ^{1}H signals are highlighted with blue dashed lines in the COSY spectra.

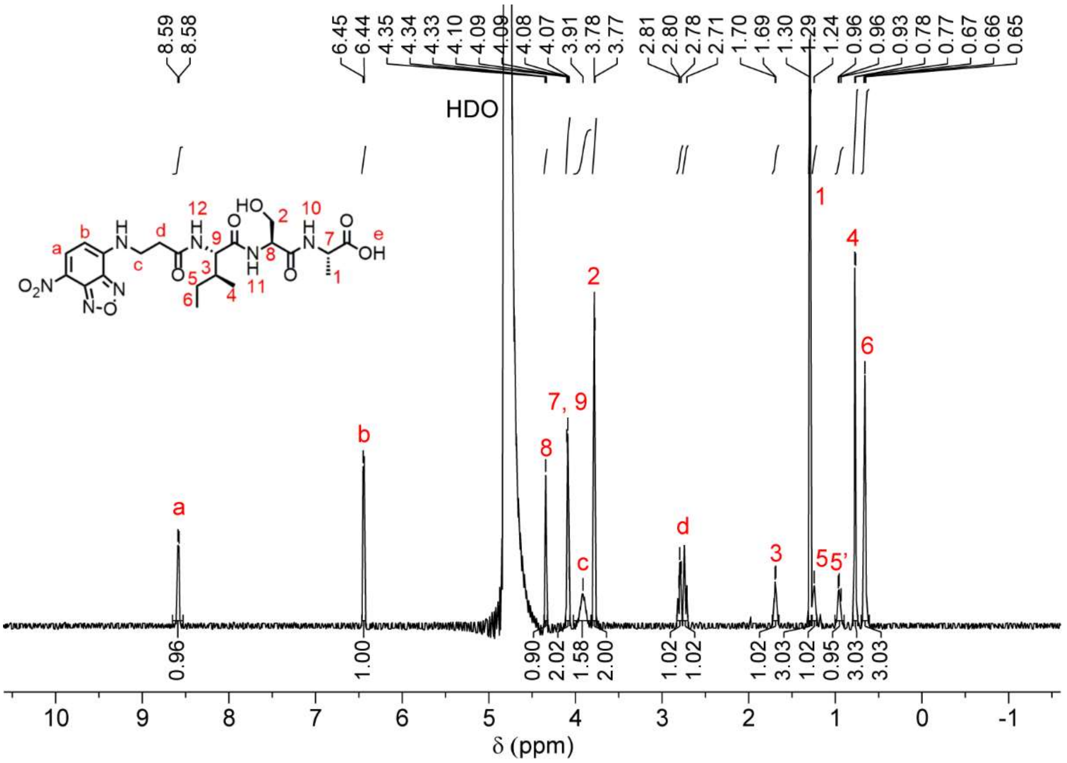

Supplementary Fig. 14| ^{1}H NMR spectrum [700 MHz, PB (pH = 7.4, 50 mM) in D_2O, 293 K] of compound **4** (500 μM). Well-defined signals with high intensity were obtained, suggesting no aggregation of compound **4** under this condition.

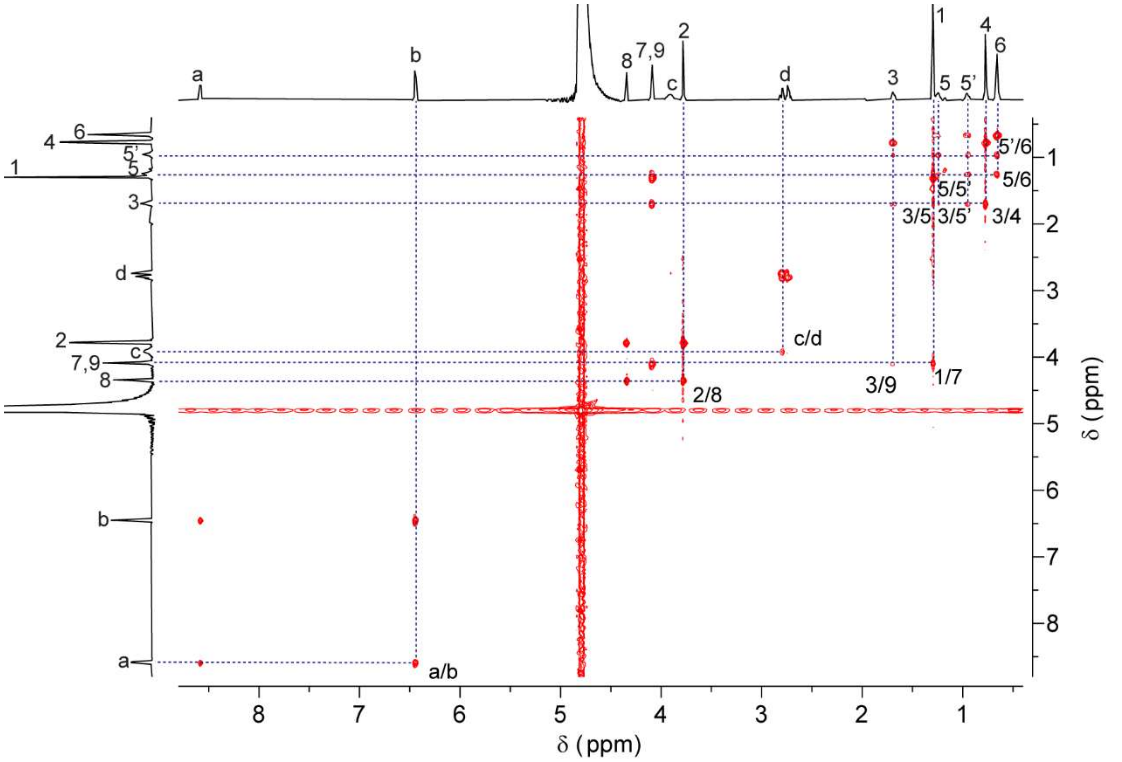

Supplementary Fig. 15| ^{1}H NMR and $^{1}H,^{1}H$ COSY NMR spectra [700 MHz, PB (pH = 7.4, 50 mM) in D_2O, 293 K] of compound **4** (500 μM). The correlations between ^{1}H signals are highlighted with blue dashed lines in the COSY spectra. Well-defined signals with high intensity were obtained, suggesting no aggregation of compound **4** under this condition.

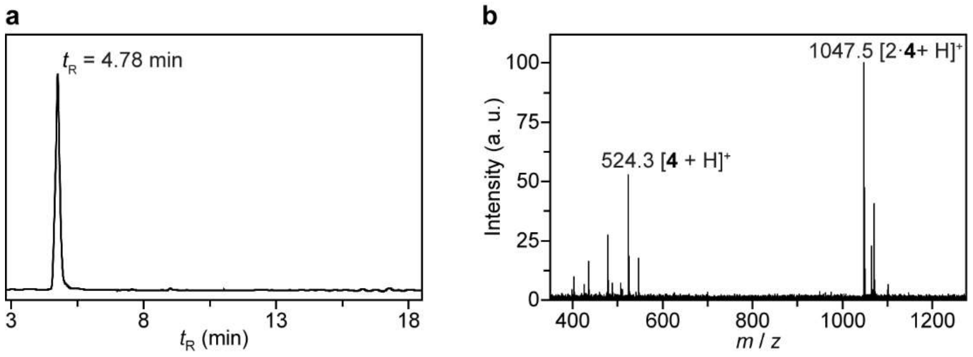

Supplementary Fig. 16| LC-MS study of compound **4**. **a**, HPLC trace of **4.** Retention time (t_R) = 4.78 min. **b**, Convoluted MS spectrum for t_R = 4.75-4.95 min, showing peaks of [M + H]$^+$ and [2M + H]$^+$

2.5 Compound 1·TAT/3TAT

Compounds **1·TAT**/ **3·TAT** were obtained by mixing compound **1/3** and SHA-TAT in DPBS solution at a 1:2 volume ratio for **1·TAT** and a 1:1 volume ratio for **3·TAT**, respectively. The resulting compounds were used directly in subsequent experiments without further purification.

To confirm the formation of the TAT-functionalized compounds by PBA-SHA dynamic covalent interaction, an Alizarin Red S assay was conducted in addition to the MALDI-TOF-MS analysis. A mixture containing phenylboronic acid compounds (50 μM for **1** and 100 μM for **3**, respectively), TAT-SHA (0 to 140 μM, 20 μM per increment), and Alizarin Red S (250 μM) was prepared in DPBS and incubated in an orbital shaker for 30 minutes. The fluorescence of the mixture (λ_{ex} = 495 nm, λ_{em} = 600 nm) was recorded and plotted as a function of the stoichiometric ratio between TAT-SHA and the phenylboronic acid-containing compound. The endpoint of the titration is indicated when no further changes in emission are observed upon the addition of more **SHA-TAT**.

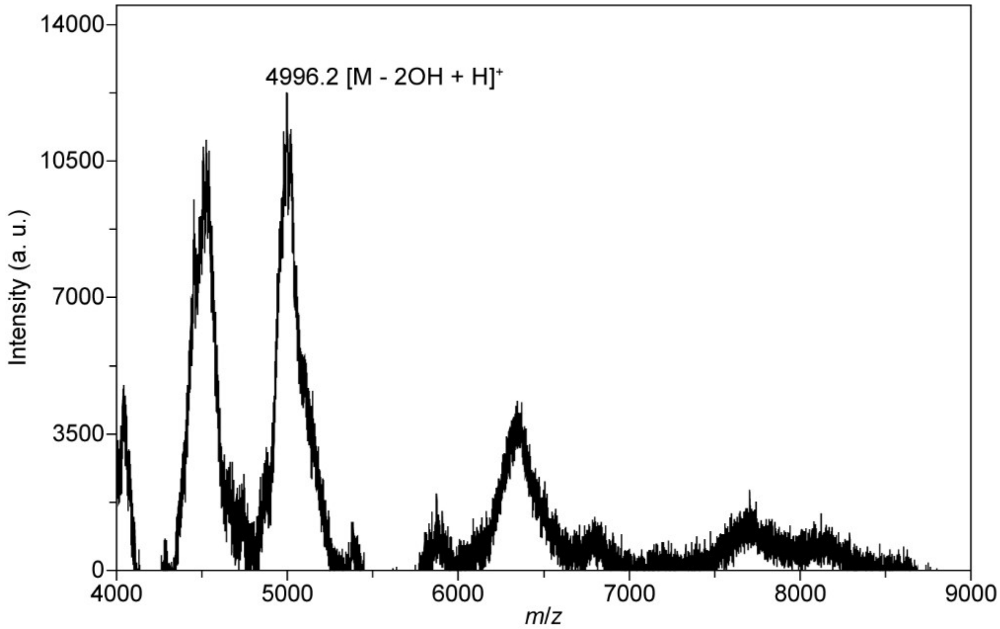

Supplementary Fig. 17| MALDI-TOF-MS spectrum for 1·TAT. Peak at *m*/*z* = 4996.2 can be identified, correspond to [M – 2OH + H]$^+$.

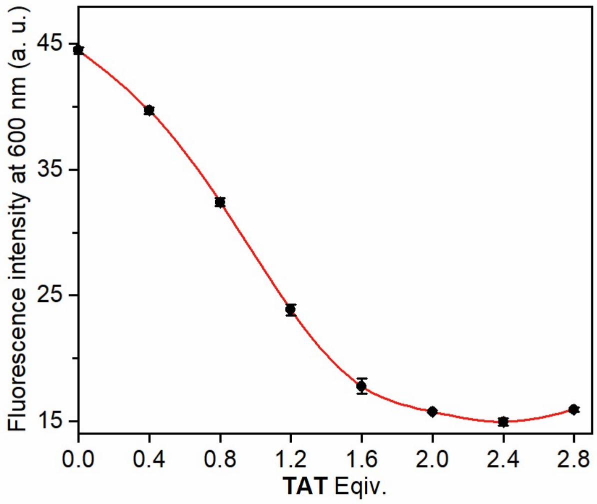

Supplementary Fig. 18| Fluorescence titration curves displaying the stoichiometric substitution of the fluorogenic Alizarin Red S/boronic acid complex on compound **1** by **SHA-TAT**. The endpoint of the titration (2.0 eqiv.) corresponds to the results obtained through MALDI-TOF-MS analysis (Supplementary Fig. 17).

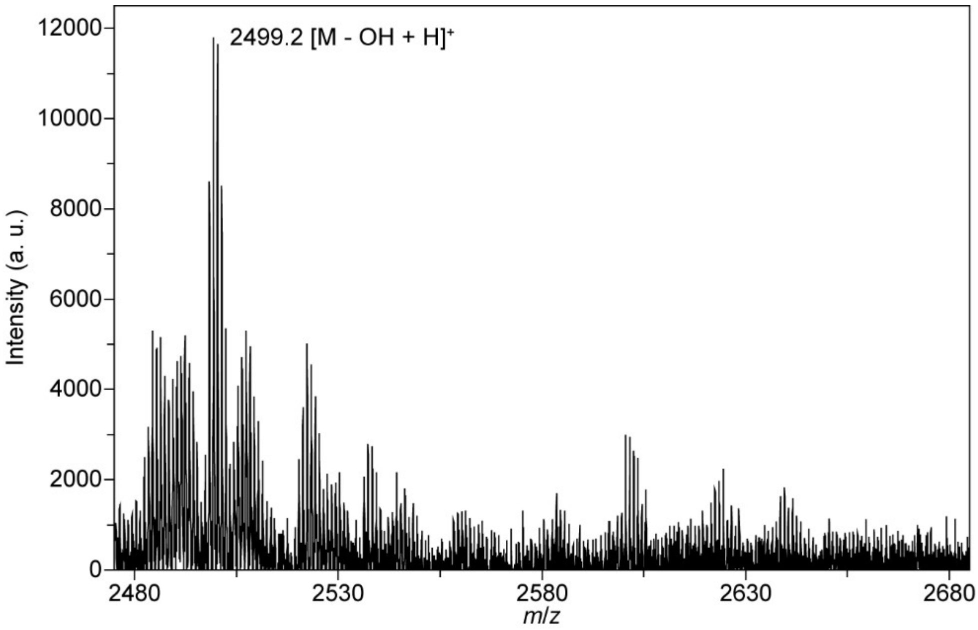

Supplementary Fig. 19| MALDI-TOF-MS spectrum for 3·TAT. Peak at m/z = 2499.2 can be identified, correspond to $[M – OH + H]^+$.

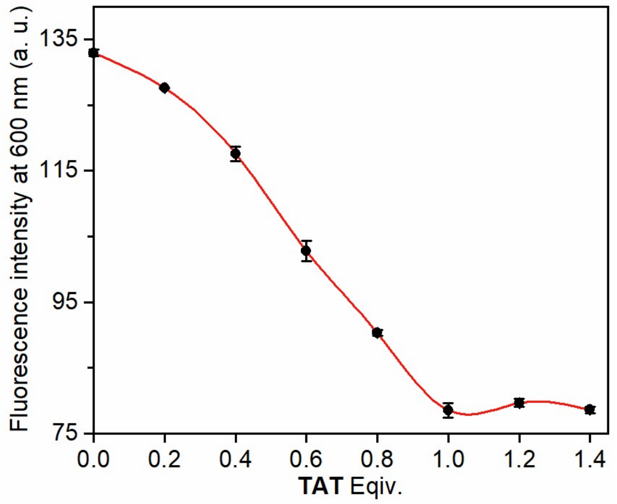

Supplementary Fig. 20| Fluorescence titration curves displaying the stoichiometric substitution of the fluorogenic Alizarin Red S/boronic acid complex on compound **3** by **SHA-TAT**. The endpoint of the titration (1.0 eqiv.) corresponds to the results obtained through MALDI-TOF-MS analysis (Supplementary Fig. 19).

3 H_2O_2-induced formation of 2

3.1 HPLC-MS analysis of H_2O_2-induced conversion from 1/1·TAT to 2.

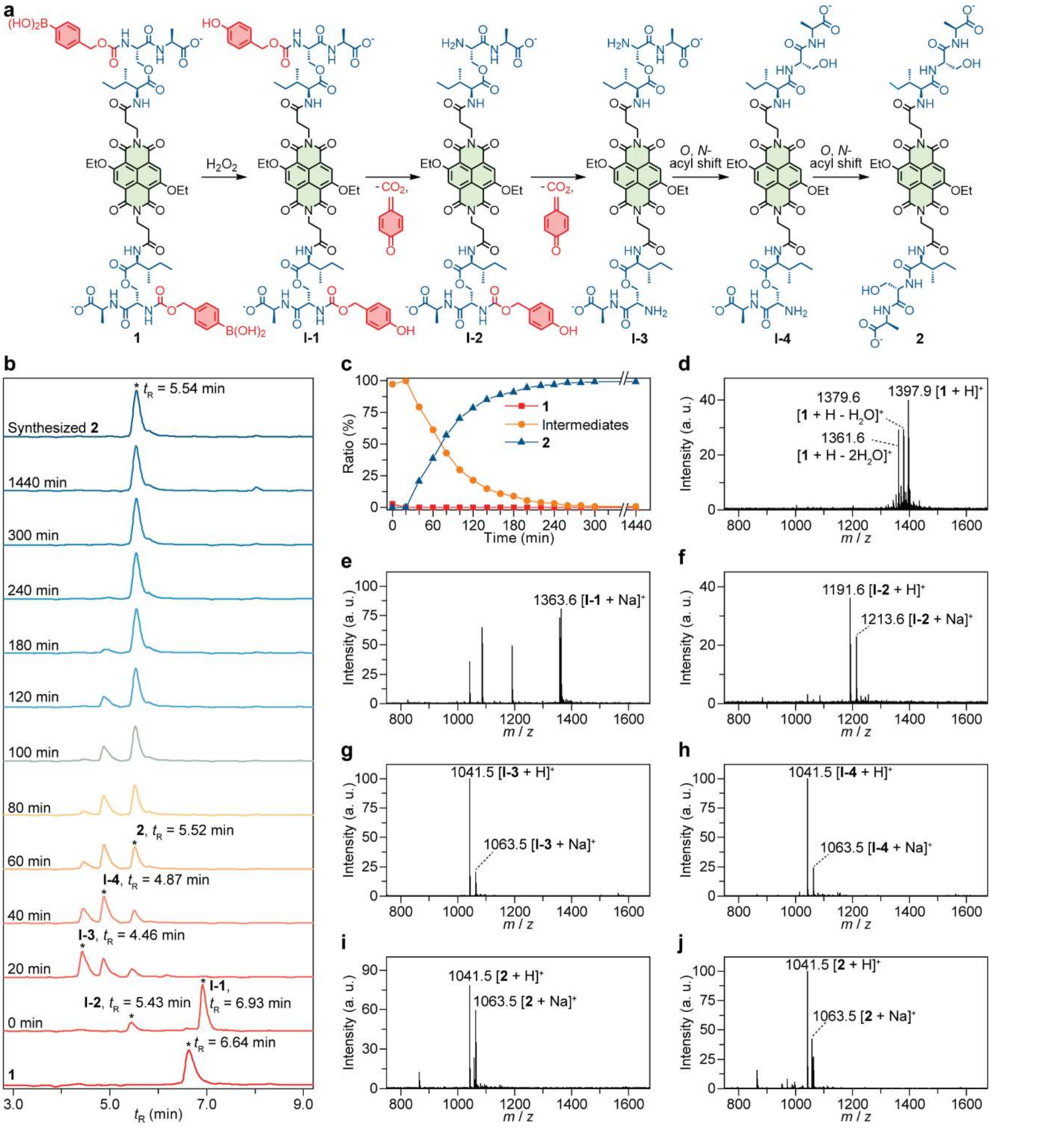

Supplementary Fig. 21| H_2O_2-induced conversion of 1 to 2. a, Reaction scheme for the conversion. The oxidation of the phenylboronic acid by H_2O_2 leads to the formation of intermediate **I-1**, followed by the self-immolation of the carbamate linker to generate **I-2** and **I-3**. The isopeptide bond then undergo *O*, *N*-acyl shift to afford **I-4** and finally compound **2**. **b**, HPLC spectra for H_2O_2 (1.0 mM) induced-conversion of **1** (50 μM) to **2** in a mixture of NH_4HCO_3 buffer (pH 7.4, 10 mM) and THF (*v*:*v* = 1:1). **c**, Molar ratio of precursor **1**, deboronation and rearrangement intermediates, and final product **2** after incubating with H_2O_2 based on the peak integration at 254 nm. Deboronation reaction was completed within 20 min of incubation, followed by *O*, *N*-acyl shift that afford **2** in >95% conversion rate within 240 min of incubation. **d**–**j**, Convoluted MS spectra for compound **1** (t_R = 6.64 min) before the addition of H_2O_2 (**d**), a reaction time of 0 min and t_R = 6.93 min (**e**, identified as compound

I-1), a reaction time of 0 min and t_R = 5.43 min (**f**, identified as compound **I-2**), a reaction time of 20 min and t_R = 4.46 min (**g**, identified as compound **I-3**), a reaction time of 40 min and t_R = 4.87 min (**h**, identified as compound **I-4**), a reaction time of 60 min and t_R = 5.52 min (**i**, identified as compound **2**), and compound **2** obtained by synthesis (t_R = 5.54 min) (**j**). The key information of this figure is presented in the main manuscript as Figs. 2b-d.

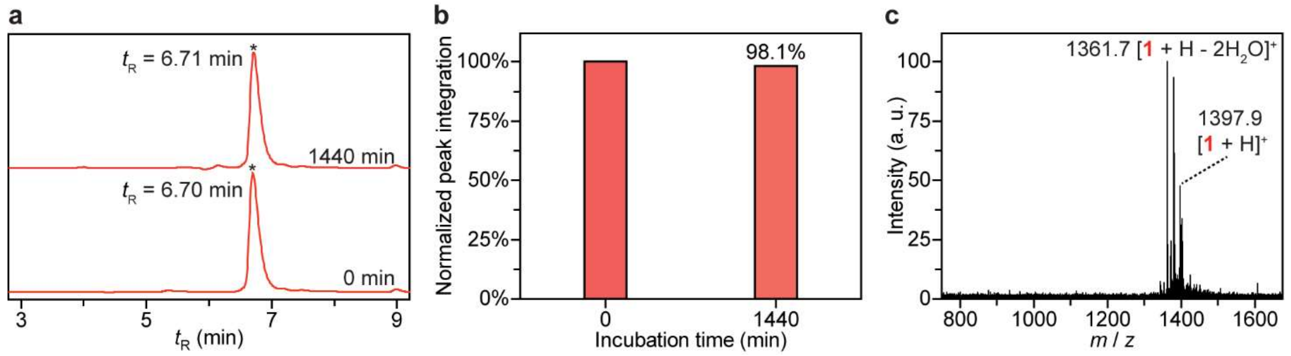

Supplementary Fig. 22| Stability of compound 1. a, Comparison of HPLC spectra of compound **1** (50 μM) incubated in a mixture of NH_4HCO_3 buffer (pH 7.4, 20 mM) and tetrahydrofuran (*v*:*v* = 1:1) for 0 and 1440 min. **b**, Normalized peak integration of the HPLC traces of compound **1** before and after the incubation for 1440 min, which revealed that 98.1% of the compound **1** remained in the mixture. **c**, Convoluted MS spectrum of t_R = 6.71 min after incubation for 1440 min. The peak was identified as compound **1**.

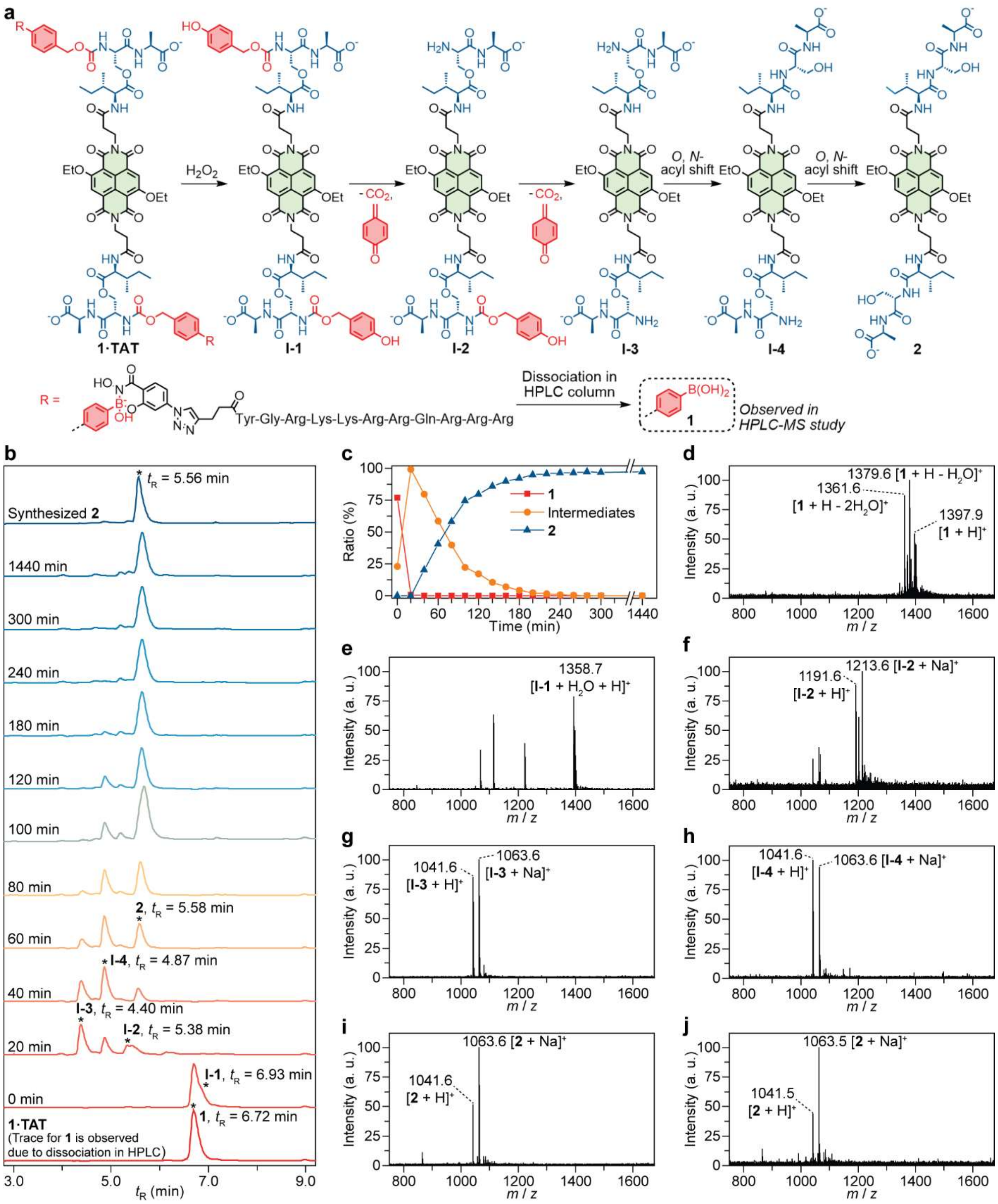

Supplementary Fig. 23| H_2O_2-induced conversion of 1·TAT to 2. a, Reaction scheme for the conversion. The oxidation of the phenylboronic acid by H_2O_2 leads to the formation of intermediate **I-1**, followed by the self-immolation of the carbamate linker to generate **I-2** and **I-3**. The isopeptide bond then undergo *O*, *N*-acyl shift to afford **I-4** and finally compound **2**. **b**, HPLC spectra for H_2O_2 (1.0 mM) induced-conversion of **1·TAT** (50 μM) to **2** in a mixture of NH_4HCO_3 buffer (pH 7.4, 20 mM) and THF (*v*:*v* = 1:1). **c**, Molar ratio of precursor **1**, deboronation and rearrangement intermediates, and **2** after incubating with H_2O_2 based on the peak integration at 254 nm. Deboronation reaction was completed within 20 min of incubation, followed by *O*, *N*-acyl shift that afford **2** in >95% conversion rate within 240 min of incubation**. d–j**, Convoluted MS spectra in the HPLC-MS analysis for compound **1** (t_R = 6.72 min) before addition treatment of H_2O_2 (**d**), a reaction time of 0 min and t_R =

6.93 min (**e**, identified as compound **I-1**), a reaction time of 20 min and t_R = 5.38 min (**f**, identified as compound **I-2**), a reaction time of 20 min and t_R = 4.40 min (**g**, identified as compound **I-3**), a reaction time of 40 min and t_R = 4.87 min (**h**, identified as compound **I-4**), a reaction time of 60 min and t_R = 5.58 min (**i**), identified as compound **2**), and compound **2** obtained by synthesis (t_R = 5.56 min) (**j**). As the boronic acid-SHA dynamic covalent interaction dissociates in the acidic mobile phase of the HPLC, only trace correspond to **1** can be observed after the injection of **1·TAT** to the HPLC.

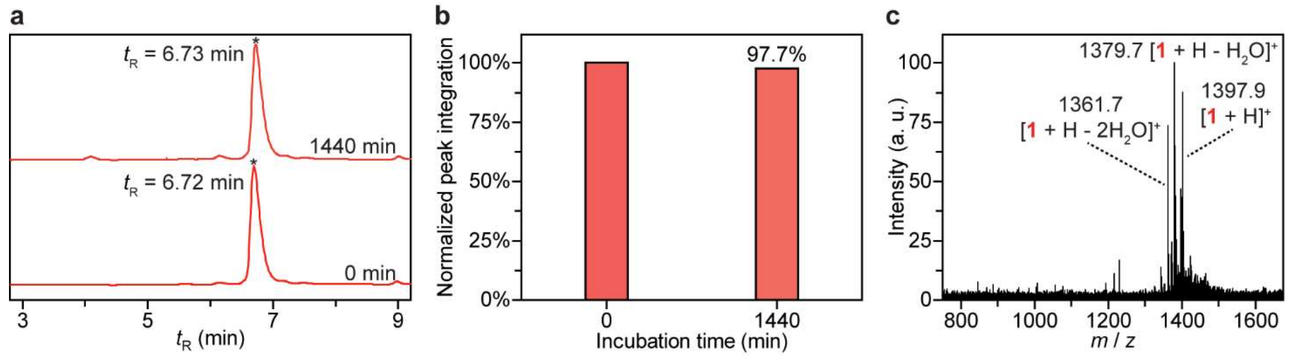

Supplementary Fig. 24| Stability of compound 1·TAT. a, Comparison of HPLC spectra of compound **1·TAT** (50 μM) incubated in a mixture of NH_4HCO_3 buffer (pH 7.4, 20 mM) and tetrahydrofuran (*v*:*v* = 1:1) for 0 and 1440 min. **b**, Normalized peak integration of the HPLC traces of compound **1** before and after the incubation for 1440 min, , which revealed that 97.7% of the compound **1·TAT** remained in the mixture. **c**, Convoluted MS spectrum of t_R = 6.73 min after incubation for 1440 min. The peak was identified as compound **1.** As the boronic acid-SHA dynamic covalent interaction dissociates in the acidic mobile phase of the HPLC, only trace correspond to **1** can be observed after the injection of **1·TAT** to the HPLC.

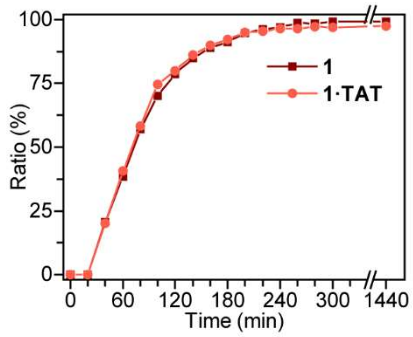

Supplementary Fig. 25| Comparison of conversion rates of final compound **2** generated by H_2O_2-induced conversion of **1** and **1·TAT** based on the peak integration at 254 nm in the corresponding HPLC spectra.

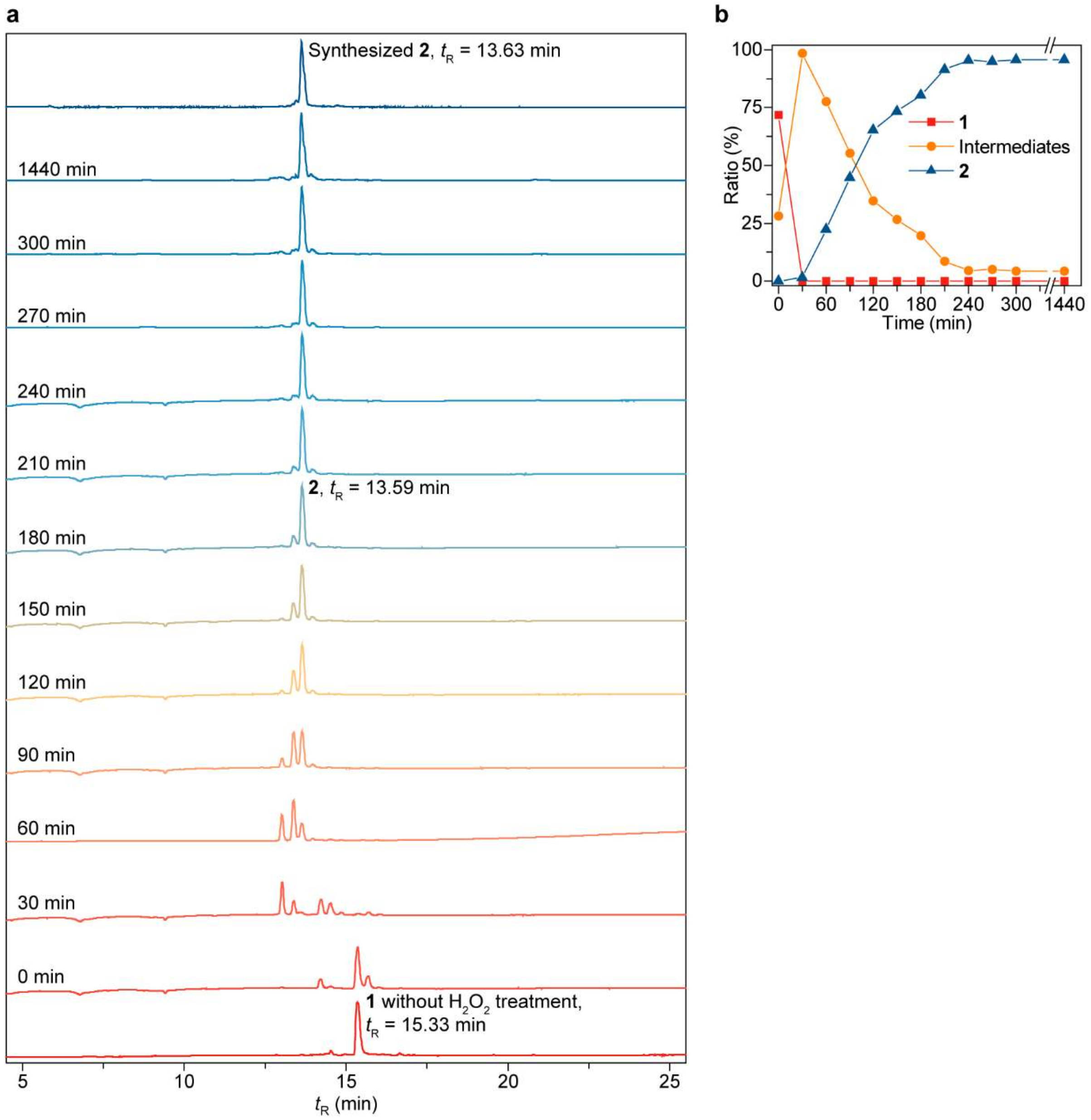

Supplementary Fig. 26| HPLC kinetics analysis of conversion from 1 to 2 at intracellular H_2O_2 concentration. **a**, HPLC spectra for H_2O_2 (2.0 μM) induced-conversion of **1** (1.8 μM) to **2** in a mixture of NH_4HCO_3 buffer (pH 7.4, 20 mM) and tetrahydrofuran (*v*:*v* = 1:1). **b**, Molar ratio of precursor **1**, deboronation and rearrangement intermediates intermediates, and final linearized compound **2** after incubating with H_2O_2 based on the peak integration at 254 nm. Deboronation reaction was completed within 20 min of incubation, followed by *O*, *N*-acyl shift that afford **2** in >95% conversion rate within 240 min of incubation, in agreement with the HPLC-MS analysis on conversion of **1** at higher concentrations.

3.2 HPLC-MS analysis of H_2O_2-induced conversion from 3/3·TAT to 4.

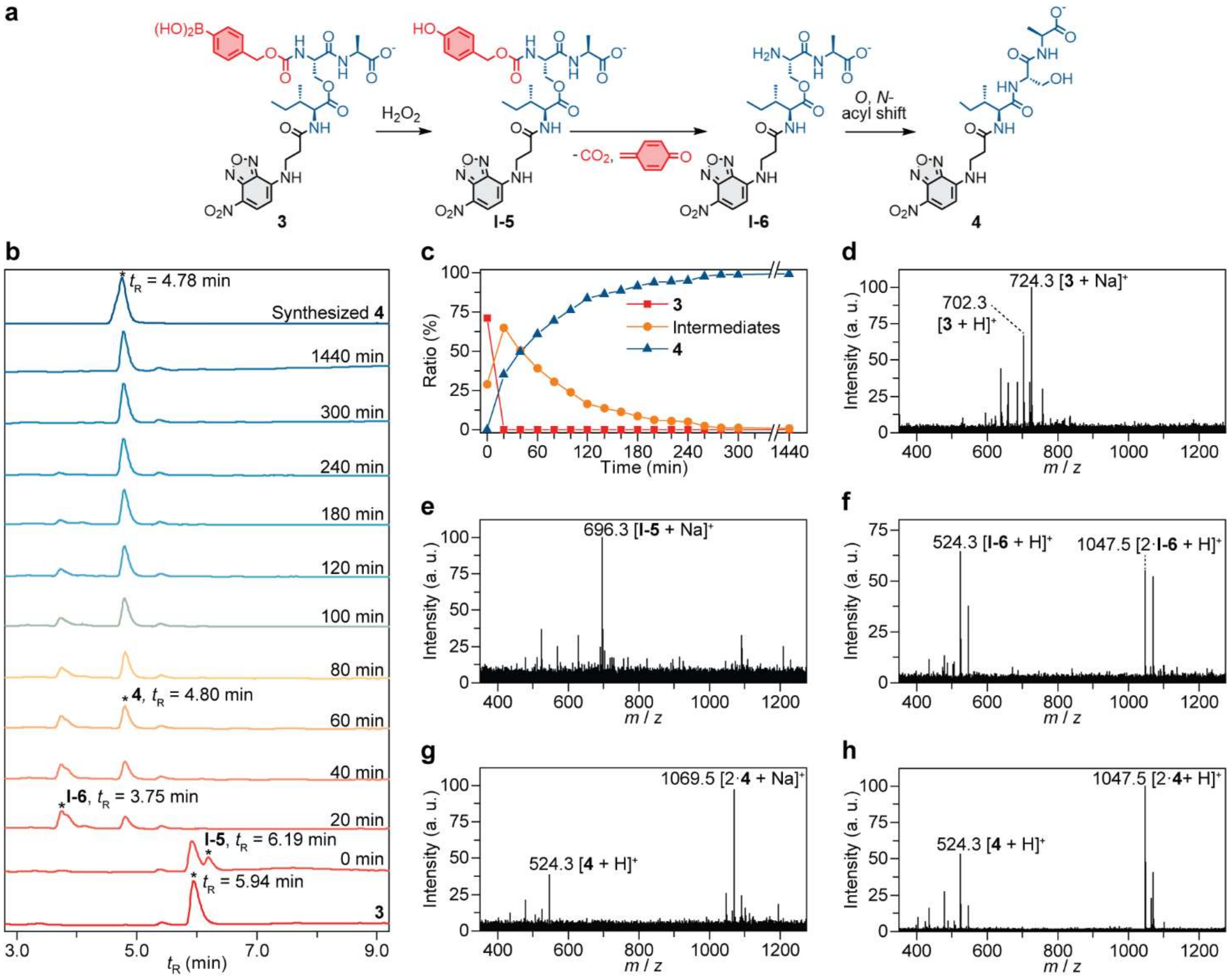

Supplementary Fig. 27| HPLC-MS kinetics analysis for H_2O_2 induced conversion from 3 to 4. a, Reaction scheme for the conversion. The oxidation of the phenylboronic acid by H_2O_2 leads to the formation of intermediate **I-5**, followed by the self-immolation of the carbamate linker to generate **I-6**. The isopeptide bond then undergo *O*, *N*-acyl shift to afford compound **4**. **b**, HPLC spectra for H_2O_2 (1.0 mM) induced-conversion of **3** (100 μM) to **4** in a mixture of NH_4HCO_3 buffer (pH 7.4, 20 mM) and tetrahydrofuran (*v*:*v* = 1:1). **c**, Molar ratio of precursor **3**, intermediates, and final product **4** after incubating with H_2O_2 based on the peak integration at 254 nm. Deboronation reaction was completed within 20 min of incubation, followed by *O*, *N*-acyl shift that afford **4** in >95% conversion rate within 240 min of incubation. **d–h**, Convoluted MS spectra in the HPLC-MS analysis for compound **3** (t_R = 5.94 min) before addition of H_2O_2 (**d**), a reaction time of 20 min and t_R = 6.19 min (**e**, identified as compound **I-5**), a reaction time of 20 min and t_R = 3.75 min (**f**, identified as compound **I-6**), a reaction time of 40 min and t_R = 4.80 min (**g**, identified as compound **4**), and compound **4** obtained by synthesis (t_R = 4.78 min) (**h**).

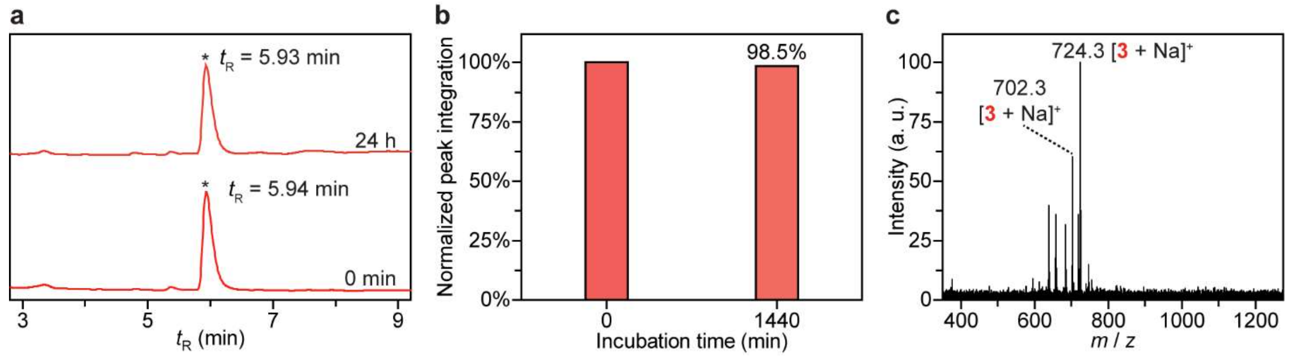

Supplementary Fig. 28| Stability of compound 3. a, Comparison of HPLC spectra of compound **3** (100 μM) incubated in a mixture of NH_4HCO_3 buffer (pH 7.4, 20 mM) and tetrahydrofuran (*v*:*v* = 1:1) for 0 and 1440 min. **b**, Normalized peak integration of the HPLC traces of compound **3** before and after the incubation for 1440 min, , which revealed that 98.5% of the compound **3** remained in the mixture. **c**, Convoluted MS spectrum of t_R = 5.93 min after incubation for 1440 min. The peak was identified as compound **3**.

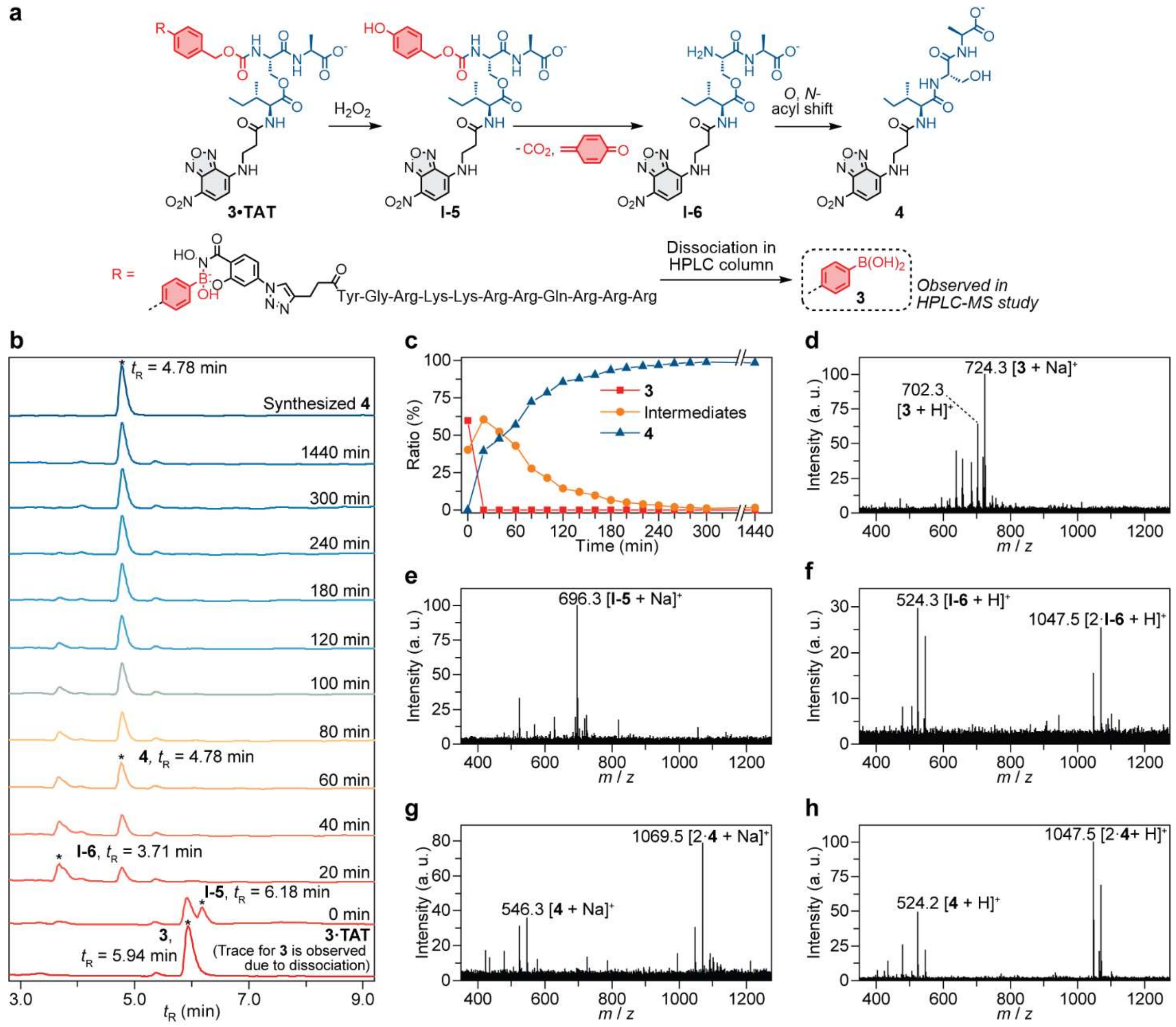

Supplementary Fig. 29| HPLC-MS kinetics analysis for H_2O_2 induced conversion from 3·TAT to 4. a, Reaction scheme for the conversion. The oxidation of the phenylboronic acid by H_2O_2 leads to the formation of intermediate **I-5**, followed by the self-immolation of the carbamate linker to generate **I-6**. The isopeptide bond then undergo *O*, *N*-acyl shift to afford compound **4**. **b**, HPLC spectra for H_2O_2 (1.0 mM) induced-conversion of **3·TAT** (100 μM) to **4** in a mixture of NH_4HCO_3 buffer (pH 7.4, 20 mM) and tetrahydrofuran (*v*:*v* = 1:1). **c**, Molar ratio of precursor **3**, intermediates and final product **4** after incubating with H_2O_2 based on the peak integration at 254 nm. Deboronation reaction was completed within 20 min of incubation, followed by *O*, *N*-acyl shift that afford **4** in >95% conversion rate within 240 min of incubation. **d–h**, Convoluted MS spectra in the HPLC-MS analysis for compound **3** (t_R = 5.94 min) before addition of H_2O_2 (**d**), a reaction time of 20 min and t_R = 6.18 min (**e**, identified as compound **I-5**), a reaction time of 20 min and t_R = 3.71 min (**f**, identified as compound **I-6**), a reaction time of 40 min and t_R = 4.78 min (**g**, identified as compound **4**), and compound **4** obtained by synthesis (t_R = 4.78 min) (**h**). As the boronic acid-SHA dynamic covalent interaction dissociates in the acidic mobile phase of the HPLC, only trace correspond to **3** can be observed after the injection of **3·TAT** to the HPLC.

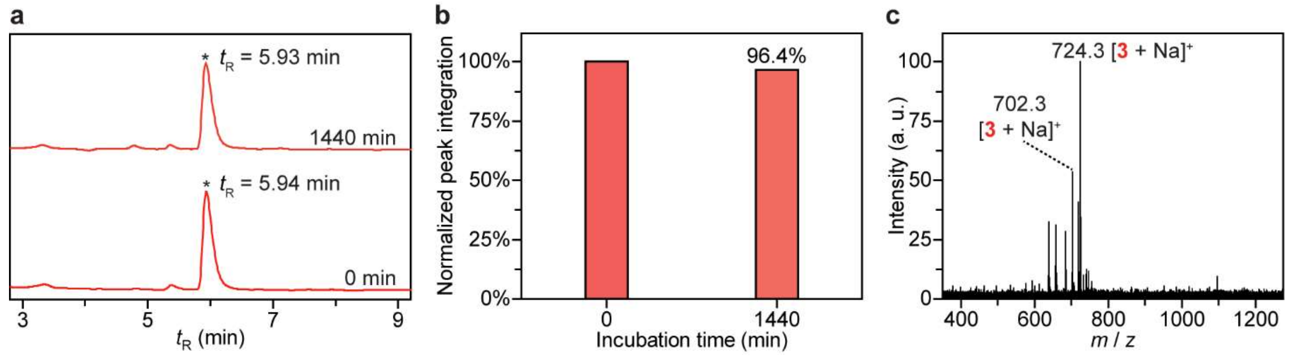

Supplementary Fig. 30| Stability of compound 3·TAT. a, Comparison of HPLC spectra of compound **3·TAT** (100 μM) incubated in a mixture of NH_4HCO_3 buffer (pH 7.4, 20 mM) and tetrahydrofuran (*v*:*v* = 1:1) for 0 and 1440 min. **b**, Normalized peak integration of the HPLC traces of compound **3** before and after the incubation for 1440 min, which revealed that 96.4% of the compound **3·TAT** remained in the mixture. **c**, Convoluted MS spectrum of t_R = 5.93 min after incubation for 1440 min. The peak was identified as compound **3**. As the boronic acid-SHA dynamic covalent interaction dissociates in the acidic mobile phase of the HPLC, only trace correspond to **3** can be observed after the injection of **3·TAT** to the HPLC.

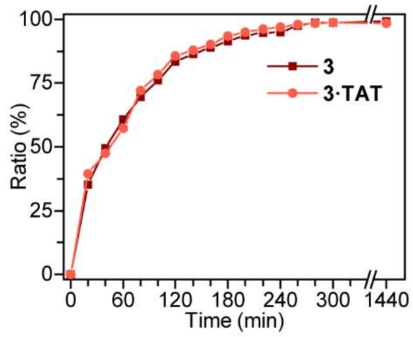

Supplementary Fig. 31| Comparison of conversion rates of final compound **4** generated by H_2O_2-induced conversion of **3** and **3·TAT** based on the peak integration at 254 nm in the corresponding HPLC spectra**.**

4. Photophysical properties of 3 and 4

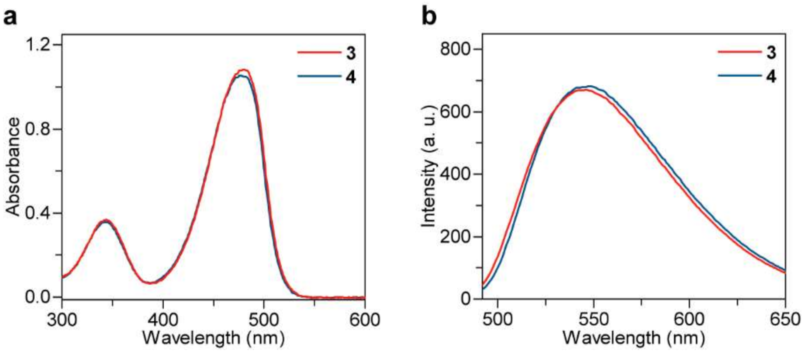

Supplementary Fig. 32| Absorption and fluorescence emission profiles of compounds 3 and 4. a, UV-Vis absorption spectra of **3** and **4** (25 μM) in phosphate buffer (PB) (pH 7.4, 50 mM). **b**, Fluorescence emission spectra of **3** and **4** (25 μM) in PB (pH 7.4, 50 mM). Excitation wavelength = 468 nm.

5. Self-assembly profiles of 2_{NF}

Monomeric complex **2** was dissolved in DMSO at a high concentration (10 mM) before diluted to different concentrations using PB (pH 7.4, 50 mM). Self-assembly formation of $\mathbf{2}_{NF}$ was induced by agitating the solution in an Eppendorf Thermomixer for 2 h at 37 °C at 500 r.p.m.

5.1 TEM analysis of the self-assemblies

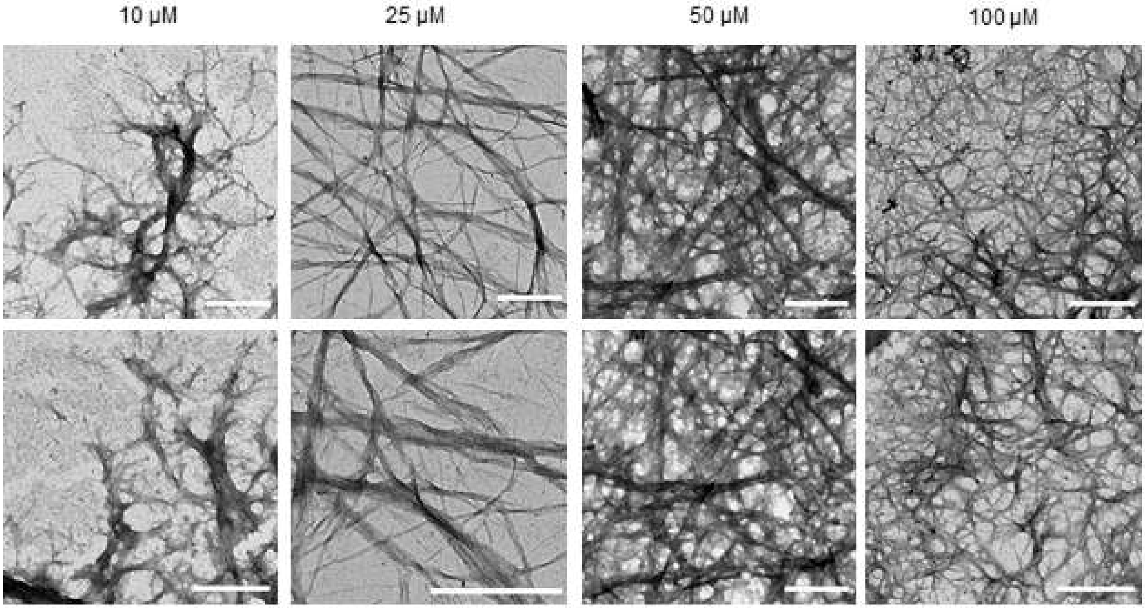

Supplementary Fig. 33| TEM micrographs of the nanofiber. Compound **2** at various concentrations was dissolved in DPBS and incubated for 10 h, before drop-casted on a carbon grid and imaged using TEM. Scale bars, 500 nm.

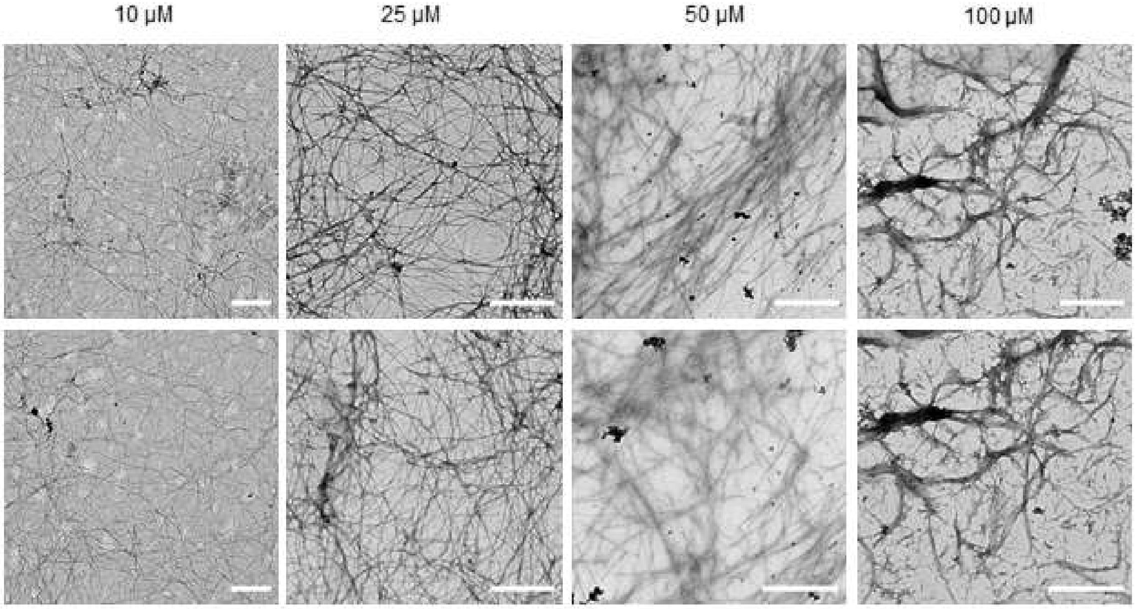

Supplementary Fig. 34| TEM micrographs of the nanofiber form upon H_2O_2-induced conversion of **1**. **1** and H_2O_2 (10 equiv.) at various concentrations were incubated in DPBS for 10 hours. The reaction mixture was then drop-casted on a carbon grid and imaged using TEM. Scale bars, 500 nm.

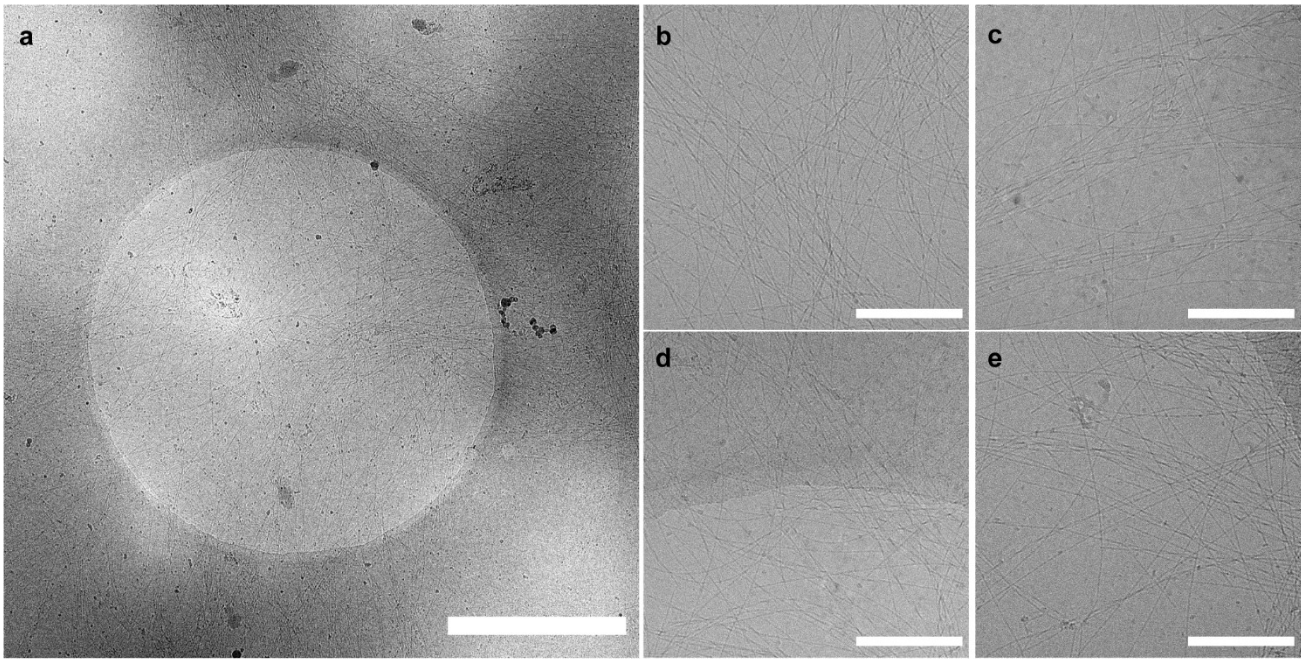

Supplementary Fig. 35| Cryo TEM micrographs of the nanofibers. Complex **2** at 100 μM was dissolved in DPBS and incubated for 1 h, Scale bars, **a**, 500 nm; **b**-**e**, 100 nm.

5.2 Determination of critical aggregation concentration (CAC)

The critical aggregation of compound 2 was determined using Nile Red dye (9-diethylamino-5-benzo[α]- phenoxazinone), a well-known fluorogenic sensor for peptide aggregation.[3] Nile Red and compound **2** were mixed in DPBS. The concentration of Nile Red was maintained at 250 nM, while the concentrations of compound **2** were varied. The maximum emission wavelengths (λ_{max}) of the mixture, originating from the Nile Red dye (λ_{ex} = 550 nm), were recorded. The λ_{max} values were plotted as a function of the concentration of compound **2**, and the CAC values corresponded to the lowest concentration at which a blueshift was observed.

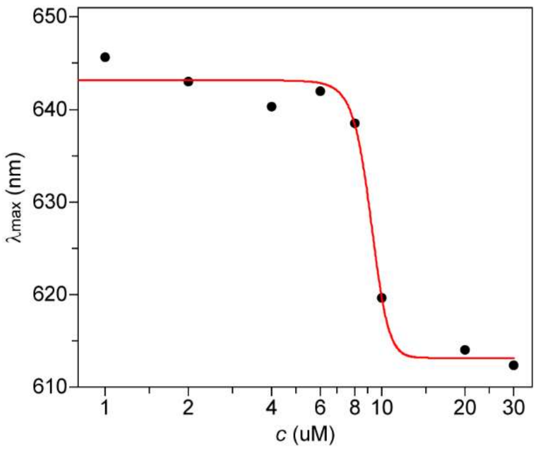

Supplementary Fig. 36| Critical aggregation concentration (CAC) study of compound 2. The shifts in maximun emission wavelength (λ_{max}) as a function of concentration of **2** were plotted, and the CAC value (~9 μM) represented the lowest concentrations at which a redshift was observed. The maximum represents the average of three measurements.

5.3 DLS analysis

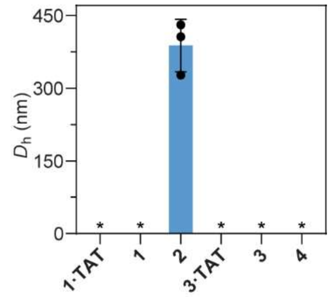

Supplementary Fig. 37| DLS analysis of the synthesised compounds at 25 μM in DPBS. a, **1·TAT**. **b**, **1**. **c**, **3·TAT**. **d**, **3**. Data are presented as mean ± s.d., n = 3. * The count rate was below detection limits for analysis, suggesting that there were no nanosized aggregates present in the solution.

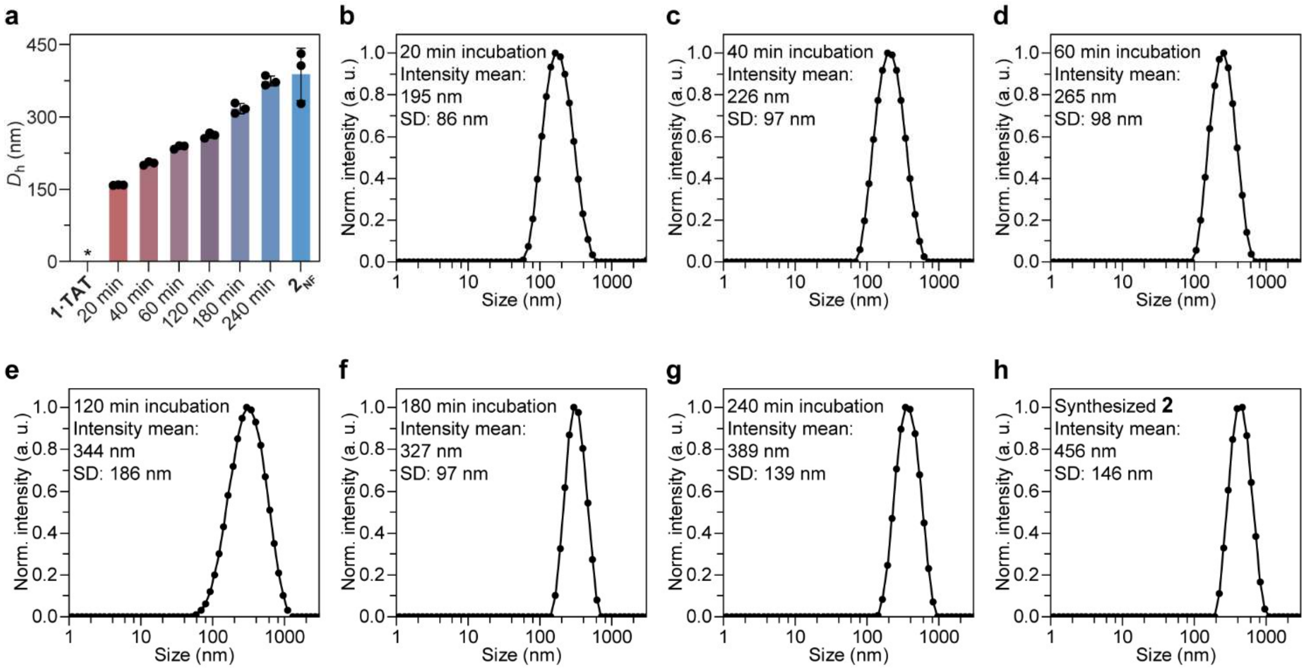

Supplementary Fig. 38| Time-dependent DLS assay of 1·TAT (25 μM) with H_2O_2 (0.5 mM) in DPBS. **a**, Hydrodynamic diameter (D_h) of the solution of **1·TAT** before and after incubation with H_2O_2 for varying amount of time, and solution of synthesized compound **2**. Data are presented as mean ± s.d., n = 3. *The count rate was too low for analysis, suggesting that there were no nanosized aggregates present in the solution. **b**-**h**, Intensity mean results obtained through the DLS analysis.

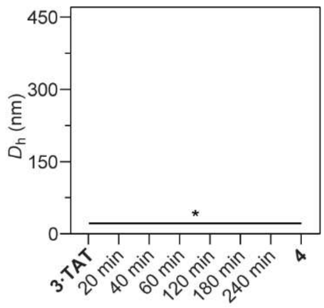

Supplementary Fig. 39| Time-dependent DLS assay of 3·TAT (25 μM) with H_2O_2 (0.5 mM) in DPBS. Hydrodynamic diameter (D_h) of the solution of **3·TAT** before and after incubation with H_2O_2 for varying amount of time, and solution of synthesized compound **4**. *The count rate was too low for analysis, suggesting that there were no nanosized aggregates present in the solution.

5.4 Variable temperature NMR analysis of the self-assemblies

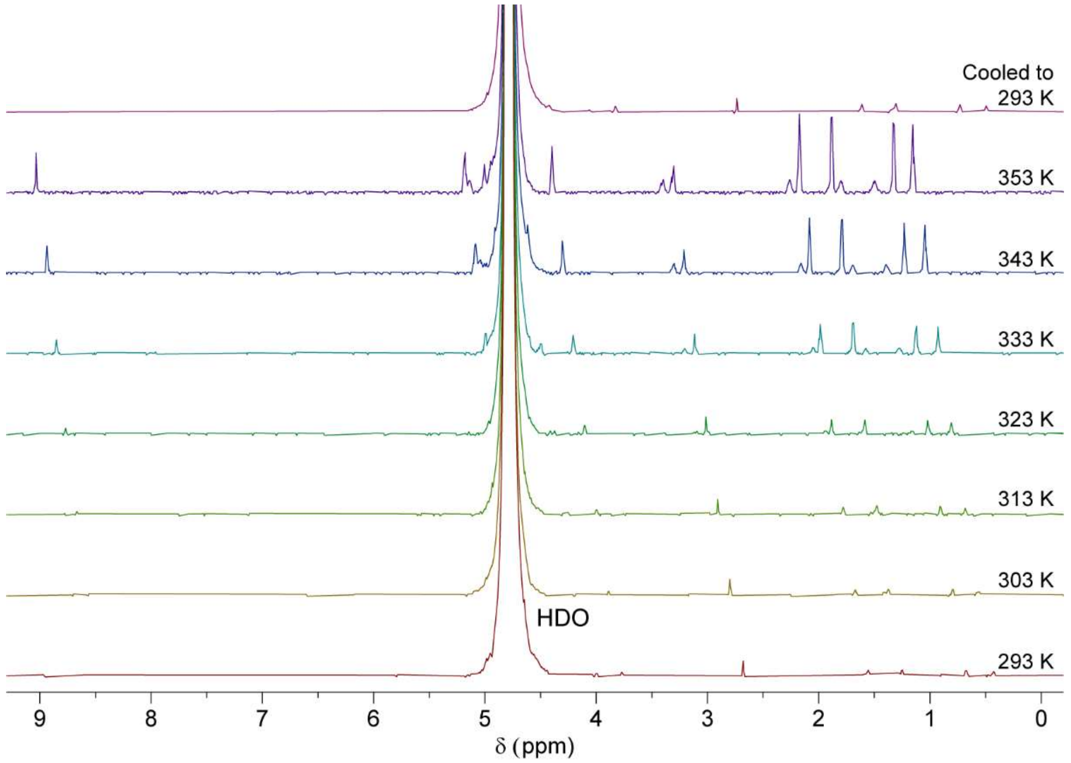

Supplementary Fig. 40| Variable temperature ^{1}H NMR spectra [700 MHz, PB (pH = 7.4, 50 mM) in D_2O] of compound 2 (250 μM). At 293 K, broad overlapping signals are observed in both aromatic and aliphatic regions, suggesting a strong self-assembly tendency of compound **2**. This phenomenon is driven by a combination of π–π interactions originating from the cNDI core, as well as hydrophobic interactions and hydrogen bond interactions arising from the peptide, resulting in the formation of nanofibers $\mathbf{2_{NF}}$. Increasing the temperature of the measurement results in the sharpening of the signals and increased signal intensity, which is indicative of disassembly of the nanostructures. Cooling the sample back to 293 K led to the recovery of broad overlapping signals, suggesting the reversible nature of the self-assembly.

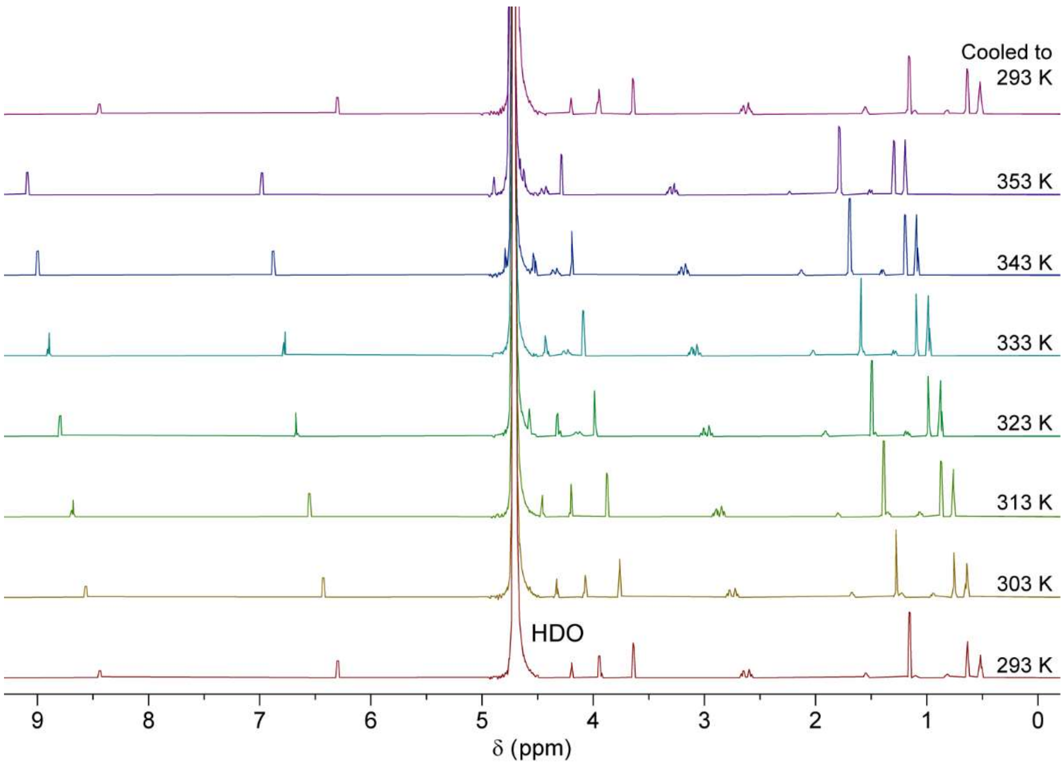

Supplementary Fig. 41| Variable temperature ^{1}H NMR spectra [700 MHz, PB (pH = 7.4, 50 mM) in D_2O] of compound 4 (500 μM). At 293 K, well-resolved peaks are observed for the sample that allowed for detialed peak assginment. Heating the sample up to 353K resulted in minimal changes in peak intensity, indicating no self-assembly at 293K.

5.5 Fluorescence lifetime imaging of assembled fibers in a Cell-Free system

After **2** was dissolved in DMSO at a concentration of 10 mM, the stock solution was diluted to 120 μM with Dulbecco's Modified Eagle Medium (DMEM). After 2 h incubation with shaking at room temperature, the solution was added into an 8-well confocal plate. The samples were imaged using phasor-resolved fluorescence lifetime imaging microscopy (phasor-FLIM). Three different regions were imaged and analyzed (Fig. 3f-k and Supplementary Figs. 42-43).

For the kinetics analysis for the H_2O_2-induced conversion of **1·TAT** to **2$_{NF}$**. A mixture of **1·TAT** (120 μM) and H_2O_2 (1.2 mM) was prepared using DMEM. The mixture was added into an 8-well confocal plate and imaged using phasor-resolved fluorescence lifetime imaging microscopy (phasor-FLIM). Imaging parameters are identical to those found in point **1.2.13.**

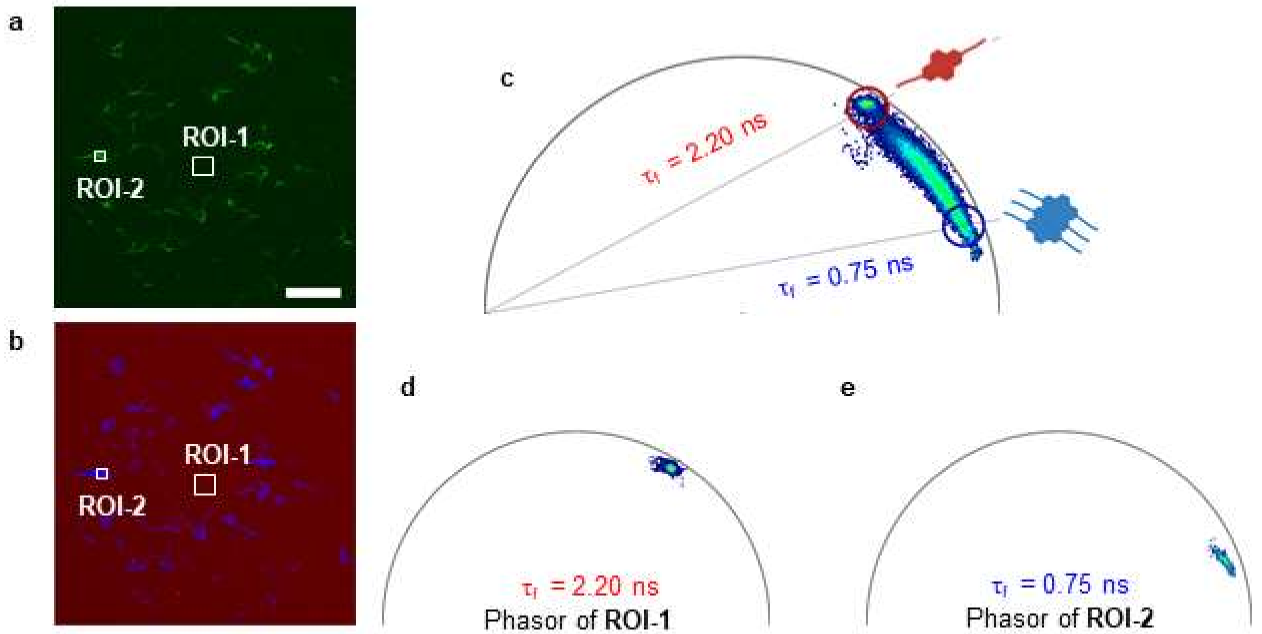

Supplementary Fig. 42| **a**, Intensity-based confocal image for **2$_{NF}$** in a cell-free condition. λ_{ex} = 469 nm, λ_{em} = 500-520 nm. Scale bar, 20 μm. **b**, Corresponding fluorescence lifetime image for **2$_{NF}$**. Red channel indicates species with a τ_f = 2.20 ns, and blue channel indicates species with a τ_f = 0.75 ns. **c**, Phasor plots of ROI-1 (monomers) in DMEM, τ_f = 2.20 ns. **d**, Phasor plots of ROI-2 (assemblies) in DMEM, τ_f = 0.75 ns. **h**, Fluorescence lifetime analysis of monomers and assemblies in cell free system using phasor analysis. Two major species showing τ_f = 2.20 and 0.75 ns, respectively, can be identified.

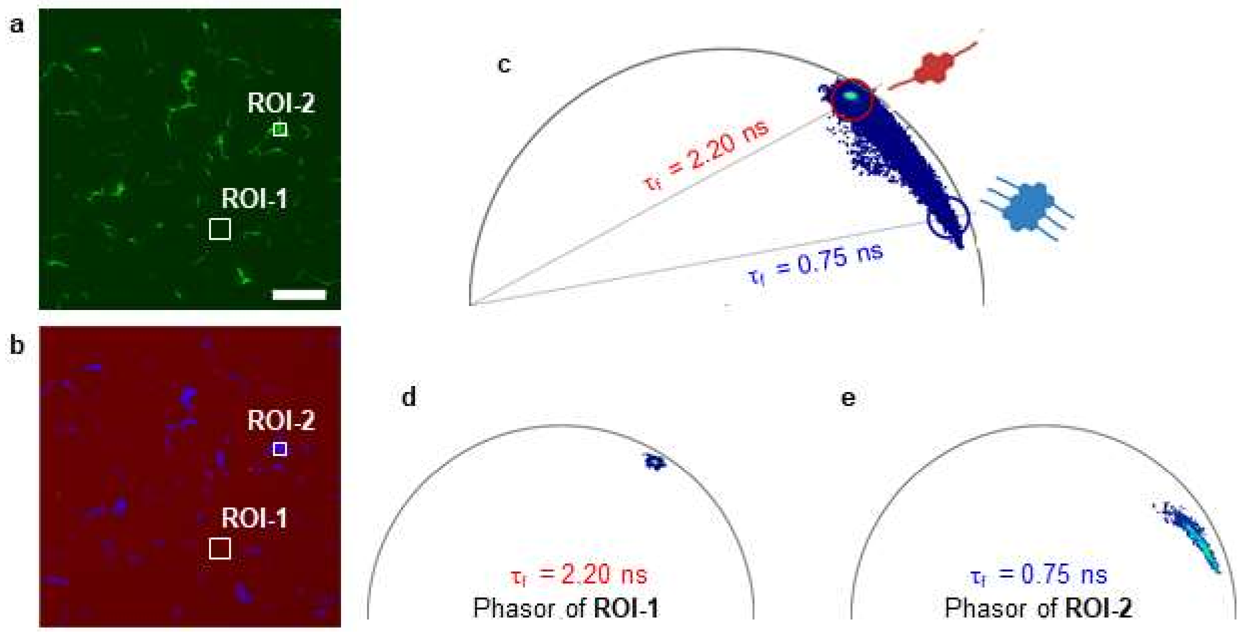

Supplementary Fig. 43| **a**, Intensity-based confocal image for **2$_{NF}$** in a cell-free condition. λ_{ex} = 469 nm, λ_{em} = 500-520 nm. Scale bar, 20 μm. **b**, Corresponding fluorescence lifetime image for **2$_{NF}$**. Red channel indicates species with a τ_f = 2.20 ns, and blue channel indicates species with a τ_f = 0.75 ns. **c**, Phasor plots of ROI-1 (monomers) in DMEM, τ_f = 2.20 ns. **d**, Phasor plots of ROI-2 (assemblies) in DMEM, τ_f = 0.75 ns. **h**,

Fluorescence lifetime analysis of monomers and assemblies in cell free system using phasor analysis. Two major species showing τ_f = 2.20 and 0.75 ns, respectively, can be identified.

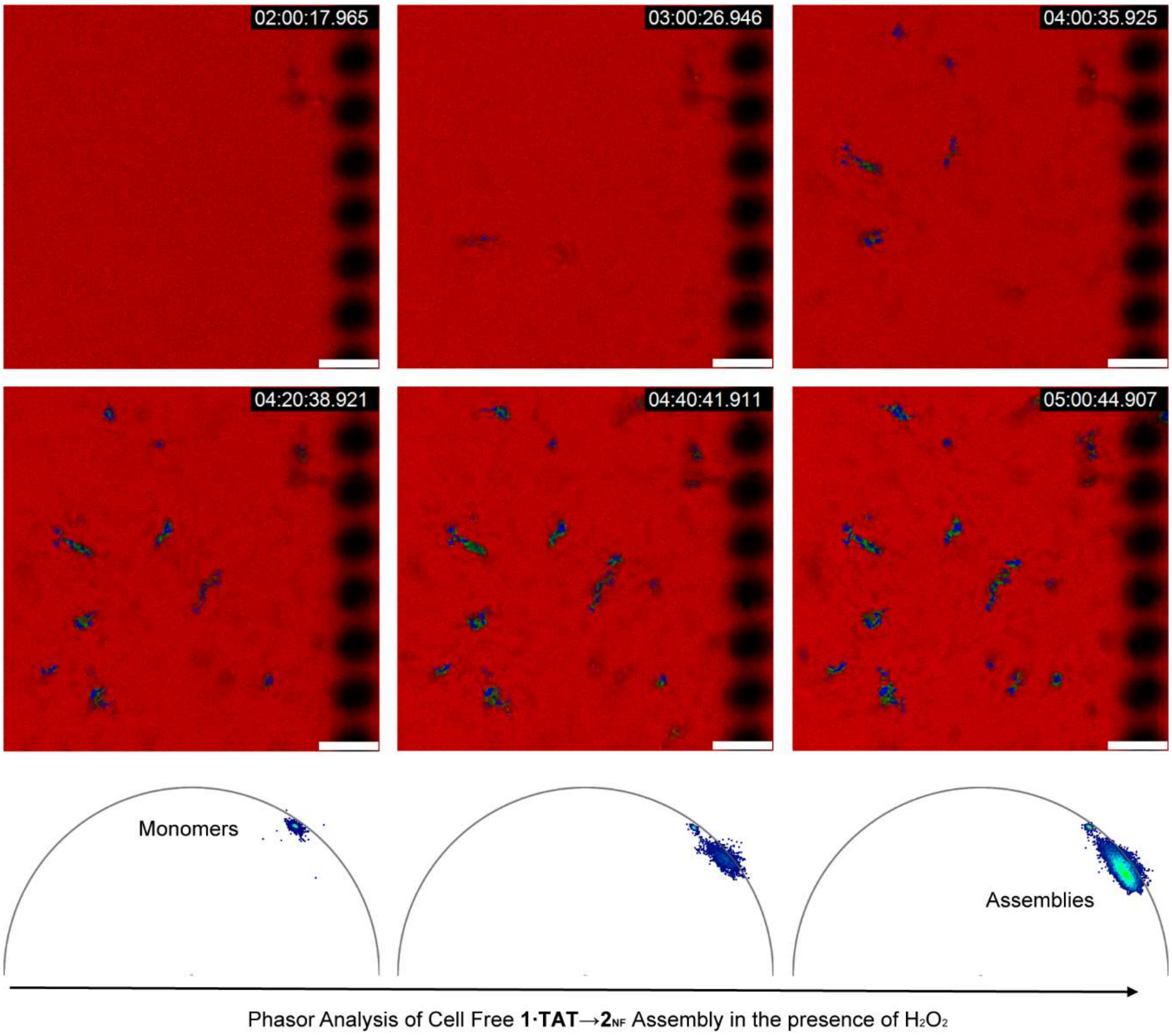

Supplementary Fig. 44| Time lapsed snapshots and phasor analysis of **1·TAT→2$_{NF}$** in the presence of H_2O_2 in cell free system. Red: monomers, Blue: assemblies. Nanofibers **2$_{NF}$** can be identified after 3h incubation with H_2O_2. Scale bars: 20 μm.

6 Cellular uptake of 1·TAT and 3·TAT and intracellular self-assembly of 1·TAT

6.1 Cellular uptake of 1·TAT

MDA-MB-231 cells were seeded at a density of 30,000 cells/well in an 8-well confocal plate. After adhering for 24 h, cells were treated with the sample for 4 h at 37 °C. Before adding to the cells, samples were pre-incubated to form the **1·TAT** by dissolving **1** in DMSO and mixing with two equimolar amount of **TAT** peptide, which was dissolved in Dulbecco's PBS. The sample was further diluted with DPBS to a final volume of 40 μL (DPBS:DMSO 9:1). Sample solutions were further diluted 1:4 with DMEM and added to the cells (total DMSO content = 2%), after removing the existing medium from the wells. After the incubation time was over, the cell mitochondria was stained with MitoTracker ® Deep Red for 30 min at 37 °C and the cell nucleus was stained with HCS NuclearMask™ Stains for

20 min at 37 °C. The staining solution was removed, and fresh FluoroBrite DMEM Medium was added to the cells before they were imaged by confocal laser scanning microscopy.

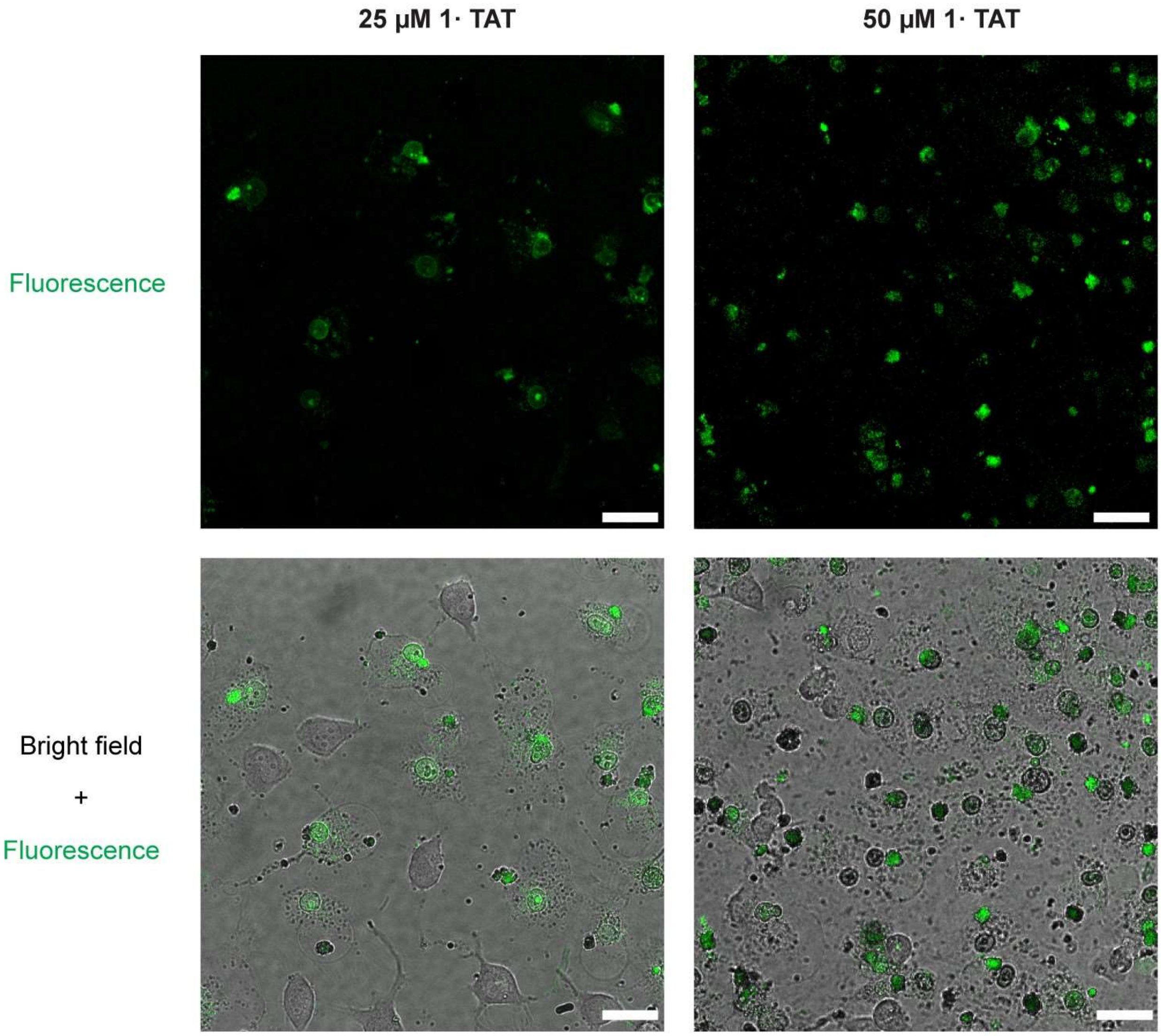

Supplementary Fig. 45| CLSM images of MDA-MB-231 cells treated for 4 h with **1·TAT** (25 μM and 50 μM). Scale bars, 20 μm.

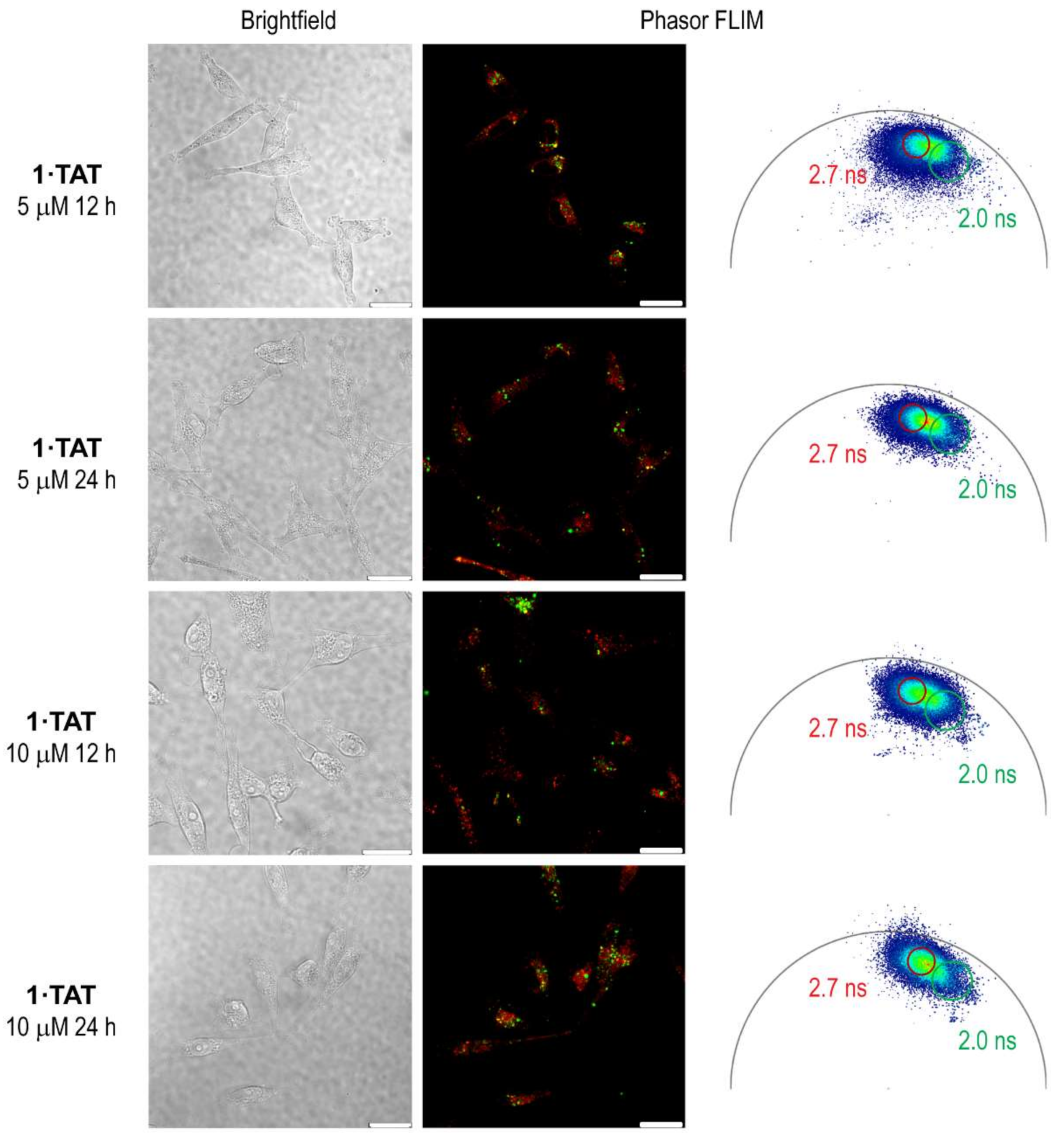

Supplementary Fig. 46| Phasor-FLIM analysis of MDA-MB-231 cells treated with **1·TAT** (5 μM and 10 μM) for 12 h and 24 h. Scale bars, 20 μm.

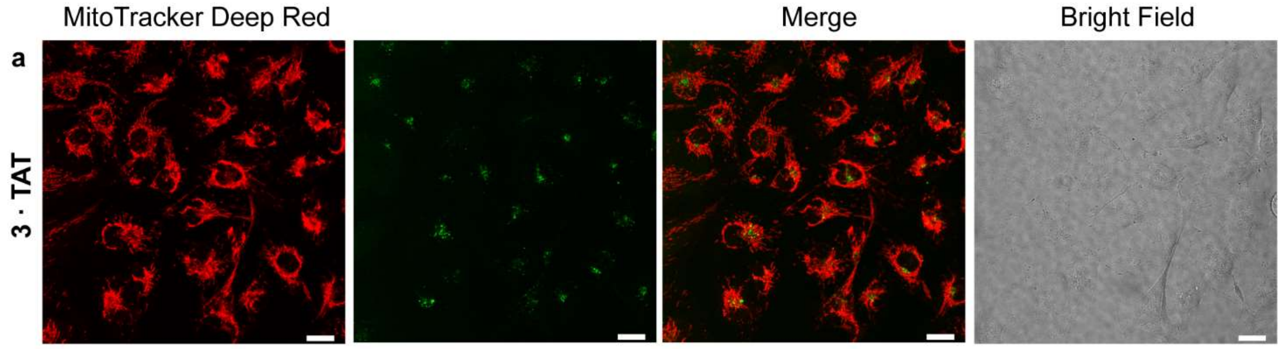

Supplementary Fig. 47| a CLSM images of MDA-MB-231 cells treated for 4 h with **3·TAT** (25 μM) and MitoTracker ® Deep Red, Scale bars, 20 μm.

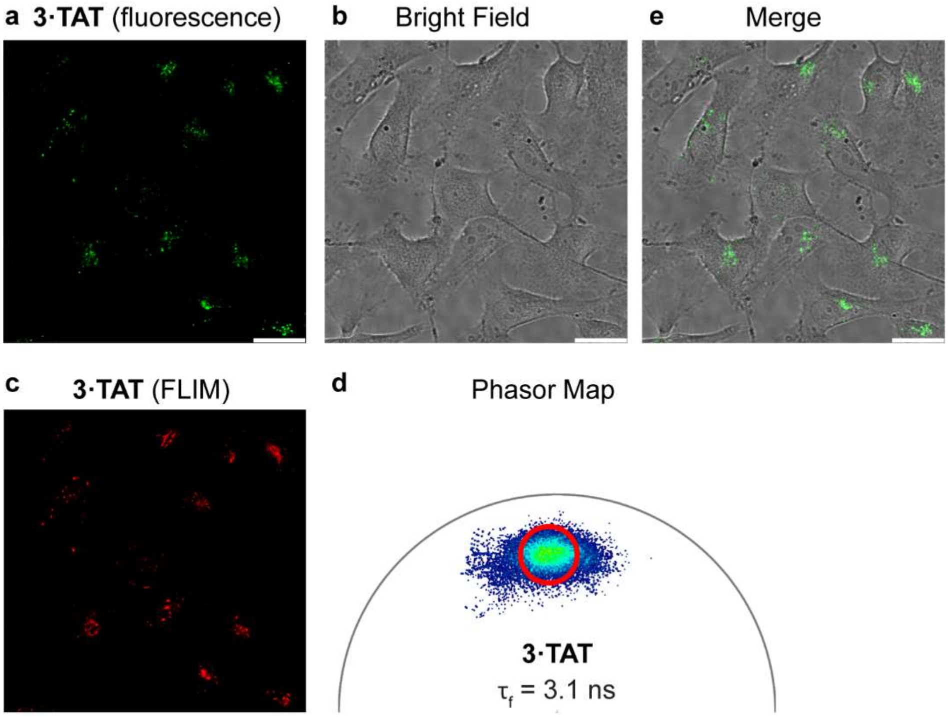

Supplementary Fig. 48| Phasor-FLIM analysis of MDA-MB-231 cells treated with 3·TAT (25 µM) for 4 h. Scale bars, 20 µm.

6.2 Electron microscopy and correlative light- and electronmicroscopy of cells

For electron microscopy (EM) studies and correlation with confocal light microscopy, MDA-MB-231 cells were seeded at a density of approximately 30000 cells/well on sapphire disks and incubated with **1·TAT** as described above. Staining with NucBlue® Live ReadyProbes® was not performed. After incubation, cells on the sapphire discs were frozen using high-pressure freezing (Wohlwendt HPF 01). This was followed by freeze substitution, in which the cells were stained at low temperatures with OsO4 and uranyl acetate for electron microscopic examination. Then, the cells were embedded in epoxy resin (EPON) and finally, ultrathin sections were prepared in an ultramicrotome (Leica FC7) using a diamond knife (Diatome). The thickness of the thin sections and the substrate onto which the sections were transferred were adjusted to meet the requirements of the subsequent EM investigation. For TEM and TEM tomography, the sections were transferred to Cu grids. The nominal section thickness for TEM investigations was 100 nm and for TEM tomography 300 nm. For SEM investigations, the nominal section thickness was 100 nm, and the sections were transferred to ITO-coated glass coverslips for this purpose. To identify the **1·TAT** component in the EM images, we correlated its fluorescence with the EM images (correlative light and electron microscopy - CLEM). For this purpose, the fluorescence of the **1·TAT** was first measured in a confocal laser scanning microscope (Leica SP5) at a wavelength of 500-550 nm. Then, the same thin section was examined in TEM (FEI Tecnai F20) or SEM (Hitachi SU8000), and the fluorescence image was precisely superimposed on the electron microscopy image using ImageJ plugin 1.53e.

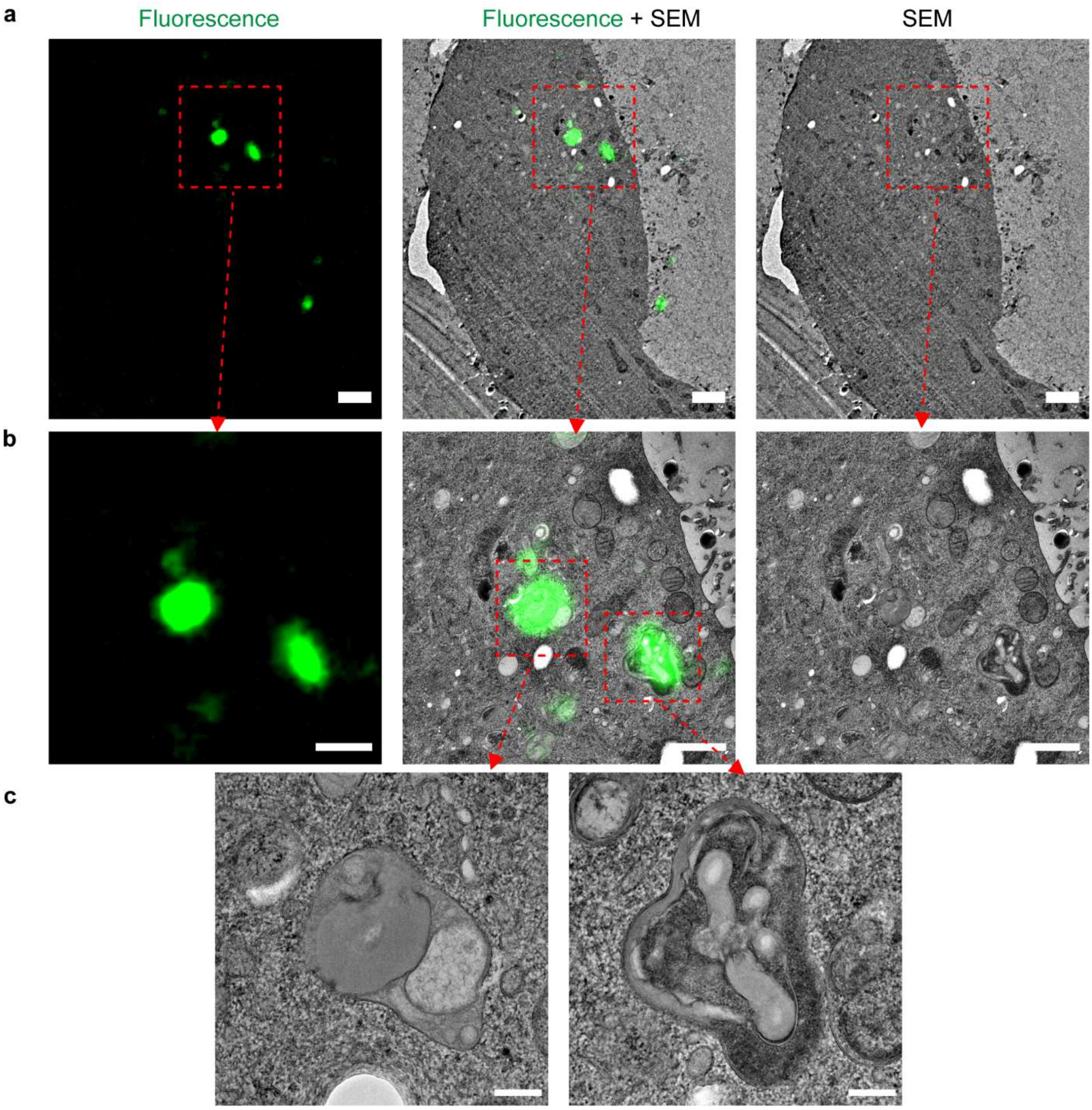

Supplementary Fig. 49| SEM-CLEM micrographs of intracellular localization within the endosomes of 1·TAT. Endosomes containing **1·TAT** are significantly distorted compared to surrounding vesicles. Magnified images show the presence of multiple phase separated sub-compartments and protofibrils. **a**, Scale bars, 2 μm. **b**, Scale bars, 1 μm. **c**, Scale bars, 200 nm.

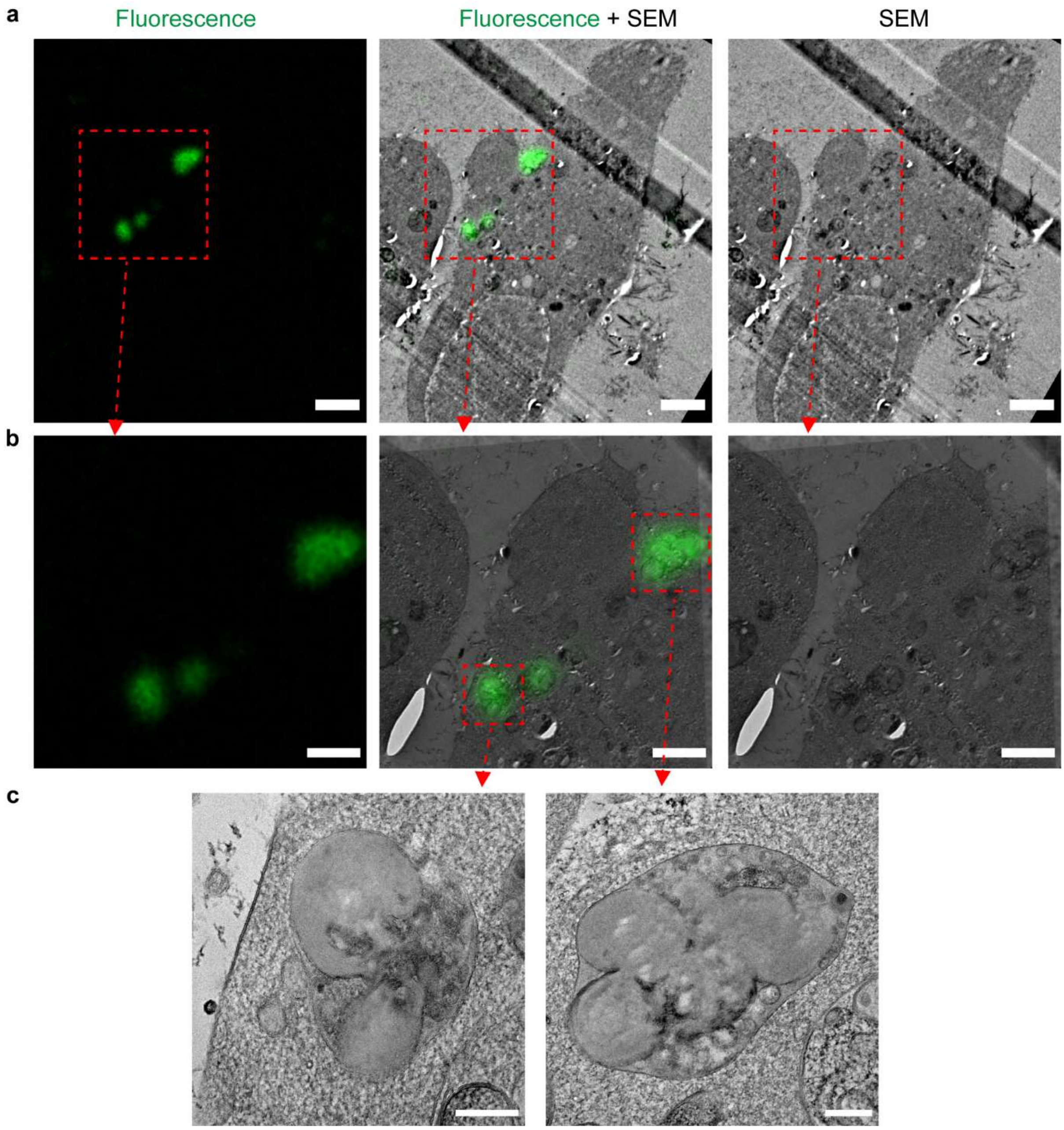

Supplementary Fig. 50| SEM – CLEM micrographs of intracellular localization within the endosomes of **1·TAT**. Endosomes containing **1·TAT** are significantly distorted compared to surrounding vesicles. Magnified images show the presence of multiple phase separated sub-compartments and protofibrils. **a**, Scale bars, 2 µm. **b**, Scale bars, 1 µm. **c**, Scale bars, 200 nm.

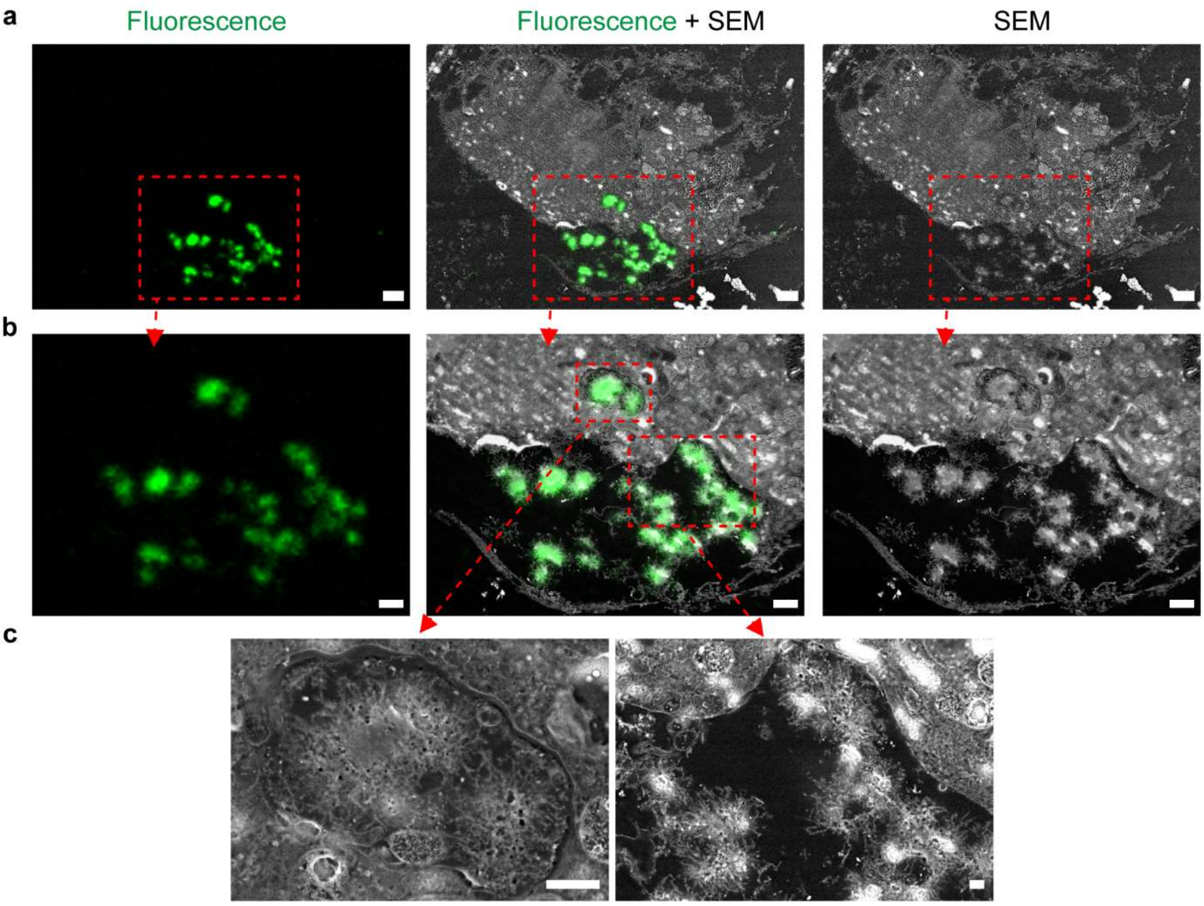

Supplementary Fig. 51| SEM – CLEM micrographs of MDA-MB-231 cells treated for 1 h with **1·TAT** (25 μM) showing the nanostructures of the characteristic dark nodules as the cell shrinks. **a**, Scale bars, 2 μm. **b**, Scale bars, 1 μm. **c**, Scale bars, 200 nm.

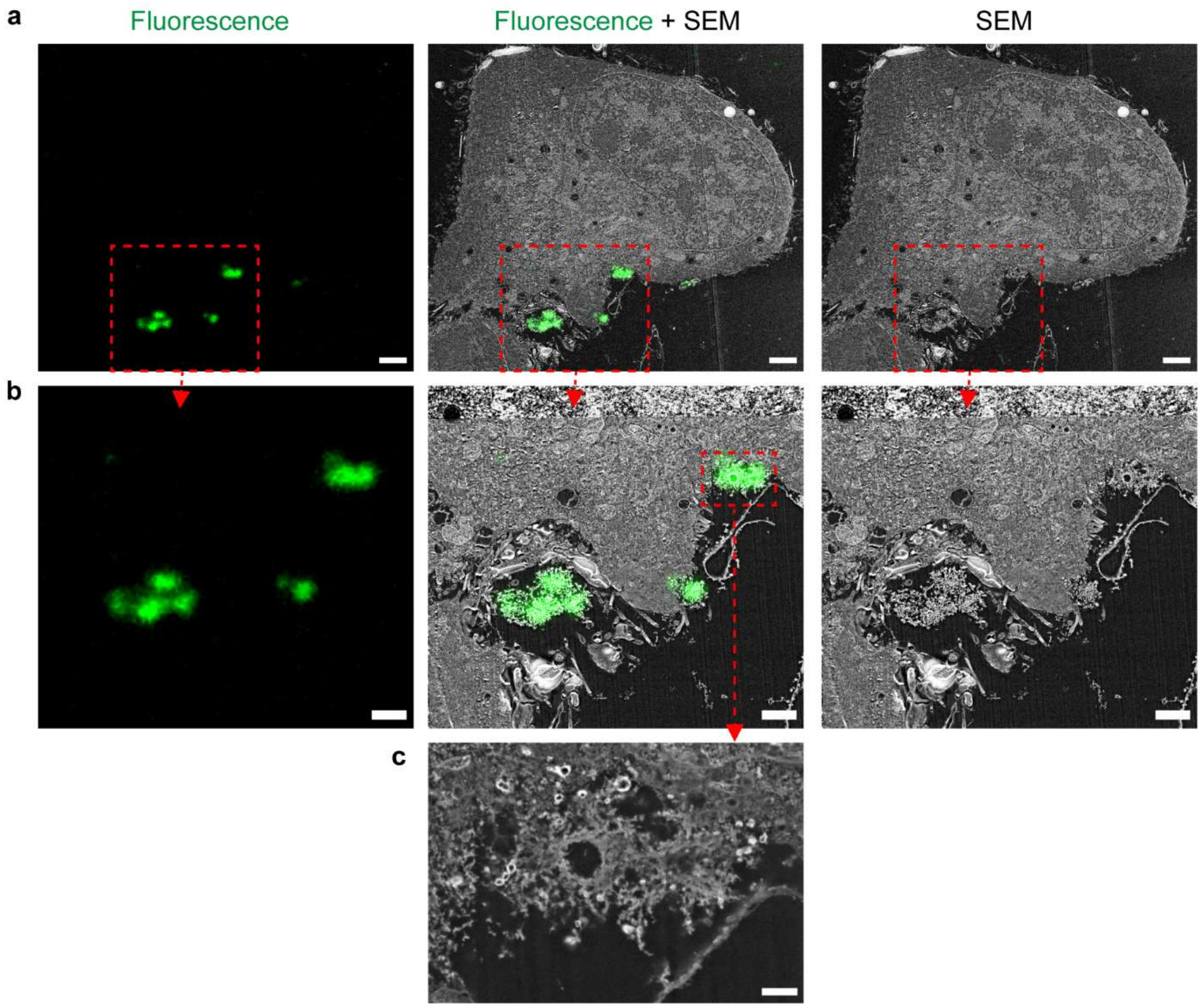

Supplementary Fig. 52| SEM – CLEM micrographs of MDA-MB-231 cells treated for 1 h with **1·TAT** (25 µM) showing the nanostructures of the characteristic dark nodules as the cell shrinks. **a**, Scale bars, 2 µm. **b**, Scale bars, 1 µm. **c**, Scale bars, 200 nm.

6.3 Tomography

Tomographic 3D reconstruction of the volume was performed using cryo-TEM. For this, 300 nm thick sections on TEM Cu grids were used as described above. Tilt series were acquired on a Thermo-Fischer Titan Krios G4 using SerialEM software (the University of Colorado Boulder). Tomographic reconstruction was performed with imod (the University of Colorado Boulder) and Tomoviz (Cornell University) was used for 3D volume visualization.

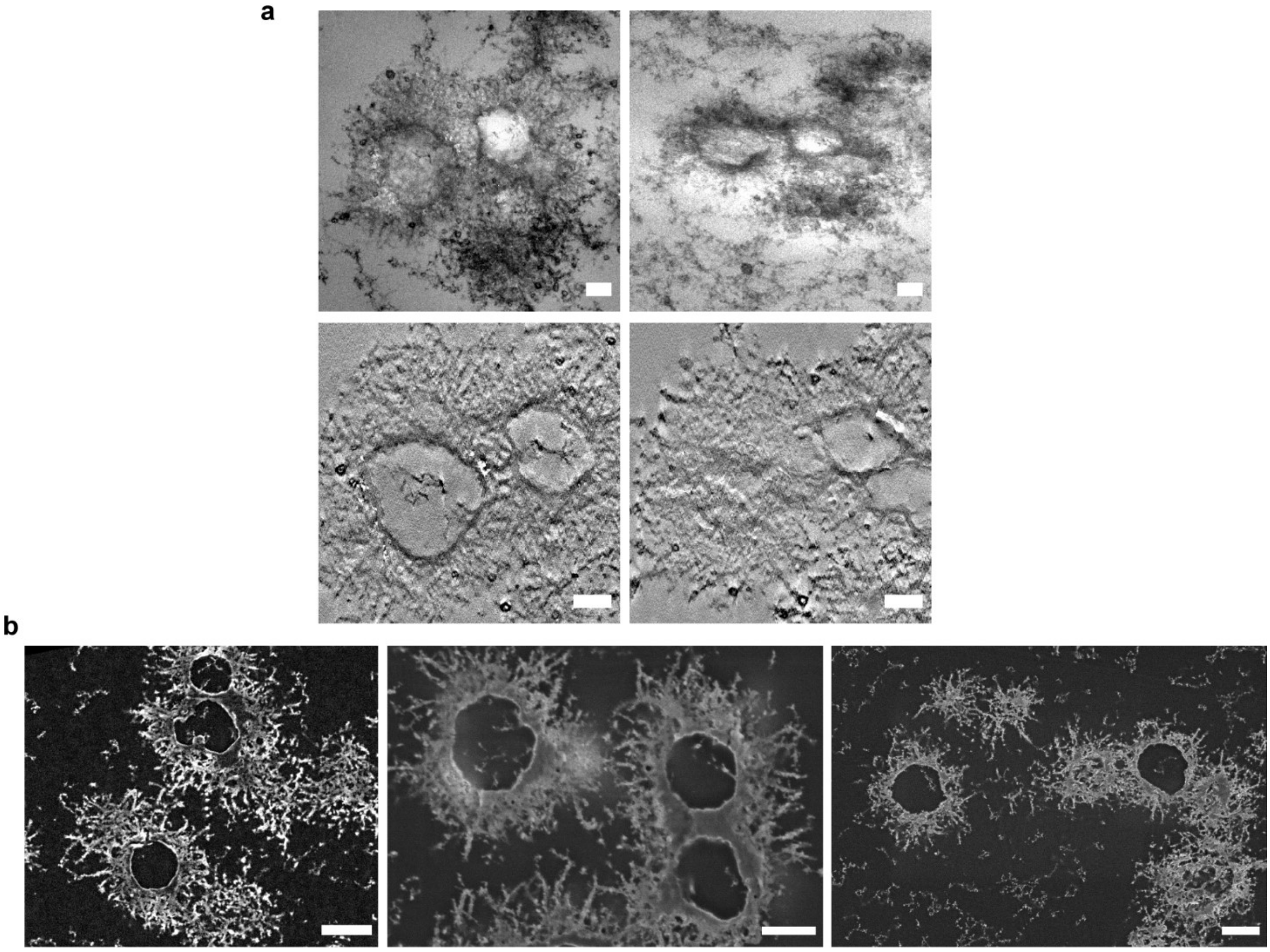

Supplementary Fig. 53| Tomography and EM micrographs of hollow spherical objects induced by 1·TAT. **a**, magnified slice of TEM tomogram, scale bars: 200 nm; **b**, SEM images of the spherical objects taken under using different parameters. Scale bars, 500 nm.

6.4 Mapping intracellular formation of 2_{NF} for live cells using Phasor-FLIM

MDA-MB-231 cells were seeded at a density of 30,000 cells/well in an 8-well confocal plate. After adhering for 24 h, cells were treated with the sample for 4 h at 37 °C. The sample was dissolved in DMSO at a concentration of 10 mM, then the sample was further diluted with DPBS to a final volume of 40 µL (DPBS: DMSO = 9:1). Sample solutions were further diluted 1:4 with DMEM and added to the cells (total DMSO content = 2%), at the same time the 8-well confocal plate was placed in the incubation box of the phasor-resolved fluorescence lifetime imaging microscopy (phasor-FLIM) and the cells were imaged in real time.

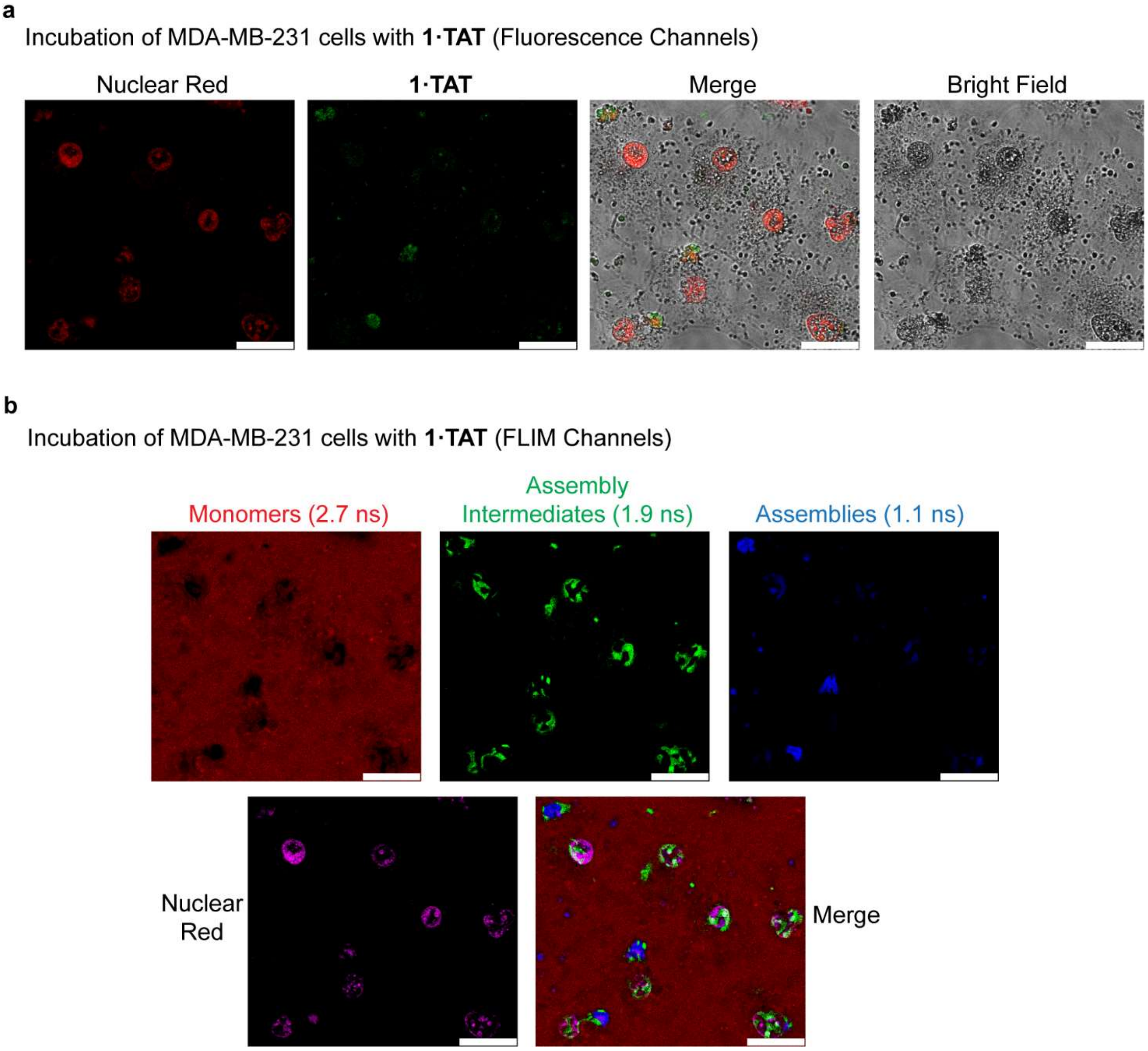

Supplementary Fig. 54| Separated channels for fluorescence lifetime imaging of MDA-MB-231 cells treated with **1·TAT** (25 μM) for 30 min. **a**, Fluorescence channels. **b**, FLIM channels. Different species including monomers, assembly intermediates, assemblies, and their merge were shown in separated images, Scale bars, 20 μm.

6.5 Stability of compound 1 in cell lysate

MDA-MB-231 cells were seeded at 15,000 cells/well in an 8-well confocal plate and subsequently washed with DPBS for three times. The cells were then lysed using of radioimmunoprecipitation assay (RIPA) lysis buffer (100 μL) on ice for 5 min. Cells were detached using a cell scraper, and the cell lysate was collected. A solution of compound **1** (1.8 μM) was prepared using the cell lysate as the solvent. To analyze the mixture, CH_3OH (400 μL) was added, and the resulting mixture was centrifuged at 13,000 rpm at 4 °C for 20 minutes to remove proteins and cellular debris. The supernatant was then analyzed using analytical HPLC (Supplementary Fig. 55).

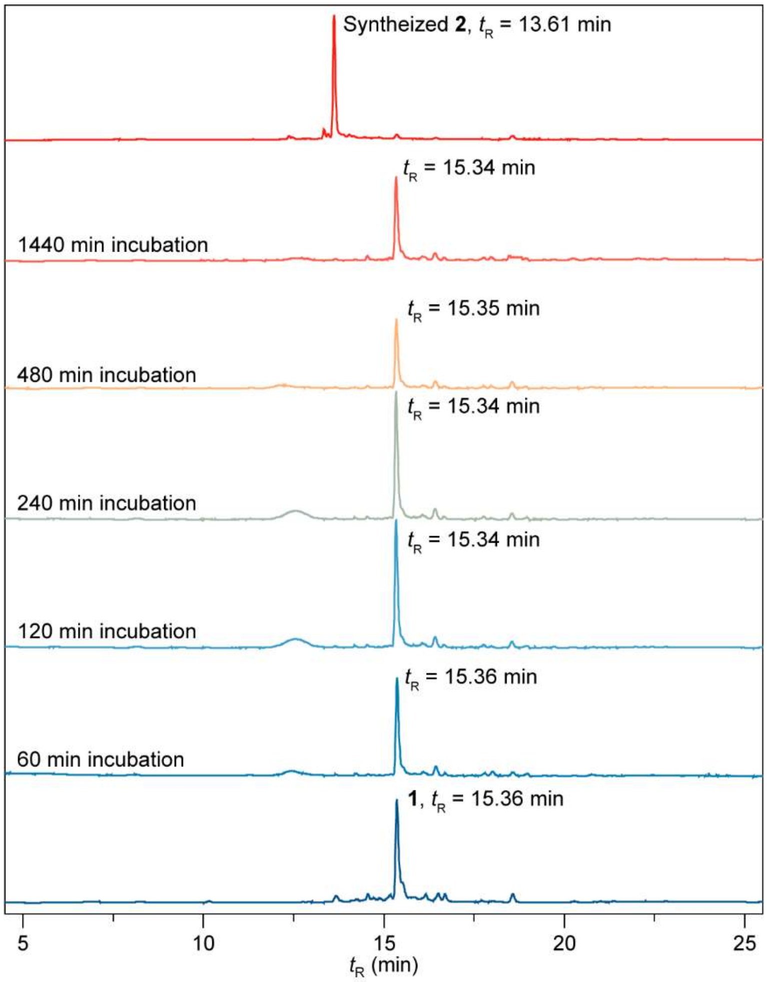

Supplementary Fig. 55| HPLC analysis for the stability of 1 in lysates of MDA-MB-231 cells. Compound **1** (1.8 μM) was incubated with cell lysate of MDA-MB-231 cells and subsequently analyzed over 24 hours. No degradation is observed during the experiment, confirming that **1** is solely responsive to H_2O_2.

6.6 HPLC analysis of intracellular transformation of 1·TAT into 2_{NF}

MDA-MB-231 cells were seeded at 15,000 cells/well in an 8-well confocal plate. The cells were incubated with **1·TAT** at various concentrations for 4 hours. After incubation, the cells were washed with DPBS three times and lysed using of RIPA lysis buffer (100 μL) on ice for 5 minutes. Cells were detached using a cell scraper, and the cell lysate was transferred into a PCR tube. To analyze the lysate, CH_3OH (400 μL) was added, and the resulting mixture was centrifuged at 13,000 rpm at 4 °C for 20 minutes to remove proteins and cellular debris. The resulting supernatant was collected and analyzed using analytical HPLC (Supplementary Fig. 56).

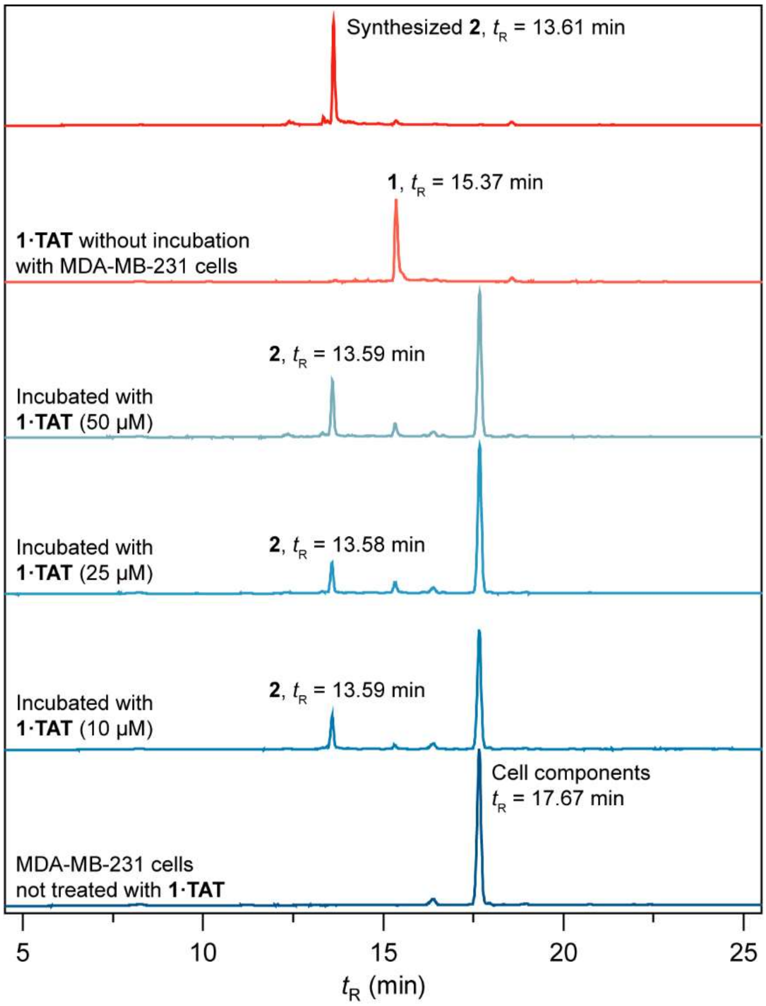

Supplementary Fig. 56| HPLC assay on the lysate of MDA-MB-231cells treated with 1·TAT. In the control group which cells are not treated with **1·TAT**, trace with retention time (t_R) = 17.67 min can be indentified. This trace can be attributed to cell components. For cells treated with **1·TAT**, peaks correpsonds to compound **2** (t_R = 13.59 min), the final product for the intracellular H_2O_2-induced conversion of **1·TAT** can be observed, suggesting endogenous H_2O_2 is sufficient for decomposing the phenylboronic acid cage and generate **2** as the final conversion product.

6.7 Phasor-FLIM analysis of 1·TAT incubated in cell lysates of MDA-MB-231 cells

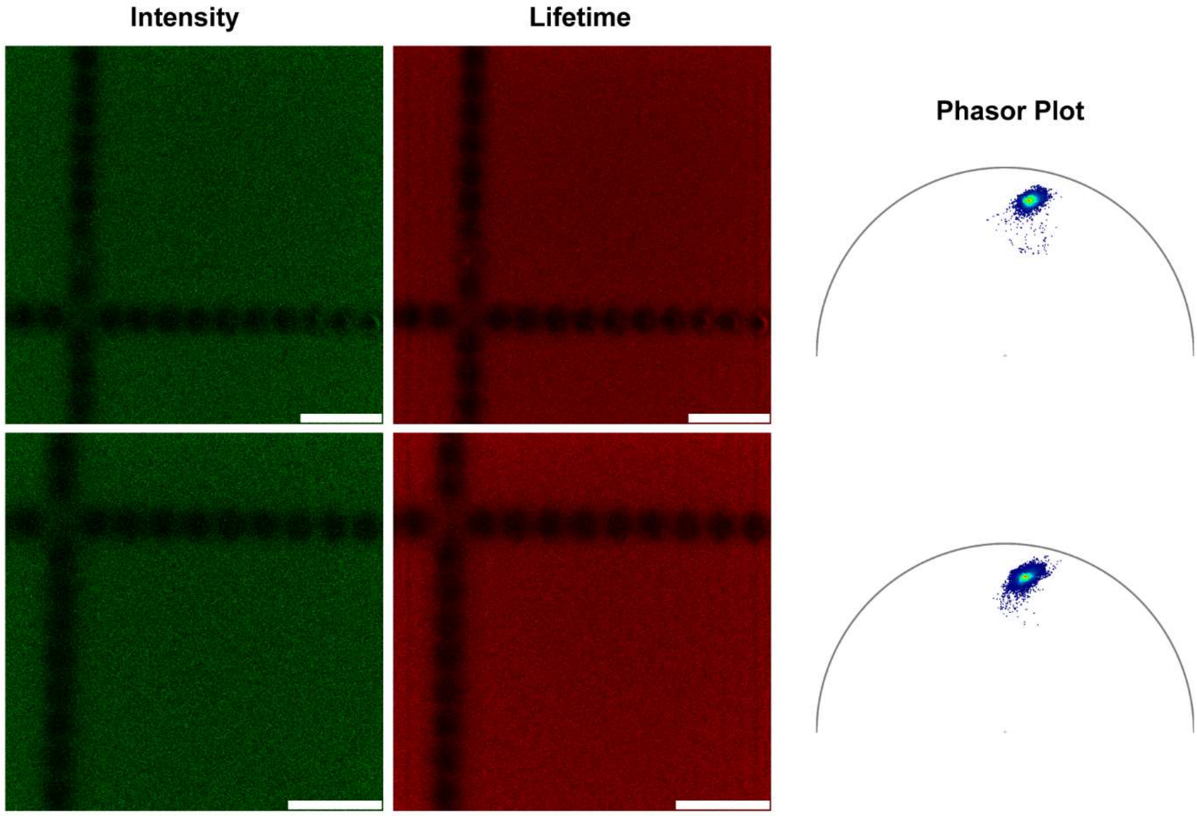

Supplementary Fig. 57| Phasor FLIM studies performed with **1·TAT** (25 µM) incubated in cell lysates of MDA-MB-231 cells for 4 h. Analysis of two different regions shows no presence of aggregation or non-specific interactions that changes the fluorescence lifetime of **1·TAT**. Phasor plots are presented with a single locus with τ = 2.9 ns. Scale bar = 50 µm.

7. Biological response of intracellular superstructure formation

7.1 Glucose Uptake-Glo^{TM} Assay

MDA-MB-231 cells were seeded in a clear-bottom 96-well plate at a density of 5,000 cells/well. They were then incubated at 37°C overnight to facilitate adherence. The following day, the cells were incubated with **3·TAT** (25 µM) for 4 h, or with **1·TAT** (25 µM) for intervals of 0.5 h, 1 h, 2 h, and 4 h. Once the incubation was completed, the medium was aspirated from the wells, and the cells were washed with DPBS (100 µL). Subsequently, 1 mM 2-deoxyglucose (2-DG) (50 µL) was added to each well. After a brief shake, the plate was allowed to incubate for 10 minutes at room temperature. Stop Buffer (25 µL) and Neutralization Buffer (25 µL) were then added to the wells, followed by a brief shake to ensure even distribution of the buffers. To complete the preparation, 2DG6P Detection Reagent (100 µL) was added to each well. After a short shake, the plate was incubated for an additional 0.5 h at room temperature. Luminescence measurements were taken using the Promega GloMax®-Multi Detection System, adhering to the manufacturer's protocol specified for luminescence readout. The result was presented in Fig. 5A in the main text.

7.2 NAD/NADH assay

MDA-MB-231 cells were both seeded at a density of 5000 cells/well in a white half area 96-well plate and incubated at 37°C overnight to allow them to adhere. On the next day, 250 μM **1·TAT** were prepared in PBS at a 10x concentration of the final concentration and of this solution (20 μL) were added to fresh medium (180 μL). The old medium of the cells was removed and substituted with fresh medium (50 μL) containing different concentrations of sample, prepared as described before. For each condition triplicates were performed. Cells were incubated with the sample for 5 min,10 min, 20 min, 30 min, 1 h, 2 h and 4 h. After the treatment, NAD/NADH-Glo™ Assay solution (50 μL) were added to each well and the plate was placed on an orbital shaker for 2 minutes and subsequently incubated 10 minutes at room temperature. Luminescence was measured using the Promega GloMax®-Multi Detection System with using the provided protocol for luminescence readout.

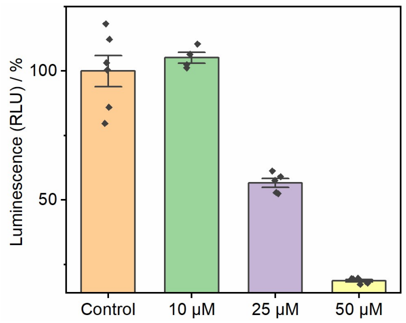

Supplementary Fig. 58| NAD/NADH assay MDA-MB-231 cells treated with different concentrations of 1·TAT for 4 h. A concentration-dependent effect of **1·TAT** on NAD/NADH pathway is observed. Values are normalized towards the untreated control group. Data are presented as mean ± s.e.m., n = 6.

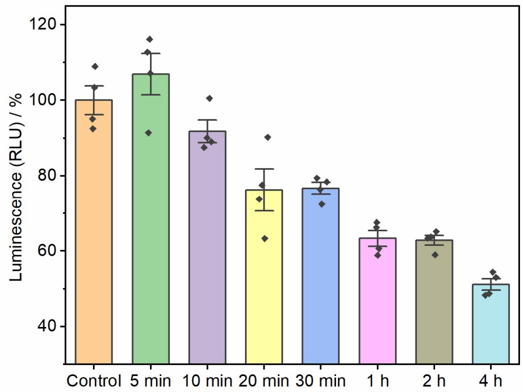

Supplementary Fig. 59| NAD/NADH assay MDA-MB-231 cells incubated with 1·TAT (25 μM) for 5 min, 10 min, 20 min, 30 min, 1 h, 2 h, 4 h. A time-dependent effect of **1·TAT** on the NAD/NADH pathway is observed. Values are normalized towards the untreated control group. Data are presented as mean ± s.e.m., n = 4.

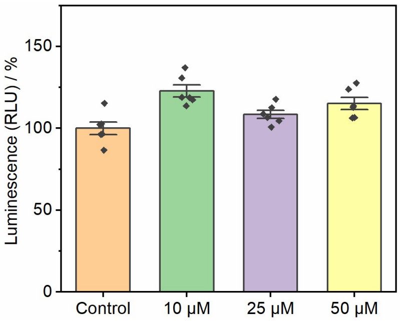

Supplementary Fig. 60| NAD/NADH assay MDA-MB-231 cells treated with different concentrations of 3·TAT for 4 h. 3·TAT shows no significant influence on NAD/NADH pathway at 10 - 50 µM. Values are normalized towards the untreated control group. Data are presented as mean ± s.e.m., n = 6.

7.3 Effect on cellular oxidative phosphorylation

The effect of **1** and **1·TAT** on cellular oxidative phosphorylation of MDA-MB-231 was investigated using the Agilent Seahorse XFe96 Analyzer. Cells were seeded one day before the assay and incubated overnight at 37°C, 5% CO_2. The cartridge for sample loading was loaded with calibration solution (200 µL per well) and incubated at 37°C in a non-CO_2 incubator. On the day of the assay, XF DMEM, pH 7.4 was supplemented with glutamine, glucose and pyruvate regarding to the protocols. Cells were washed once with XF DMEM (200 µL) and the medium was substituted with fresh XF DMEM (180 µL) and placed in a 37°C non-CO_2 incubator for 45 to 60 minutes. The cartridge was prepared by loading the samples (20 µL) in Port A of each well and the components of the performed assay in Ports B-D. The cartridge and 96-well plate with the cells were loaded in the instrument and the components of the ports were added to the cell medium, while the pH, respectively the extracellular acidification rate (ECAR) and the oxygen concentration, respectively the oxygen consumption rate (OCR) was measured.

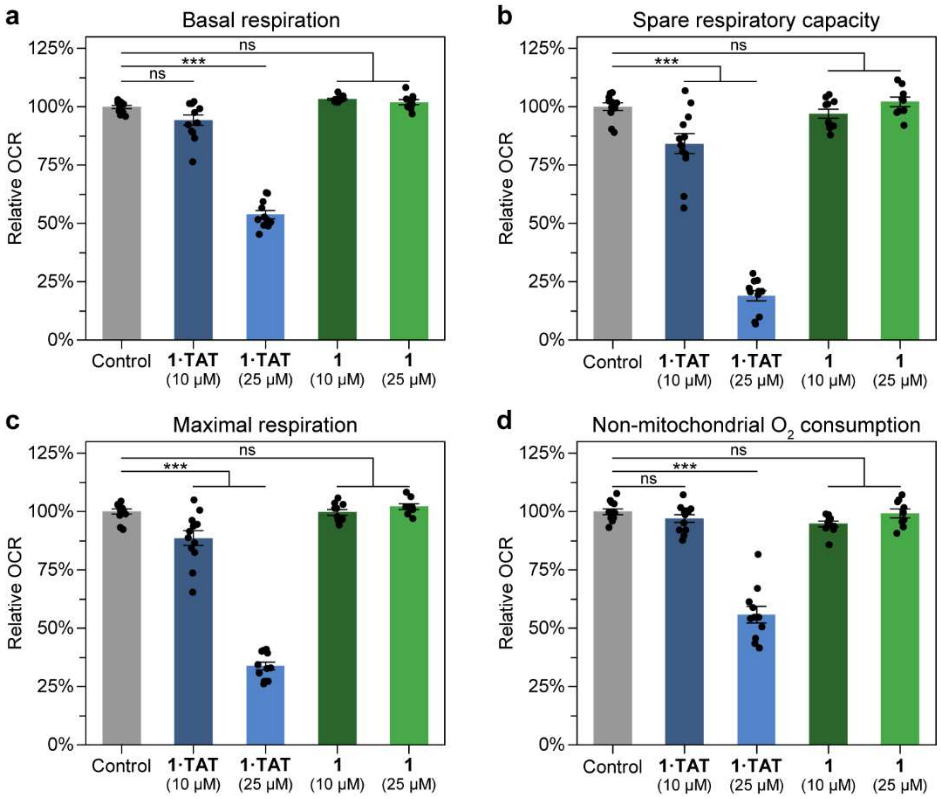

Supplementary Fig. 61| Comparison of 1·TAT (10 µM and 25 µM) and 1 (10 µM and 25 µM) incubation on key cellular Oxphos parameters after incubation for 1 h. a, Basel respiration. **b**, Spare respiratory capacity. **c**, Maximal respiration. **d**, Non-mitochondrial oxygen consumption. Values are normalized towards the untreated control group. The last measurement before the compound injection is set as 100%. Data are presented as mean ± s.e.m., n ≥ 9. Statistical significance was calculated by ANOVA with a Tukey post hoc test. *p < 0.05, **p < 0.01, ***p < 0.001.

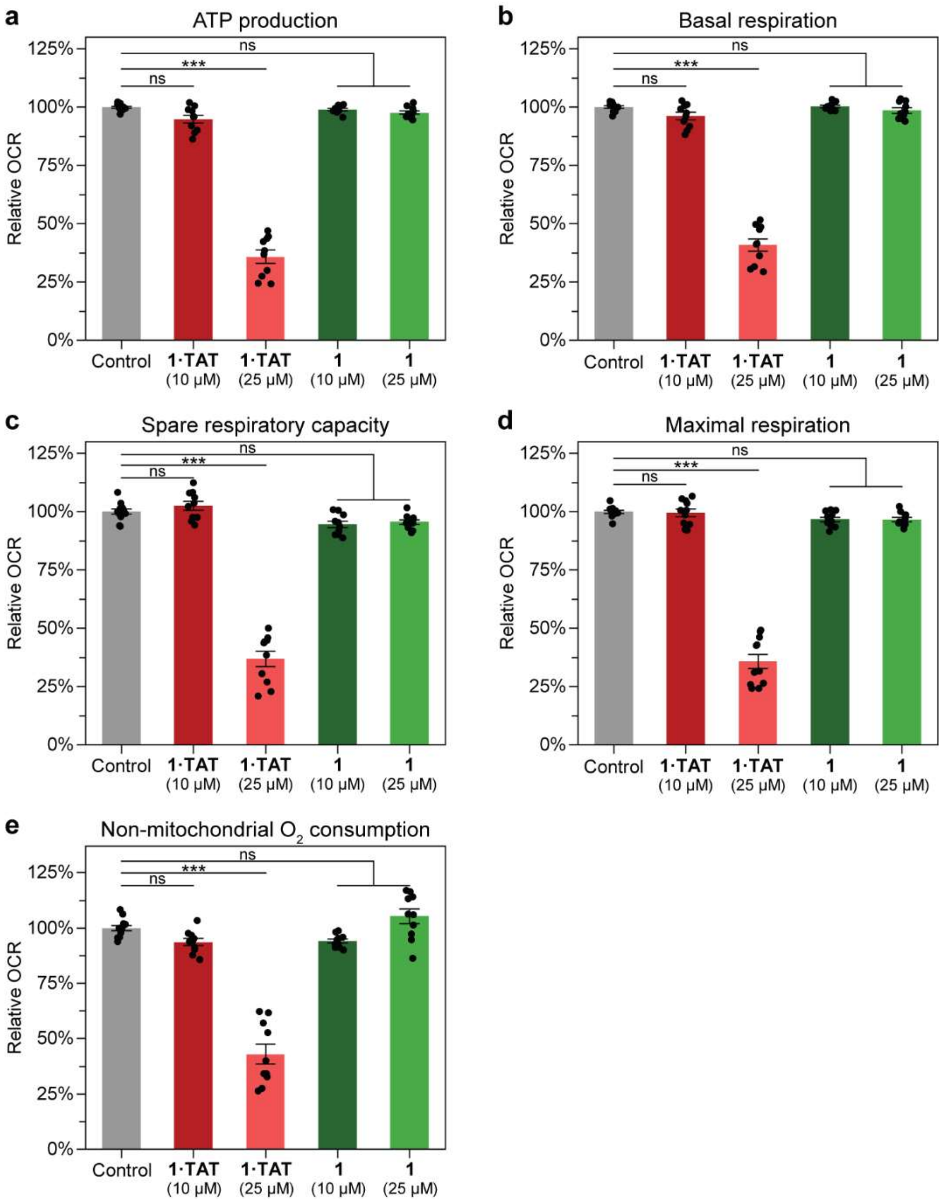

Supplementary Fig. 62| Comparison of 1·TAT (10 µM and 25 µM) and 1 (10 µM and 25 µM) on key cellular Oxphos parameters after incubation for 3 h. **a**, ATP production. **b**, Basel respiration. **c**, Spare respiratory capacity. **d**, Maximal respiration. **e**, Non-mitochondrial oxygen consumption. Values are normalized towards the untreated control group. The last measurement before the compound injection is set as 100%. Data are presented as mean ± s.e.m., n ≥ 9. Statistical significance was calculated by ANOVA with a Tukey post hoc test. *p < 0.05, **p < 0.01, ***p < 0.001.

7.4 Cell viability assay

MDA-MB-231 cells were both seeded at a density of 2,500 cells/well in a white half area 96-well plate and incubated at 37°C overnight to allow them to adhere. On the next day, different concentrations of the **1·TAT** or **3·TAT** were prepared in PBS at a 10x concentration of the final concentration and of this solution (20 µL) were added to fresh medium (180 µL). The old medium of the cells was removed and substituted with fresh medium (50 µL) containing different concentrations of sample, prepared as described before. For each condition triplicates were performed. Cells were incubated with the sample for 4 h or 24 h. After the treatment, CellTiter-Glo Assay solution (50 µL) were added to each well and the plate was placed on an orbital shaker for 2 minutes and subsequently incubated 10 minutes at room temperature. Luminescence was measured using the Promega GloMax®-Multi Detection System with using the provided protocol for luminescence readout.

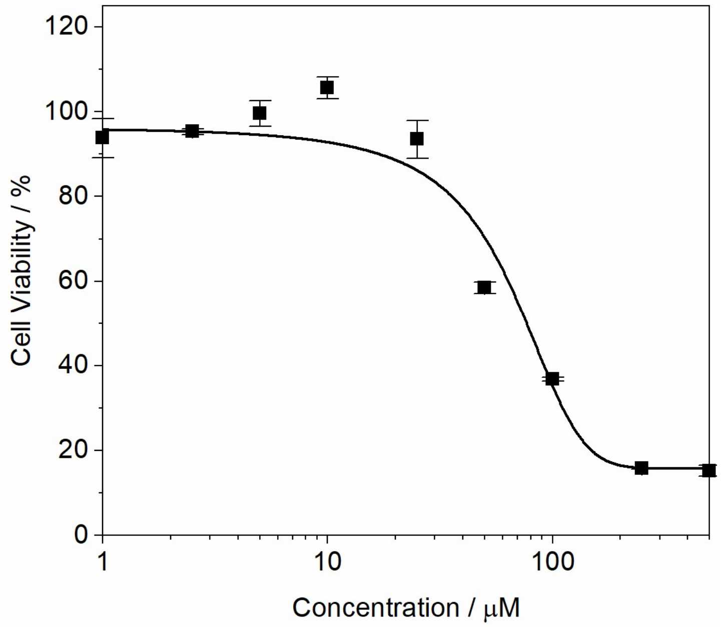

Supplementary Fig. 63| Cytotoxicity assay for MDA-MB-231 cells treated with different concentrations of **1·TAT** for 4 h. The IC50 value is determined to be 69 ± 5 µM. Values are normalized towards the untreated control group. Data are presented as mean ± s.e.m., n ≥ 4.

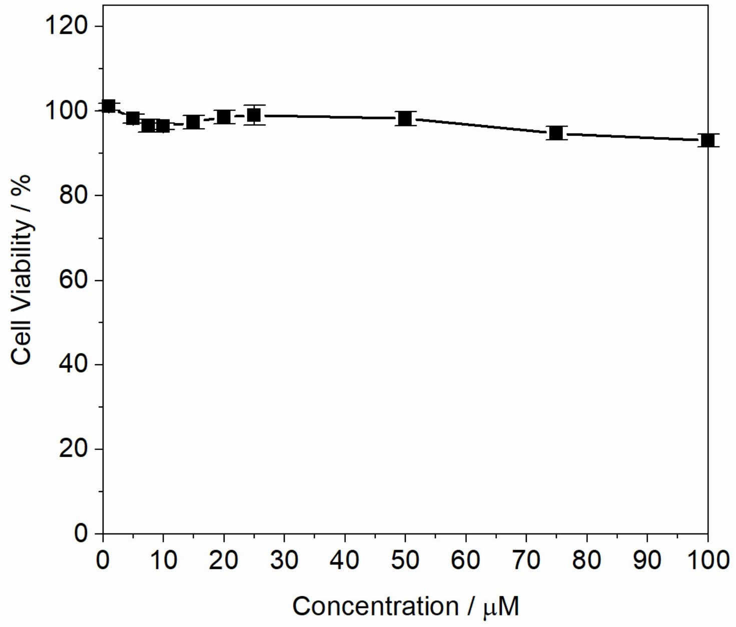

Supplementary Fig. 64| Cytotoxicity assay for MDA-MB-231 cells treated with different concentrations of **3·TAT** for 4 h. **3·TAT** shows no cytotoxicity up to 100 µM. Values are normalized towards the untreated control group. Data are presented as mean ± s.e.m., n ≥ 4.

7.5 Autophagy assay

MDA-MB-231 cells were seeded at a density of 10,000 cells/well in a flat-bottom 96-well plate and incubated at 37°C overnight to allow for adherence. The following day, the cells were treated with **1·TAT** at concentrations of 10 µM and 25 µM for durations of 0.5 h and 1 h. After the incubation period, the medium was aspirated from the wells, and 100 µL of the Autophagosome Detection Reagent working solution was added to each well. The cells were then incubated at 37°C in an atmosphere containing 5% CO_2 for 30 minutes. Subsequent to this incubation, the cells were washed three times with Wash Buffer by gently adding 100 µL of the buffer to each well. The fluorescence intensity, with an excitation wavelength (λ_{ex}) of 360 nm and an emission wavelength (λ_{em}) of 520 nm, was measured using a microplate reader.

7.6 Annexin V/DAPI apoptosis assay

The apoptotic nature of cell death was investigated using Annexin V staining after incubation of MDA-MB-231 cells with **1·TAT** at 25 µM. MDA-MB-231 cells were seeded in an 8-well Ibidi plate at a density of 30,000 cells/well the day before the assay. They were then incubated overnight at 37°C to allow for adherence to the surface. The following day, the cells were treated with **1·TAT** for 4 hours. Post-treatment, they were incubated with Annexin-V FITC, obtained from the Apoptosis Detection Kit FITC by Invitrogen. After this step, the cells were washed once with binding buffer (200 µL). Then, cells in each well were exposed to Annexin V red (5 µL) in binding buffer (200 µL) and incubated for 15 minutes in the dark. Once the Annexin V staining was complete, the cells were washed with binding buffer (200 µL). The cell nuclei were subsequently stained using 4',6-diamidino-2-phenylindole (DAPI) in binding buffer (200 µL), incubating the cells for 5 minutes in the dark. After the staining, the solution was removed, and the cells were washed twice with binding buffer (200 µL each time). The washing

solution was replaced with fresh binding buffer (200 μL) before imaging using confocal laser scanning microscopy (CLSM).

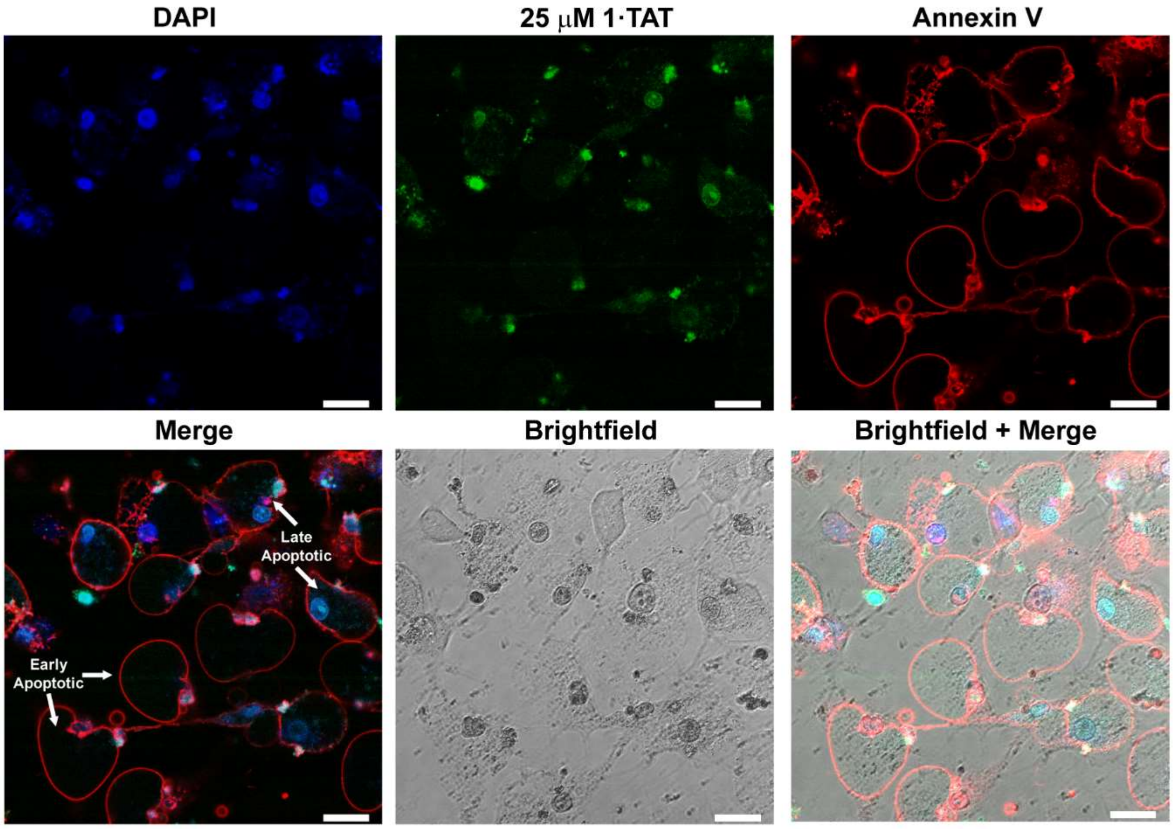

Supplementary Fig. 65| Annexin V/DAPI assay on MDA-MB-231 cells treated with 1·TAT (25 μM). The cells were treated with Annexin V red and DAPI after the incubation. Early apoptosis is characterized by annexin V binding but are not yet permeable towards DAPI, whereas late apoptosis/early necrotic cells show both positive annexin V binding and DAPI permeability. Representative early apoptotic and late apoptotic cells were identified by white arrows. Scale bar = 20 μm.

References

1. Ranjit, S., Malacrida, L., Jameson, D. M. & Gratton, E. Fit-free analysis of fluorescence lifetime imaging data using the phasor approach. *Nat. Protoc.* **13**, 1979-2004 (2018).
2. Berezin, A. A., Sciutto, A., Demitri, N. & Bonifazi, D. Rational Synthesis of AB-Type N-Substituted Core-Functionalized Naphthalene Diimides (cNDIs). *Org. Lett.* **17**, 1870-1873 (2015).
3. Moyer, T. J. *et al.* pH and Amphiphilic Structure Direct Supramolecular Behavior in Biofunctional Assemblies. *J. Am. Chem. Soc.* **136**, 14746-14752 (2014).
